# Melanosome evolution shaped sensory biology and terrestrial adaptations in early aquatic vertebrates

**DOI:** 10.1038/s41467-026-78877-z

**Published:** 2026-10-27

**Authors:** Daniel J. Cirtina, Beatriz Carazo Del Hoyo, Shaun P. Collin, Maria E. McNamara

**Affiliations:** 1 School of Biological, Earth and Environmental Sciences, University College Cork, Cork, Ireland; 2 Sustainability Institute, University College Cork, Cork, Ireland; 3 School of Agriculture, Biomedicine and Environment, La Trobe University, Bundoora, VIC Australia

**Keywords:** Palaeontology, Organelles, Palaeoecology

## Abstract

The photic environment is a key driver of fish ecology that shapes adaptations to aquatic habitats. The evolutionary history of these adaptations, however, is difficult to trace. Melanosomes, melanin-rich organelles, offer unique insights into physiology and ecology and often fossilise. Here, we present a comparative analysis of melanosomes from extant and fossil fishes to trace the evolution of ecological and physiological adaptations through deep time. Melanosomes from internal tissues of extant fishes exhibit highly conserved geometries in all major taxonomic groups examined, indicating persistent functional constraints during evolution. Larger, more abundant melanosomes in lungfish likely reflect adaptations that underpin the vertebrate water-to-land transition. In contrast, eye melanosomes exhibit lineage-specific patterns. Bony fishes possess cylindrical eye melanosomes associated with retinomotor movements, photoreceptor protection, and improved visual acuity, whereas jawless and cartilaginous fishes possess only spheroidal melanosomes, consistent with visual sensitivity and alternative light-regulation strategies including pupillary modulation. These taxonomic differences are preserved in fossils from the late Carboniferous Mazon Creek biota, indicating that links between melanosome morphology and function were established during the early evolutionary history of these lineages in the Ordovician, approximately 445 million years ago. Early innovations in melanin biology uniquely facilitated vertebrate sensory evolution and ecological diversification.

## Abstract (web summary)

Light influences how fishes sense their environment, but tracking the history of visual adaptations has been difficult. This study finds that melanosomes in modern and fossil fishes provide insights into early vertebrate sensory evolution.

## Introduction

Fishes are the most diverse vertebrate group, occupying numerous aquatic niches that vary in temperature, pressure, salinity, oxygen and light intensity^1^. Discrete aquatic light habitats include the epipelagic (sunlight) zone with intense, broad-spectrum light, the mesopelagic (twilight) zone with low-intensity, medium-wavelength light, and the abyssopelagic (abyssal) zone devoid of sunlight^2^. Consequently, vision in fish has evolved under strong selective pressures, yielding various anatomical innovations tailored to specific light regimes^3^.

Many visual adaptations relate to the retina, including ultraviolet-sensitive photoreceptors in many reef-associated lineages^4^, specialisations for aerial vision in various surface-dwelling species^5^ and a multibank retina and retinal diverticula in numerous deep-sea species^6,7^. Sharks (Selachii) have only one type of cone photoreceptor and are considered colour blind^8^, with many showing adaptations that maximise visual sensitivity (i.e. the ability to detect light) and contrast (i.e. the ability to detect differences in brightness). By comparison, bony fishes (Osteichthyes) can possess up to four cone types and vary widely in visual acuity (i.e. the sharpness of visual detail) and sensitivity^9^.

Light modulation mechanisms also differ among major taxonomic groups. Cartilaginous fish (Chondrichthyes) predominantly employ pupillary light reflexes (PLRs, i.e. changes in pupil size), whereas ray-finned fish (Actinopterygii) typically rely on ocular filters^10^ and retinomotor movements (RMMs, i.e. active repositioning of photoreceptors and pigment within the retina)^11^. RMMs protect photoreceptors from excess light exposure under changing ambient illumination^12^, consistent with an alternative evolutionary solution to PLRs in fluctuating light environments and underscoring divergent visual adaptations in Actinopterygii and Chondrichthyes.

Complementing these retinal mechanisms is melanin^13^, a widespread pigment packaged into organelles called melanosomes. In skin and internal tissues, melanin functions in photoprotection^14^, immunity^15^ and metal homeostasis^16^, while in eye tissues it also reduces light scattering and shields photoreceptors from excess illumination^17,18^. Melanosome geometry varies among tissues^19^; the functional implications of this variation are only partially resolved, and the physiological and ecological controls on melanosome geometry in fish have not been systematically investigated.

**Fig. 1:**
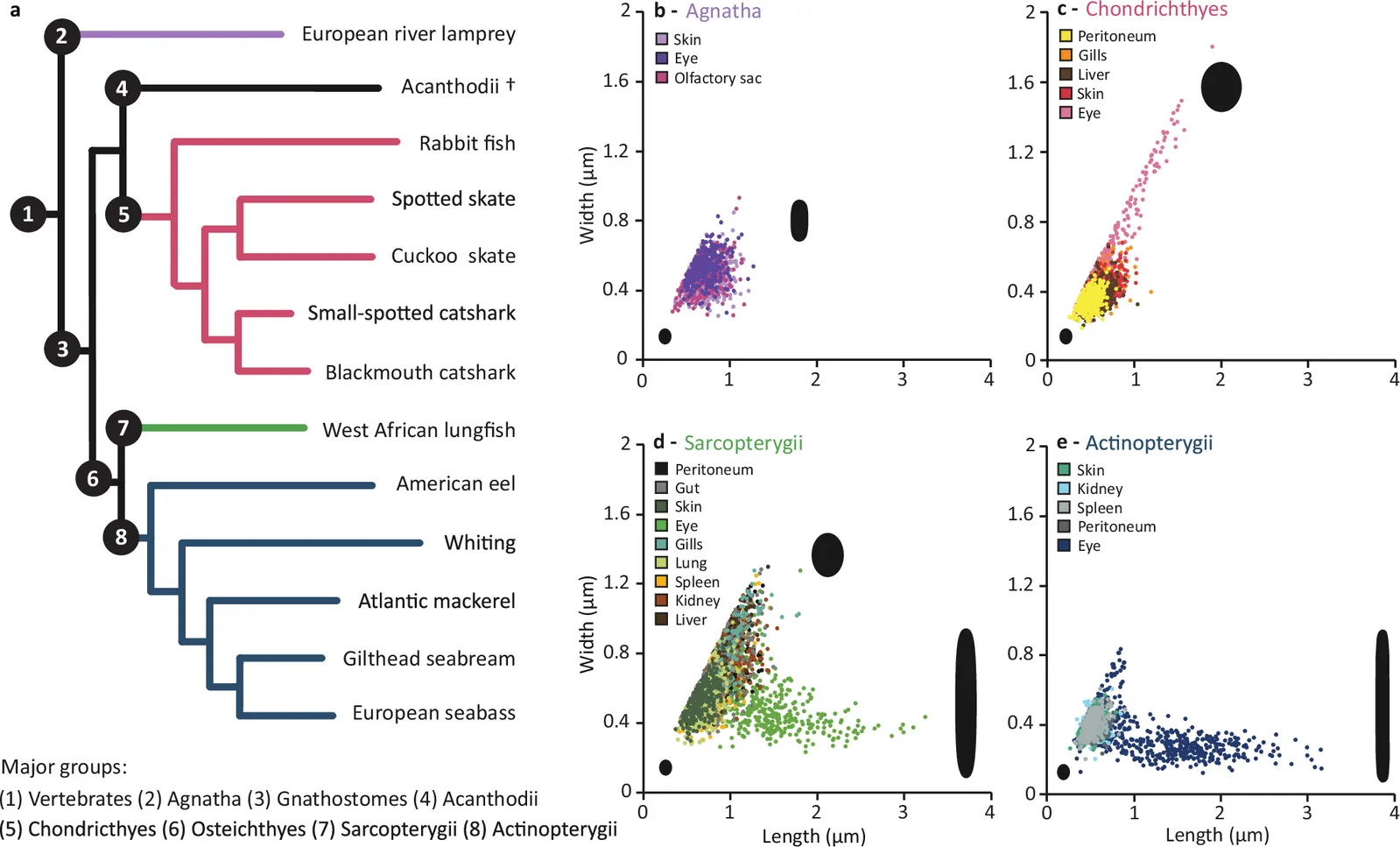
Melanosome geometry in major fish clades. **a** Phylogeny of extant taxa included in this study. Branch numbers represent major groups. **b–e** Scatter plots of melanosome length versus width for various tissues in representative species for major extant fish groups. **b** European river lamprey (*Lampetra fluviatilis*). **c** Blackmouth catshark (*Galeus melastomus*). **d** West African lungfish (*Protopterus annectens*). **e** Whiting (*Merlangius merlangus*). Three biological replicates were analysed for each tissue. Silhouettes represent extremes of melanosome geometry for each group. See Supplementary Fig. 1 for all other species.

To address this, we examine melanosomes from the eye, skin and internal tissues of jawless (Agnatha), cartilaginous (Chondrichthyes), ray-finned (Actinopterygii) and lobe-finned (Sarcopterygii) fishes (Supplementary Tables 1 and 2). Our data reveal lineage- and tissue-specific variation in melanosome size and shape reflecting ecological adaptations and functional specialisations (Fig. 1 and Supplementary Fig. 1). By integrating these data with melanosome measurements from fossil specimens (Supplementary Fig. 2 and Supplementary Table 3), we demonstrate that patterns of variation in melanosome geometry in extant fish have deep evolutionary origins.

## Results

### Anatomical distribution of melanosomes in extant fishes

Enzymatic digestion of various internal and external tissues of extant fishes yielded black to dark brown melanosome extracts. The eyes and skin are melanised in all species, but none show melanosomes in the brain, gonads or heart (Fig. 1 and Supplementary Fig. 1, Supplementary Table 2). The European river lamprey (hereafter river lamprey, Agnatha) exhibits the fewest melanosome-bearing tissue extracts, with melanosomes observed only in the eyes, skin and olfactory sac. The West African lungfish (hereafter African lungfish, Sarcopterygii) is the most heavily melanised taxon, with melanosomes in the gills, eyes, kidney, liver, lungs, peritoneum, skin, spleen and gut.

In the studied Actinopterygii (Atlantic mackerel, European seabass and gilthead seabream, hereafter referred to as mackerel, seabass and seabream, as well as American eel and whiting), melanosomes consistently occur in the eyes, skin, spleen, kidney and, except in seabass, the peritoneum (Fig. 1 and Supplementary Fig. 1, Supplementary Table 2). In contrast, the studied Chondrichthyes (spotted skate, cuckoo skate, blackmouth catshark, small-spotted catshark and rabbitfish) show consistent melanisation only in the eyes and skin. In Chondrichthyes, melanosomes also occur in internal tissues, but their distribution varies among species: they are common in the gills, liver and peritoneum, rare in the gut and spleen, and absent in the kidney. Exceptions include the small-spotted catshark, which lacks melanosomes in the gills and peritoneum, the rabbitfish and blackmouth catshark, which lack melanosomes in the liver, and the spotted skate, which lacks melanosomes in the peritoneum.

Scanning electron microscopy (SEM) images of the spleen, liver and kidney reveal that melanosomes are organised into discrete spheroidal aggregates within melanomacrophage centres (MMCs), with individual aggregates containing tens to tens of thousands of melanosomes (Supplementary Fig. 3). MMC melanosomes are spheroidal (spherical to slightly prolate) with minimal variation in morphology.

In bony fishes (Osteichthyes; Sarcopterygii and Actinopterygii), cylindrical melanosomes (elongate with rounded ends) are present in the retinal pigment epithelium (RPE), where they are oriented parallel to the photoreceptors (Supplementary Figs. 4–6). In contrast, cylindrical melanosomes are absent in chondrichthyans and agnathans (Supplementary Figs. 4, 6). Notably, melanosomes within the RPE of Chondrichthyes are substantially reduced or absent. In all groups, spheroidal melanosomes occur in the choroid and iris, but in Chondrichthyes and Sarcopterygii, melanosomes in these tissues are typically stratified into size-specific layers, with larger melanosomes positioned on the vitreal (inner-facing) side.

### Intraspecific trends in melanosome geometry in extant fishes

We quantified intraspecific variation in melanosomes across extant fishes using standard descriptors of melanosome geometry (length, width and aspect ratio) and assessed the significance of differences among tissues using two-sided analysis of variance (ANOVA) and Tukey-adjusted post hoc pairwise comparisons. We scrutinised pairwise comparisons further using Bhattacharyya coefficients (BCs), which quantify overlap between the distributions for each pair. Higher values indicate greater overlap. BCs were assigned to three categories: low (0–0.33), moderate (0.34–0.66), and high (0.67–1) to facilitate interpretation of morphological differentiation among tissues.

Within individual fish species, melanosome length and width differ significantly among tissues (ANOVA, *p* < 0.05; Supplementary Table 4). Post-hoc comparisons show that these differences are primarily associated with comparisons involving the eyes (Tukey-adjusted *p* < 0.05, Fig. 1 and Supplementary Fig. 1, Supplementary Table 5). Among the skin and internal tissues (gills, kidney, liver, peritoneum, spleen and gut), however, pairwise comparisons are almost all non-significant and BCs are high (Tukey-adjusted *p* > 0.05, BCs = 0.70–0.94; Supplementary Fig. 7 and Supplementary Table 5). Exceptions include the blackmouth catshark and seabream, where comparisons of the peritoneum and gills with other tissues are significant (Tukey-adjusted *p* < 0.05; Supplementary Table 5). Despite differences in these taxa, BCs remain moderate to high (BCs = 0.45–0.89; Supplementary Fig. 7 and Supplementary Table 5), indicating overlapping morphological distributions and suggesting that differences reflect shifts in the central tendency rather than clear morphological separation. The African lungfish is the most striking exception: melanosome length and width in the skin and lung differ markedly from other tissues (i.e., gills, liver, kidney and peritoneum; Tukey-adjusted *p* < 0.05; Supplementary Table 5). BC values for these comparisons are low to moderate, indicating limited morphological overlap (BCs = 0.31–0.65; Supplementary Fig. 7 and Supplementary Table 5). African lungfish melanosomes are smaller in the skin and lung than in other tissues (Supplementary Tables 6, 7).

Melanosome aspect ratio also differs significantly among tissues within individual fish species (ANOVA, *p* < 0.05; Supplementary Table 4). Again, post-hoc comparisons reveal that these differences are primarily associated with comparisons involving the eyes (Tukey-adjusted *p* < 0.05, Fig. 1, Supplementary Fig. 1 and Supplementary Table 5). Pairwise comparisons of melanised tissues in the river lamprey (agnathan) are non-significant and show high BC values, indicating high morphological overlap (Tukey-adjusted *p* > 0.05, BCs = 0.93–0.96; Supplementary Fig. 7 and Supplementary Table 5). In the African lungfish (sarcopterygian), only eye melanosomes differ significantly in aspect ratio from those in other tissues; BCs are low, indicating low morphological overlap (Tukey-adjusted *p* < 0.05, BCs = 0.18–0.31; Supplementary Table 5).

Within the examined actinopterygians and chondrichthyans, only eye melanosomes show significant differences in aspect ratio from melanosomes in the skin and internal tissues; BCs are low to moderate, consistent with limited overlap in melanosome geometry between eyes and other tissues (Tukey-adjusted *p* < 0.05, BCs = 0.11–0.66; Supplementary Tables 4, 5). The blackmouth catshark shows additional significant differences in aspect ratio comparisons involving the gills and peritoneum, while the seabream shows additional significant differences in aspect ratio comparisons involving the peritoneum (Tukey-adjusted *p* < 0.05; Supplementary Table 5). Differences in aspect ratio for the skin and internal tissues (among gills, liver, kidney, spleen and gut) are not significant for Actinopterygii and Chondrichthyes; BCs are typically high, indicating pronounced morphological overlap (Tukey-adjusted *p* > 0.05, BCs = 0.74–0.98; Supplementary Fig. 7 and Supplementary Table 5).

### Interspecific trends in melanosome geometry in extant fishes

**Fig. 2:**
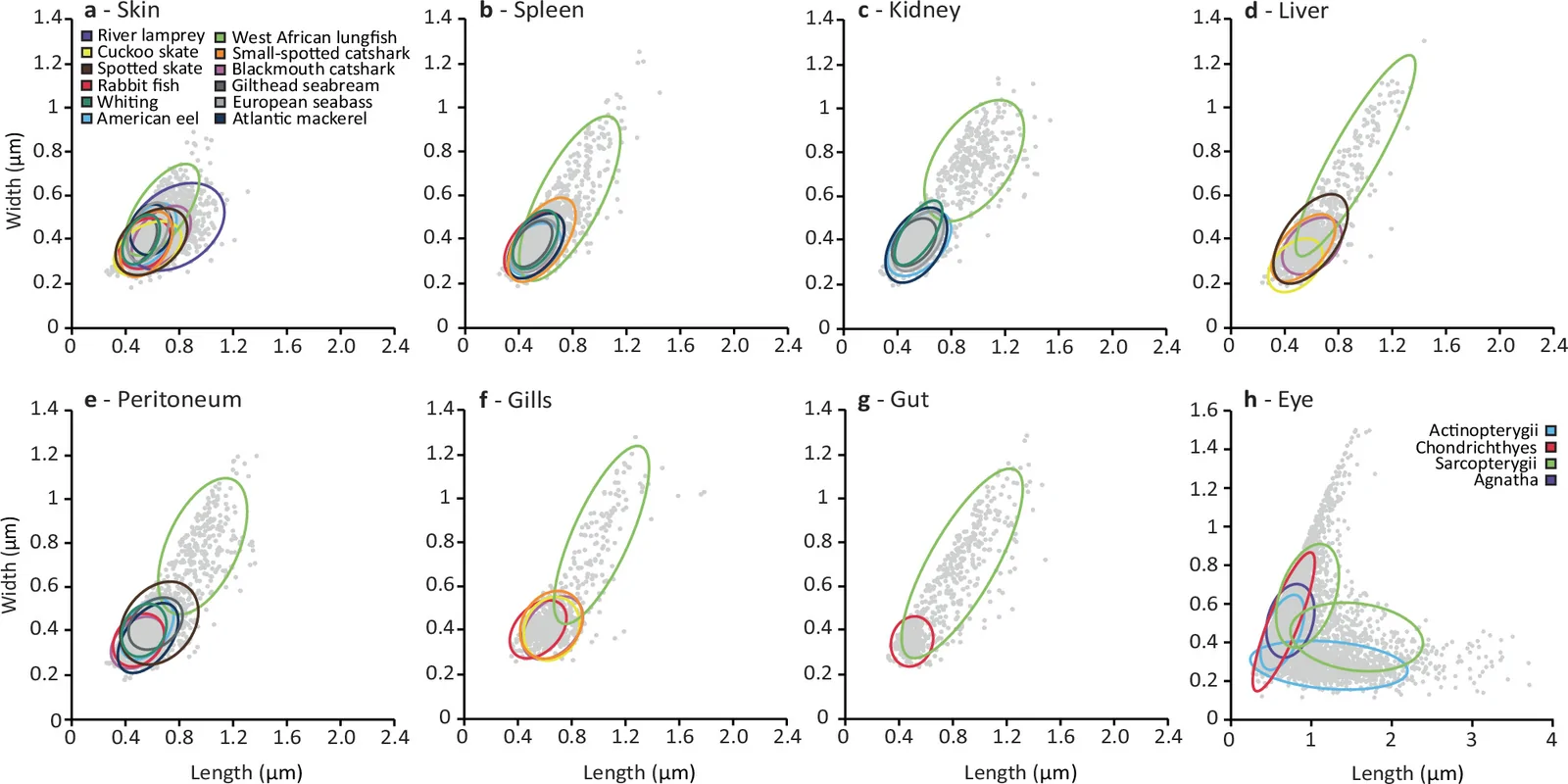
Melanosome geometry in tissues of extant fish. **a–g** 95% confidence ellipses are based on measurements of 300 melanosomes per species, where possible (*n* = 100 melanosomes per each of three biological replicates). For tissues yielding less than 100 observable melanosomes, all visible melanosomes were counted (Supplementary Table 6). **h** 95% confidence ellipses for data from eye tissues use melanosome populations identified by Gaussian mixture modelling (Supplementary Figs. 8, 9) from 600 melanosomes (*n* = 200 melanosomes per each of three biological replicates). Eye data are pooled for each major group. See Fig. 3 for eye data from individual species. Legend in (**a**) applies to (**a–g**).

Analysis of melanosome length and width for individual tissues reveals significant differences among taxa (ANOVA, *p* < 0.05; Figs. 1, 2 and Supplementary Table 8). With few exceptions, significant differences in melanosome length and width within internal tissues (gills, liver, kidney, peritoneum, spleen and gut) are restricted to pairwise comparisons involving African lungfish (Tukey-adjusted *p* < 0.05; Supplementary Table 9). BCs for comparisons including African lungfish (BCs = 0.02–0.52; Fig. 2 and Supplementary Table 9) are lower than those for comparisons excluding African lungfish (BCs = 0.54–0.98; Fig. 2 and Supplementary Table 9), indicating reduced morphological overlap between African lungfish and other taxa. Mean values for melanosome length and width in African lungfish (sarcopterygian) internal tissues are higher than in all other groups (Supplementary Tables 6,  7). In contrast, chondrichthyan and actinopterygian melanosomes show smaller mean lengths and widths, while no melanosomes are reported in the river lamprey (agnathan) for these tissues (Fig. 2 and Supplementary Tables 6, 7).

For skin melanosomes, significant differences in length and width are restricted to pairwise comparisons involving African lungfish, river lamprey and blackmouth catshark; BCs are typically low to moderate, indicating limited morphological overlap among these taxa (Tukey-adjusted *p* < 0.05, BCs = 0.27–0.64; Fig. 2 and Supplementary Table 9). Both agnathan and sarcopterygian species show higher mean, median, IQR, and range values for skin melanosome length and width than Actinopterygii and Chondrichthyes (Fig. 2 and Supplementary Tables 6, 7). River lamprey (agnathan) melanosomes show the lowest mean aspect ratio among all groups (0.62 ± 0.15), whereas African lungfish (sarcopterygian) melanosomes show the highest (0.80 ± 0.12; Fig. 2 and Supplementary Tables 6, 7). Actinopterygii skin melanosomes show a higher mean aspect ratio (0.77 ± 0.11) than Chondrichthyes (0.66 ± 0.14; Fig. 2 and Supplementary Tables 6, 7).

**Fig. 3:**
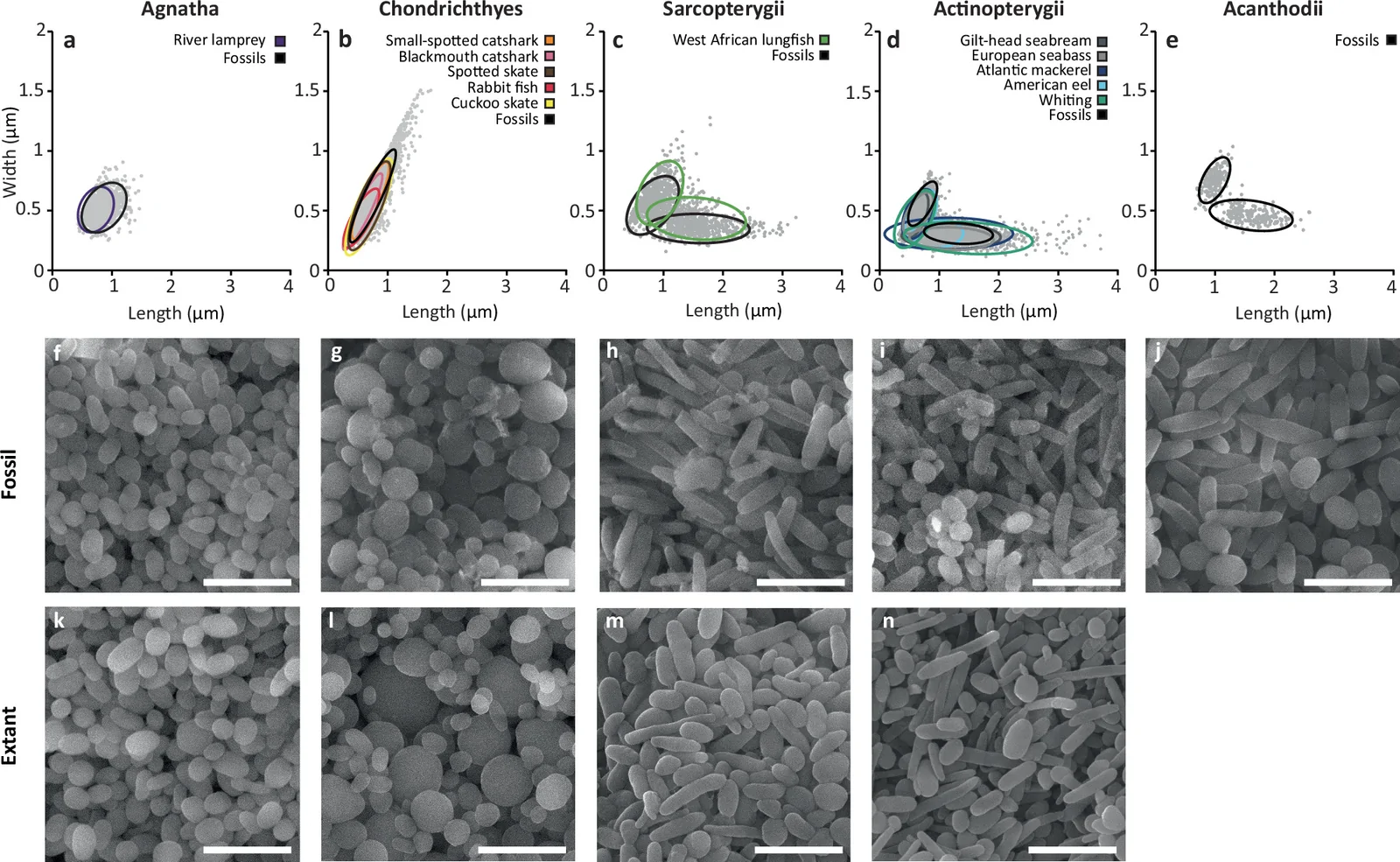
Variation in eye melanosome geometry in major fish groups. **a–e** Scatter plots of eye melanosome geometry for each major group. 95% confidence ellipses use melanosome populations identified by Gaussian mixture modelling (Supplementary Figs. 8–10) from 600 melanosomes (*n* = 200 melanosomes per each of three biological replicates). For fossils, data for each major taxonomic group are pooled for clarity. See Supplementary Fig. 2 for raw measurement data. **f**–**j** SEM images of preserved eye melanosomes in a representative species of fossil fish from each taxonomic group. **f** *Mayomyzon pieckoensis* (Agnatha, PF17992). **g** *Bandringa rayi* (Chondrichthyes, PF10265). **h** *Esconichthys apopyris* (Sarcopterygii, PF8719). **i** *Elonichthys* sp. (Actinopterygii, PF16083). **j** Indet. Acanthodii (PF5754). **k–n** SEM images of eye melanosomes in a representative species of extant fish from each group. **k** European river lamprey (*Lampetra fluviatilis*). **l** Blackmouth catshark (*Galeus melastomus*). **m** West African lungfish (*Protopterus annectens*). **n** Whiting (*Merlangius merlangus*). Scale bars, 2 μm.

**Fig. 4:**
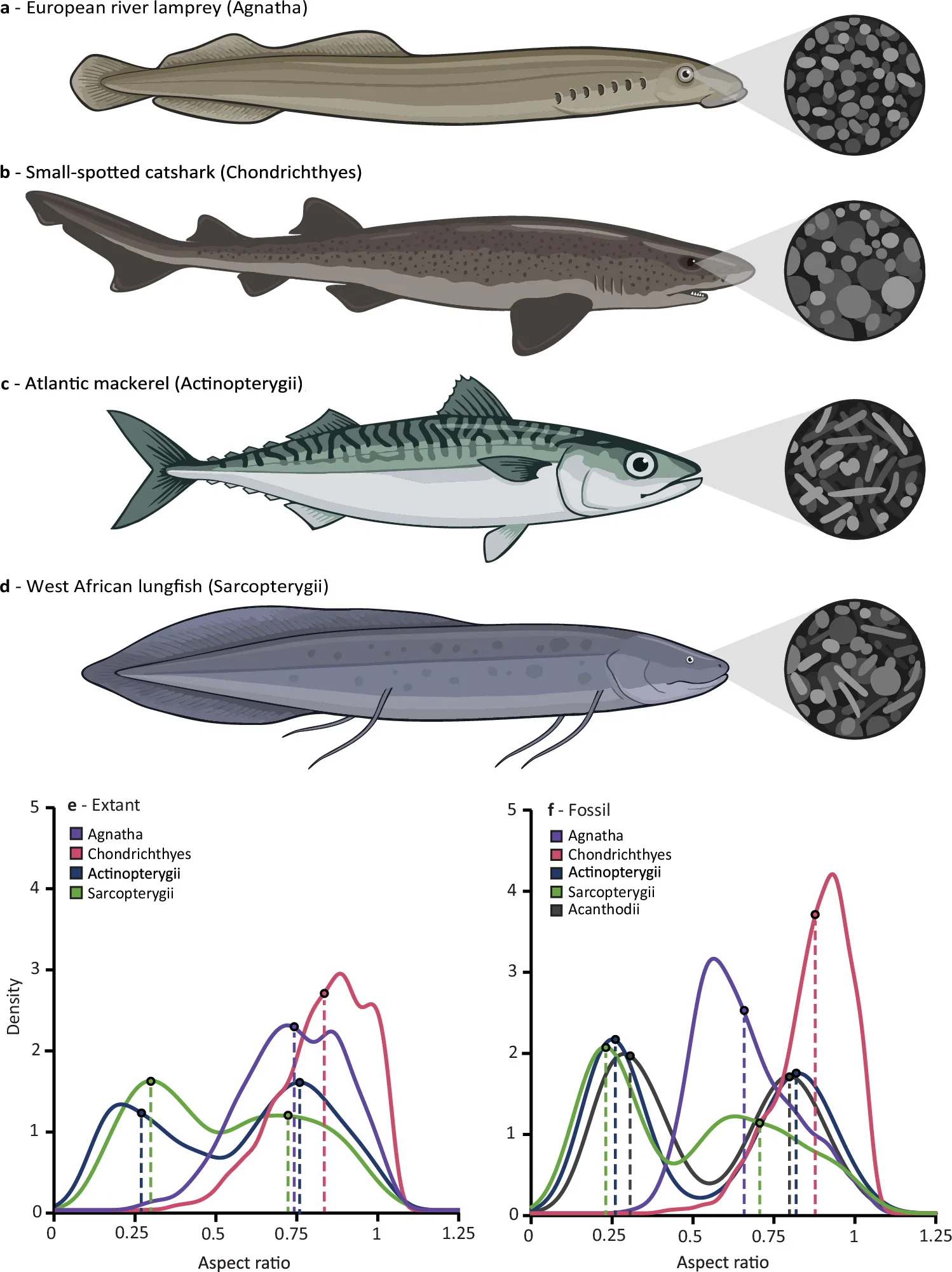
Eye melanosome geometry in extant and fossil fish. **a** European river lamprey (*Lampetra fluviatilis*; Agnatha). **b** Small-spotted catshark (*Scyliorhinus canicula*; Chondrichthyes*)*. **c** Atlantic mackerel (*Scomber scombrus*; Actinopterygii). **d** West African lungfish (*Protopterus annectens*; Sarcopterygii). Density plots for melanosome aspect ratio in extant (**e**) and fossil (**f**) taxa. Dashed lines represent population means. Aspect ratio for melanosomes in extant fish was calculated from length and width measurements obtained from melanosomes isolated from tissues using enzymatic extraction.

Mixture modelling of aspect ratio data reveals that eye melanosomes form one population in Agnatha and Chondrichthyes but form two populations in Actinopterygii and Sarcopterygii (Figs. 3, 4 and Supplementary Figs. 8, 9, Supplementary Tables 10, 11). Population 1 comprises more spheroidal melanosomes, whereas population 2 comprises more cylindrical melanosomes (Supplementary Tables 10, 11).

Analysis of melanosome populations between major extant taxonomic groups reveals significant differences (ANOVA, *p* < 0.05; Supplementary Table 10). Pairwise comparisons of melanosome aspect ratio between populations 1 and 2 are significant among extant groups, with low BCs indicating low morphological overlap (Tukey-adjusted *p* < 0.05, BCs = 8.28 × 10−^3^–0.06; Supplementary Table 11). Population 1 does not differ significantly among groups; BCs are moderate to high (Tukey-adjusted *p* > 0.05, BCs = 0.41–0.86; Supplementary Table 11). Population 2 does not differ between Actinopterygii and Sarcopterygii (Tukey-adjusted *p* > 0.05, BC = 0.39; Supplementary Table 11). Population 1 mean aspect ratios in Agnatha, Chondrichthyes, Sarcopterygii and Actinopterygii are 0.71 ± 0.15, 0.80 ± 0.13, 0.70 ± 0.14 and 0.72 ± 0.14, respectively (Supplementary Table 10). Population 2 mean aspect ratios in Actinopterygii and Sarcopterygii are 0.29 ± 0.09 and 0.26 ± 0.11, respectively (Supplementary Table 10).

### Trends in fossil fish melanosomes

**Fig. 5:**
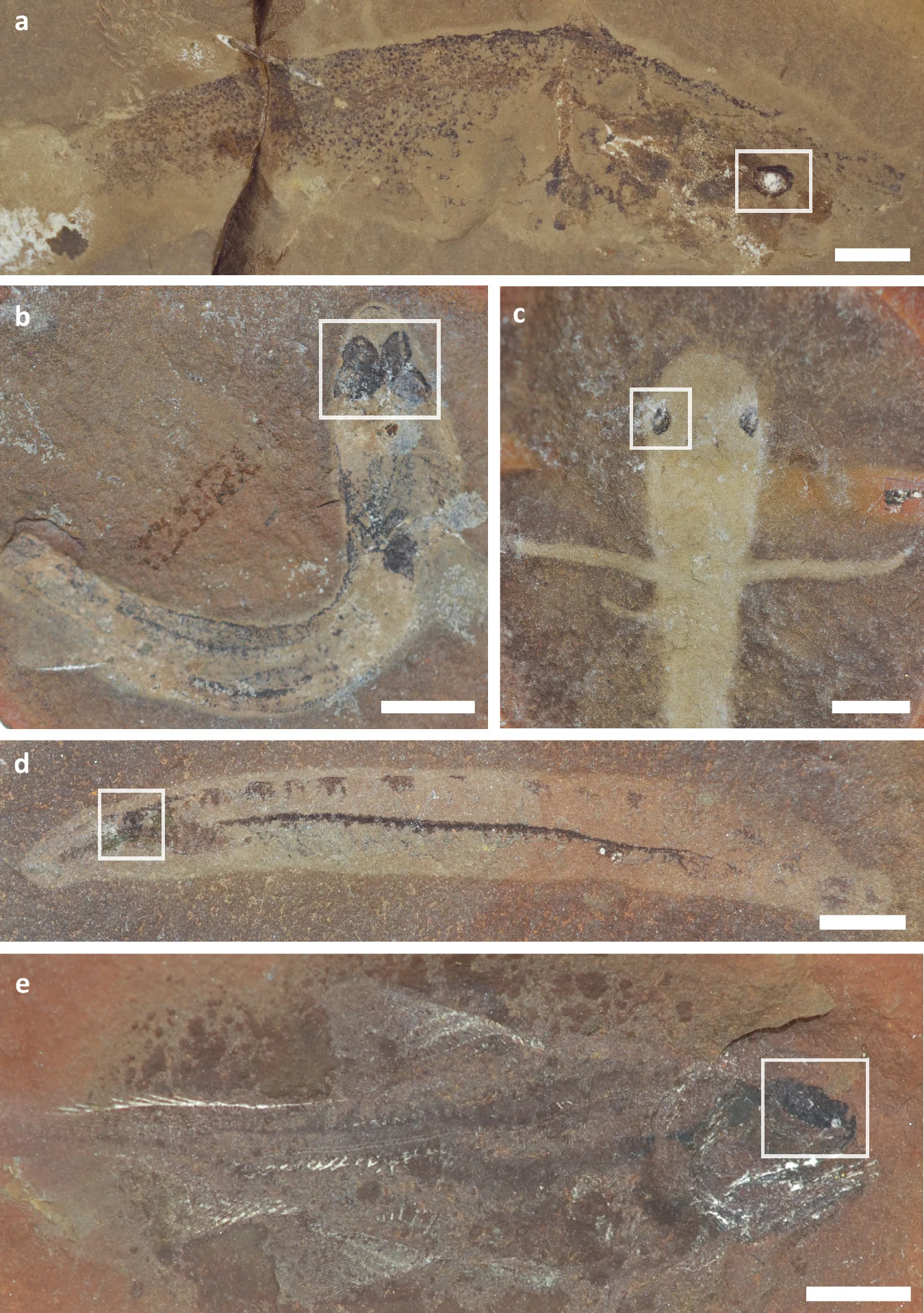
Fossil specimens from the Mazon Creek biota (Late Carboniferous, USA). **a–e** Representative fossils from each taxonomic group. **a** *Bandringa rayi* (Chondrichthyes, PF10265). **b** indet. Acanthodii (PF5754). **c** *Esconichthys apopyris* (Sarcopterygii, PF8719). **d** *Mayomyzon pieckoensis (*Agnatha, PF17992). **e** *Elonichthys* sp. (Actinopterygii, PF16083). White boxes show the preserved eye regions imaged with SEM. See Supplementary Figs. 11–13 for images of all other fossil specimens analysed. Scale bars: (**a–e**) 5 mm.

The examined agnathan, chondrichthyan, acanthodian and actinopterygian fossils from Mazon Creek exhibit skin patterning and evidence of internal organs, defined by preserved melanosomes^20^ (Fig. 5 and Supplementary Figs. 10–13). Examined sarcopterygians, however, lack evidence of melanosomes in skin and internal organs (Fig. 5 and Supplementary Fig. 12).

As in extant fishes, mixture modelling of aspect ratios for fossil eye melanosomes confirms a single melanosome population in agnathans and chondrichthyans, but two populations in Actinopterygii and Sarcopterygii, as well as the extinct group Acanthodii (Figs. 3, 4 and Supplementary Fig. 10). Analysis of fossil melanosome populations between major taxonomic groups reveals significant differences (ANOVA, *p* < 0.05; Supplementary Table 10). Pairwise comparisons of aspect ratio between melanosome populations 1 and 2 across fossil groups are significant and BCs are low, indicating low morphological overlap (Tukey-adjusted *p* < 0.05, BCs = 8.68 × 10−^4^–0.07; Supplementary Table 11). Pairwise comparisons of melanosome aspect ratio for population 1 among all fossil groups are non-significant; BCs are moderate to high (Tukey-adjusted *p* > 0.05, BCs = 0.37–0.92; Supplementary Table 11). Pairwise comparisons of melanosome aspect ratio for population 2 among all fossil groups are non-significant; BCs indicate variable morphological overlap (Tukey-adjusted *p* > 0.05, BCs = 0.33–0.71; Supplementary Table 11). Population 1 mean aspect ratios in fossil Agnatha, Chondrichthyes, Acanthodii, Sarcopterygii and Actinopterygii are 0.63 ± 0.14, 0.84 ± 0.10, 0.77 ± 0.09, 0.68 ± 0.15 and 0.78 ± 0.10, respectively (Supplementary Table 10). Population 2 mean aspect ratios in fossil Acanthodii, Sarcopterygii and Actinopterygii are 0.30 ± 0.08, 0.23 ± 0.06 and 0.25 ± 0.06, respectively (Supplementary Table 10).

Pairwise comparisons reveal no significant differences in melanosome aspect ratio between corresponding fossil and extant populations within each taxonomic group; BCs are moderate to high (Tukey-adjusted *p* > 0.05, BCs = 0.6–0.9; Supplementary Table 11). Further, with few exceptions, pairwise comparisons reveal no significant differences in melanosome aspect ratio between corresponding fossil and extant populations among taxonomic groups; BCs are moderate to high (Tukey-adjusted *p* > 0.05, BCs = 0.48–0.91; Supplementary Table 11).

## Discussion

Our data reveal major trends in fish melanosome geometry that relate to tissue type and phylogeny. Collectively, these trends confirm step changes in melanosome geometry at key physiological, anatomical and ecological transitions of the fish phylogeny, discussed below for the major tissue types analysed.

To date, analyses of fish tissues have only provided direct evidence for eumelanin^21,22^, while reports of pheomelanin^23–27^ have been debated in the absence of direct chemical evidence^28^. Melanin composition was not analysed here, but the dark colour of melanosome extracts in our dataset is consistent with eumelanin-rich melanosomes. These observations are not diagnostic and thus melanosome chemistry is not discussed further. Previous ToF-SIMS analyses of melanosomes from Mazon Creek fossils are consistent with eumelanin^29^.

### Melanosome geometry in the internal tissues of extant fish

Internal tissues in all extant species studied here are characterised by small, spheroidal (spherical to slightly prolate) melanosomes (Figs. 1, 2 and Supplementary Figs. 1, 7, 14). This may reflect optimisation of melanosome geometry under strong functional constraints during early fish evolution, with this geometry retained as a plesiomorphic feature in all groups. Internal melanin in vertebrates is linked to metal sequestration and scavenging of harmful reactive oxygen species (ROS)^30^. In fish, melanin accumulates within the melanomacrophage centres (MMCs) of the liver, spleen, kidney and gills (Supplementary Fig. 3) in response to stress-related stimuli^31^; such functions likely benefit from the high surface-area-to-volume ratio of small, spheroidal melanosomes. Whether these melanosomes accumulate in MMCs through phagocytosis or are synthesised in situ as part of a regulatory response is debated^15^.

African lungfish (Sarcopterygii) show melanisation in more internal tissues and greater variability in melanosome geometry than all other species studied (Fig. 2 and Supplementary Figs. 7, 14). Extensive internal pigmentation has also been reported for *Protopterus aethiopicus*, another species of African lungfish^32^. Given that internal organs in lungfish are largely shielded from direct ambient light, expanded melanosome morphospace in African lungfish internal tissues is more consistent with pervasive differences in physiology rather than differences in light exposure^33^. Further, the high abundance of internal melanin in lungfish is unexpected: in deep-sea fishes, gut and peritoneal melanin is suggested to mask light from ingested bioluminescent prey as camouflage^34^, whereas the shallow freshwater ecology of lungfish suggests an alternative function.

We propose that the strong internal tissue melanisation in African lungfish relates to aestivation, which in lungfish involves terrestrialisation and prolonged metabolic depression^35^. Upon arousal, reoxygenation generates large amounts of ROS that can cause oxidative stress and tissue damage^35^. African lungfish exhibit unique immunological adaptations to counteract this physiological stress, including large granulocyte populations^36^ (which scavenge ROS)^14,15^ and, during aestivation, upregulation of antioxidant defence^37^ and increased tissue pigmentation^36^. Extensive internal melanisation in lungfish may therefore buffer tissues against oxidative stress, raising the intriguing possibility that melanisation of internal organs, by facilitating aestivation, may have played a crucial role in enabling vertebrates to withstand the physiological challenges of transitioning onto land. Expanded melanosome morphospace in lungfish may be a pleiotropic effect of the above and other changes in the melanocortin system^19^.

The role of internal melanin in physiological stress responses is conserved across ectothermic vertebrates^38^. There is also evidence that internal melanin is associated with adaptations to seasonal temperature variation. In some amphibians, increased liver melanisation during seasonal dormancy mitigates low environmental temperatures by leveraging the ability of melanin to absorb energy from photons and convert it to heat^14,39,40^. In African lungfish, however, aestivation occurs in response to drought conditions driven by high temperatures^35^. The extensive internal melanisation in African lungfish is therefore most plausibly explained by ROS-related physiological stress and not as an adaptation to low ambient temperatures.

The lack of internal melanisation in sarcopterygian fossils, relative to other groups (Fig. 5 and Supplementary Figs. 11–13), indicates little pigmentation in vivo, likely reflecting the estuarine setting^41^. In these permanently aquatic habitats, selective pressure for aestivation would have been lower than in extant sarcopterygians inhabiting seasonally dry environments. Limited sampling and preservation bias in the available fossil material, however, cannot be ruled out.

### Melanosome geometry in the skin of extant fish

Superimposed on these broad taxonomic trends are tissue-specific variations in melanosome geometry. In African lungfish, despite pronounced variation in internal tissue melanosome morphology relative to other taxa, skin melanosomes exhibit comparatively limited geometric variation. (Supplementary Fig. 7). This indicates tight functional constraints in skin tissues, likely driven by light-related selection pressures. Further, skin melanosomes are more spherical in Sarcopterygii and Actinopterygii than in Chondrichthyes and Agnatha (Supplementary Table 7). This, in addition to the size^42^ and packing density of melanosomes in skin tissues^43^, provides additional evidence that melanosome size and shape modulate light absorbance and scattering in fish, optimising the optical properties of skin in different species for different lighting regimes. There is no obvious link between the aspect ratio of skin melanosomes and physiological colour change^44,45^; evidence for the latter in several of the studied taxa is limited, preventing assessment of this in our dataset.

In humans, there is evidence that the size of hair melanosomes increases with age, reflecting turnover and replacement by progressively larger melanosomes^46^. There is no evidence for such age-related changes in melanosome size in any tissue in fish. All specimens examined here are sexually mature, minimising potential age-related variation among taxa. Further, to our knowledge, no studies have reported systematic changes in melanosome geometry in response to environmental pH or temperature. As the reported patterns are consistent across groups, these reflect true taxonomic signals rather than a methodological or sampling bias.

### Melanosome geometry in the eyes of extant fish

Our data show strong lineage-specific patterns in fish eye melanosome geometry (Figs. 1–4). In actinopterygians and sarcopterygians (bony fish), the eye contains two distinct melanosome populations discriminated by aspect ratio: cylindrical and spheroidal. In contrast, chondrichthyans and agnathans possess only spheroidal melanosomes (Fig. 4 and Supplementary Figs. 4, 8). These taxonomic differences reflect group-specific visual adaptations. Vertebrates exhibit diverse retinal architectures that balance sensitivity and acuity to meet specific ecological demands, with optimisation of one aspect of visual performance often resulting in trade-offs for others^3^. Species adapted to low light levels typically prioritise sensitivity over acuity, using low ganglion cell densities, high summation (convergence) ratios, high rod-to-cone ratios and/or a tapetum lucidum to maximise photon capture and light detection^3^. We propose that melanosome geometry plays a key role in regulating light and enhancing visual performance, particularly across variable light environments.

In such environments, dynamic control of retinal light absorption relies on pupillary light reflexes (PLR) and retinomotor movements (RMM)^11^. RMMs involve positional adjustment of photoreceptors and melanosomes within the RPE in response to ambient light levels^12^. In bright conditions, rod inner segments extend, pushing the light-sensitive rod outer segments deep into the RPE, while melanosomes migrate apically to surround and shield the outer segments from excess light. Simultaneously, cone inner segments retract, pulling cone outer segments away from the RPE for optimal visual acuity and photoprotection of rods. Under low light, these processes reverse, maximising sensitivity^3,12^ (Supplementary Fig. 6).

Among extant vertebrates, bony fish display the most extensive and widely distributed RMMs^12^, with RMMs in teleosts thought to compensate for the absence of a PLR^47^; these light regulation mechanisms are not mutually exclusive^11,47^. PLR-bearing teleosts are typically bottom-dwellers that use PLRs for camouflage rather than visual adaptation when immersed in the substrate to reduce the contrast between the pupil and surrounding tissue/sediment^11^. RMMs are inherently slow (typically lasting several hours), limiting their effectiveness under rapidly changing light conditions^48,49^. PLRs, however, require only milliseconds to seconds in certain taxa^11^. Further, PLRs are often coupled with dynamic pupil shapes, enabling control of directional light and depth of field adjustments^50^, while most bony fishes retain near-round pupils that constrict less efficiently than slit- or crescent-shaped pupils^11^.

In Actinopterygii, melanosome geometry has been linked to RMM function^17^, whereby only cylindrical melanosomes enter the apical processes of RPE cells^17^ (Supplementary Figs. 4–6). The controls on the formation of elongate melanosomes are thus of particular interest. PMEL (premelanosome protein) is a highly conserved component of melanosome biogenesis in vertebrates and plays a key structural role in the formation of an organised fibrillar scaffold that is, in turn, required for melanosome elongation^51–53^. Inactivation of PMEL in vertebrates prevents the formation of elongate melanosomes^52^. Further, knockdown of PMELa in zebrafish eye tissues results in the loss of melanosome transport into apical processes^17^. The high surface-area-to-volume ratio and reduced cross-sectional area of cylindrical melanosomes are thus thought to enhance melanosome transport by molecular motors through the narrow (<1 µm wide) apical projections^17^. Additionally, the orientation of cylindrical melanosomes parallel to photoreceptors appears to optimise incident light capture and minimise lateral scattering, enhancing overall visual contrast and acuity^17,18,42^.

RMMs have not been reported in Chondrichthyes, consistent with their well-developed PLRs (excluding some deep-sea sharks^8^). The absence of cylindrical melanosomes in Chondrichthyes and the reduction/loss of melanosomes within the RPE (Supplementary Fig. 4)^8,54–56^, therefore, provide new evidence that RMMs are reduced or absent in this group. Given that cylindrical melanosomes may reduce lateral light scatter and enhance acuity^17^, their absence in the retina of Chondrichthyes may be a low-light adaptation, supporting the hypothesis that eye melanosome geometry is linked to lineage-specific visual adaptations. These retinal melanosomes would occlude the tapetum lucidum, impeding light reflection back to photoreceptors and thereby reducing visual sensitivity in these taxa.

Certain chondrichthyans regulate incident light via migration of choroidal melanosomes. Despite this, they retain spheroidal melanosomes. In bright conditions, melanosomes can migrate through the dendritic processes of choroidal melanocytes to occlude the tapetum lucidum and reduce intraocular light scatter^57–59^. Conversely, in dim light, melanosomes retract toward the cell body, exposing the reflective tapetum and enhancing photon capture by retinal photoreceptors^57,58^. Notably, this occurs in few species adapted to brighter conditions; the chondrichthyans studied here are not known to exhibit choroidal melanosome migration, reflecting nocturnal and deep-water behaviour, where visual sensitivity is prioritised^8^. Since the choroid lies sclerad (outward) to the photoreceptors and thus cannot shield them from incoming light, selective pressure to minimise lateral scatter is likely weaker than in the RPE. Additionally, the spacing of guanine crystals may be less tightly constrained than photoreceptor organisation within the retina, imposing fewer geometric constraints on melanosome packing. Consequently, choroidal melanosomes are presumably less constrained by the selective pressures that favour melanosome elongation in the RPE.

Lampreys exhibit RMMs and a PLR^60,61^, although both are considered rudimentary. These RMMs appear to lack melanosome migration and involve myoid elongation in only one photoreceptor type (rod-like short photoreceptor cells^60^), unlike Osteichthyes, where both rod and cone photoreceptors participate^12^. Additionally, the lamprey PLR is mediated solely by melanopsin, independent of photoreceptor input, representing a simpler visual system that predates more complex gnathostome vision^61^. In combination with nocturnality^62,63^ and a reliance on electroreception^64^, lamprey vision appears biased towards sensitivity rather than acuity. Taken together, this rudimentary visual physiology is inferred to have imposed minimal selective pressure for melanosome elongation, explaining the simple spherical to slightly prolate geometry of lamprey eye melanosomes, similar to those within their internal tissues (Figs. 1–3).

Previous studies of the vertebrate eye have indicated that iris pigment epithelium (IPE) and choroidal melanosomes are typically small, spheroidal^65^ and confined to a single layer. Our data, however, reveal that in the examined Chondrichthyes and Sarcopterygii^66^, melanosomes in choroidal and IPE tissues are typically stratified into immobile, size-specific layers, with larger melanosomes positioned on the vitreal (inner-facing) side (Supplementary Fig. 4). This size stratification may modulate light entry and/or intraocular scattering: optimal melanosome sizes for maximum light absorption have been characterised in deep-sea fishes^43^ and numerical modelling indicates that variation in melanosome morphology can alter light scattering and absorption properties^42^. Given the high metabolic and oxidative activity of the choroid^67^, it is likely that choroidal melanosomes contribute to ROS scavenging, but it is not clear how size-based stratification would directly enhance this function.

### Melanosome geometry in the eyes of fossil fish

We analysed eye melanosomes preserved in a range of fossil fishes from the Mazon Creek biota (Carboniferous, USA; Fig. 5 and Supplementary Figs. 2, 11–13). Remarkably, trends in melanosome geometry in the fossil specimens closely mirror those in extant fishes (Figs. 3 and 4, Supplementary Fig. 10). As in extant agnathans^20^, the fossil agnathans possess spheroidal melanosomes with minimal variation in geometry. The fossil chondrichthyans show spheroidal melanosomes of varying size, consistent with the eye melanosome size-based stratification in extant chondrichthyans. The fossil sarcopterygians and actinopterygians show spheroidal to highly elongate, cylindrical melanosomes. These data confirm that the broad taxonomic trends in melanosome geometry identified in extant fishes are deeply conserved and originated at least 306 million years ago. This, in turn, indicates strong conservation of melanosome function across the major fish groups examined. As ocular melanosomes have been identified in early vertebrate eye fossils dating back to the Cambrian^68^, these data provide an avenue for extending phylogenetic analyses deeper into vertebrate evolutionary history.

The lack of significant differences in melanosome size in all fossil groups relative to their extant counterparts (Fig. 3 and Supplementary Fig. 10) is unexpected, given that melanosomes have been shown to shrink during thermal maturation^69^. This raises the possibility that fossil melanosomes were larger in vivo. In humans, eye melanosomes persist throughout life with little to no regeneration^70^; although melanin content decreases with age^71^, melanosome geometry does not change^72^. No comparable data exist for extant fish, but ontogenetic differences are unlikely to explain the possibility of larger in vivo melanosome sizes in the Mazon Creek fishes, as this would require a consistent age bias between fossil and extant taxa, for which there is no ecological or taphonomic basis. If Mazon Creek eye melanosomes were indeed larger in vivo, this may reflect experimentation in early gnathostome visual systems, with subsequent reductions in melanosome size potentially reflecting optimisation for visual function, although the mechanistic basis for such changes remains unresolved.

This dataset provides a framework for classifying fossil fishes that preserve melanosomes and for evolutionary reconstructions of early vertebrate visual ecology. Building on previous qualitative observations of cylindrical and spheroidal eye melanosomes^29^, our approach establishes quantitative analyses of melanosome geometry as a source of taxonomic and ecological information that complements, and is particularly useful, in the absence of traditional anatomical characters.

### Evolutionary Implications

Melanosome geometry in the internal tissues of fishes is highly constrained. With the exception of African lungfish, the internal tissues of fishes from all major groups examined consistently show spheroidal melanosomes with limited variation, indicating that melanosome form is inherently stable across major fish groups in the absence of external light-related selective pressures (e.g., ultraviolet radiation or fluctuating light; Fig. 2, Supplementary Figs. 1, 3 and 7). This strongly conserved geometry suggests an ancestral vertebrate condition, providing a baseline against which differences in geometry can be assessed.

In tissues exposed to light, i.e. eyes and skin^43^, melanosome geometry is more variable among species and eye melanosomes show a strong taxonomic signature for major fish groups (Figs. 1–3). This taxonomic control is best explained by broad ecological differences among major taxonomic groups and indicates adaptations of melanosome geometry in response to selective pressures linked to ambient light levels.

Consideration of the ancestral state for vertebrate vision provides critical context for interpreting the evolution of eye melanosomes^73^. The lamprey eye demonstrates that camera-style vision predates the agnathan-gnathostome split^74,75^. Slight variation in melanosome geometry from spherical to prolate spheroids^20^ (Fig. 3), together with the presence of *pmel* orthologs in agnathans^51^, suggests that experimentation in melanosome elongation began in early vertebrate evolution. The absence of more elongate cylindrical melanosomes in both fossil and extant agnathans (Figs. 3, 4), however, indicates that these more extreme melanosome geometries evolved later, likely driven by ecological demand for enhanced visual performance^20^. The low diversity of melanosome morphologies in agnathans is therefore considered to represent an ancestral condition, consistent with the simplified light-adaptation strategies of the agnathan eye^60,61^.

Our data indicate that cylindrical melanosomes most parsimoniously represent an ancestral condition of gnathostomes, present in actinopterygians, sarcopterygians and stem chondrichthyans (acanthodians), but subsequently lost in crown chondrichthyans (Supplementary Fig. 10). The latter transition may reflect a shift towards visual sensitivity and alternative light-regulatory mechanisms, particularly PLRs (a faster, more flexible light-regulation mechanism than RMMs). These findings imply that complex retinal adaptations (i.e., PLRs and RMMs) were already established by 306 Ma and that RMMs may have been present in the gnathostome ancestor in the Late Ordovician (ca. 445 Ma)^76,77^. When cylindrical melanosomes were lost in Chondrichthyes is less clear, but their absence in all three major extant groups (chimaeras, sharks and rays/skates) suggests a single event at, or close to, the origin of Chondrichthyes in the Early Silurian. Collectively, these findings indicate that taxonomic trends in melanosome geometry in fish have deep evolutionary origins.

The widespread nature of cylindrical melanosomes in fossil Actinopterygii, Sarcopterygii and stem Chondrichthyes (Acanthodii) suggests that RMMs and acuity-related adaptations were the dominant light-regulatory strategies of early gnathostomes. In crown chondrichthyans, however, RMMs were likely reduced or lost as faster, more versatile PLR mechanisms evolved for light regulation^11^, alongside a reduced reliance on melanosomes for intracellular light shielding and increased dependence on sensitivity-enhancing adaptations such as the tapetum lucidum^8,56,78^. This may reflect behavioural changes favouring visual sensitivity and/or ecological expansion into habitats with steep light gradients, in which frequent and rapid vertical movements favoured faster retinal adjustment. In contrast, continued use of RMMs in Actinopterygii is consistent with a greater reliance on acuity-based vision and adaptation to more predictable or gradual changes in ambient light, such as diel transitions, vertical migrations, seasonal changes and occupation of shallow-water environments^79^. This is supported by the high behavioural plasticity (especially in diel activity patterns) of some extant teleosts in response to increased predation^80^.

RMMs and PLRs thus represent alternative visual strategies that balance visual sensitivity, acuity and response speed to changing ambient light levels under selective pressures including social signalling, prey detection and mate recognition^81^. The replacement of RMMs with PLRs in Chondrichthyes represents a pivotal shift from slower, cellular modes of photoprotection to rapid, neural-based reflexes, a transition that likely occurred convergently in multiple vertebrate lineages and that underpins the sophisticated visual systems in modern species.

## Methods

### Modern specimens

In order to test for variation in melanosome geometry across the phylogenetic tree of fish, we identified the following major groupings: Agnatha (jawless fish), Chondrichthyes (cartilaginous fish), Actinopterygii (ray-finned fish) and Sarcopterygii (lobe-finned fish). Groups were selected to correspond with the major phylogenetic groups represented by the fossil taxa. Within these groups, extant fish species were selected to capture representation across different orders, although sampling was constrained by species availability. The taxon phylogeny (Fig. 1a) was constructed following the relationships described in the current literature^82–84^.

Thirty-six sexually mature fish specimens (three biological replicates of each of 12 species) were used for enzymatic melanin extraction (Supplementary Table 1). Specimens of *Chimaera monstrosa* (rabbit fish; Chondrichthyes), *Galeus melastomus* (blackmouth catshark; Chondrichthyes), *Raja montagui* (spotted skate; Chondrichthyes) and *Leucoraja naevus* (cuckoo skate; Chondrichthyes) were caught by the Irish Marine Institute in International Council for the Exploration of the Sea (ICES) subareas 6 (division a, in waters south of 58°N) and 7 (divisions b, c, j and k, in waters west of 8°W). Specimens were frozen at sea, defrosted immediately after transport to the laboratory, and dissected. *Lampetra fluviatilis* (European river lamprey; Agnatha), *Scyliorhinus canicula* (small-spotted catshark; Chondrichthyes) and *Anguilla rostrata* (American eel; Actinopterygii) were purchased online from UK-based commercial suppliers (Gerry’s Fishing, Darwin Biological and Bradley’s Fish, respectively). Specimens were supplied frozen and dissected immediately after defrosting. *Merlangius merlangus* (whiting; Actinopterygii), *Scomber scombrus* (Atlantic mackerel; Actinopterygii), *Dicentrarchus labrax* (European seabass; Actinopterygii) and *Sparus aurata* (gilthead seabream; Actinopterygii) were purchased freshly deceased from local suppliers (K O’Connell’s Fishmongers, English Market, Cork). Specimens were dissected within 24 h after death.

Specimens of *Protopterus annectens* (West African lungfish, Sarcopterygii) were supplied live by Sims Tropical Fish (UK), transported to Ireland by car, euthanised by cranial concussion on arrival at the laboratory and dissected within 12 h.

All research reported here was conducted in accordance with the relevant ethical regulations and guidelines of University College Cork (UCC). African lungfish specimens were imported to Ireland from the UK for scientific purposes under a Movement Approval Notice issued by the Marine Institute Fish Health Unit (OIMP-3346), with entry documented by a Common Health Entry Document for Animals (CHED-A; CHEDA.IE.2023.0004216). Euthanasia was approved by the UCC Animal Experimentation Ethics Committee (approval number: UCC-AEEC-23/001; amendment 10/07/2023) and was performed by Luke Harman (School of BEES; HPRA authorisation AE19130/I087). Wild-caught fish specimens were provided to D.C. by the Marine Institute following collection during the 2023 Irish Anglerfish and Megrim Survey (IAMS; cruise CE23003)^85^, conducted by the Marine Institute in fulfilment of Ireland’s obligations under the Common Fisheries Policy (CFP).

### Fossil specimens

Fossil specimens from different localities inherently differ in their taphonomic history. To minimise potential taphonomic bias among fossil biotas, such as potential melanosome shrinkage during thermal maturation^69^, we limited our fossil dataset to the Mazon Creek Lagerstätte (~306–311 Ma; Moscovian stage, late Carboniferous^86^). This biota was selected as it includes representatives of all four major groups of fish examined in our study (Agnatha, Chondrichthyes, Sarcopterygii and Actinopterygii), plus the extinct stem chondrichthyan group Acanthodii.

We examined melanosomes preserved in the eye region of twenty fossil specimens from Mazon Creek, housed in the collections of the Field Museum of Natural History (FMNH), Chicago, Illinois (Supplementary Table 3). Fossil specimens were accessed through a formal loan from the FMNH (2 May 2025; FMNH invoice no. G-55337), approved by William Simpson and authorised by Arjan Mann. Specimens were temporarily imported to Ireland from the US for study. No import or export permits were required. Four specimens were studied per taxonomic group. Taxonomic identification follows museum designations. Agnathan specimens include three identified as *Mayomyzon pieckoensis* (PF17992, PF5688, PF10788) and one identified as *Gilpichthys greenei* (PF8448). All acanthodian specimens (PF16201, PF5754, PF16263, PF11509) are of undetermined affinity. Sarcopterygian specimens are designated as *Esconichthys apopyris* (PF8719, PF9740, PF9868, PF16850), however, new evidence suggests that they may represent early life stages of other sarcopterygian species^87^. Three chondrichthyans are identified as *Bandringa rayi* (PF10265, PF15328, PF3197) and one as *Bandringa* sp. (PF16367). Among the actinopterygians, two are assigned to *Elonichthys peltigerus* (PF7487, PF7499), and two are *Elonichthys* sp. (PF16084, PF16083). Digital images of specimens were taken using a Nikon D3100 digital SLR camera.

Specimens from the Mazon Creek biota are preserved as well-defined two-dimensional impressions within siderite concretions. The studied specimens locally show a thin coating of white kaolinite, which represents a secondary diagenetic precipitate^20^. All examined dark regions within the fossils correspond to locations of melanosome preservation. Several specimens also exhibit a pale circular structure at the centre of each eye, interpreted as the lens.

Recent analyses of the Mazon Creek deposits, integrating sedimentology^86^ and statistical assessment of over 300,000 siderite concretions^41^, conclude that the site represents a river delta system delivering freshwater and sediment into a shallow, low-energy, brackish marine basin. Within this framework, three distinct biotas have been recognised, each corresponding to a different paleoenvironmental setting: the Braidwood biota is dominated by terrestrial washout flora and fauna in a brackish estuarine environment; the Will-Essex biota supports abundant benthic taxa in a shallow nearshore marine environment; and the Kankakee-Essex biota is inhabited primarily by pelagic and fully marine organisms in a more distal offshore environment^41^.

Specimen PF15382 (*Bandringa rayi*) was recovered from Essex deposits of the Vermilion Shaft Mine in Will County. The exact recovery location of PF10788 (*Mayomyzon pieckoensis*) is unknown. All other specimens in this study (Fig. 5, Supplementary Figs. 11–13) were recovered from Pit 11 in Will County, which contains predominantly marine fauna of the Essex Formation, with benthic organisms more abundant to the north and pelagic organisms more abundant to the south^41^. While all sarcopterygian fossils included in the study were collected from Pit 11 and thus belong to the marine fauna of the Essex Formation, lungfish scales and teeth are also reported from the estuarine Braidwood Formation^41^. The aquatic species of the Braidwood assemblage are considered to have lived predominantly in brackish estuarine habitats, while their presence in the Essex assemblage represents the seaward limit of their living ranges^41^.

### Enzymatic melanin extraction

Eyes, skin, peritoneum, spleen, kidney, liver, gills, gut, gonads, brain and heart were dissected from each fish where possible. The lung was dissected only in African lungfish and the olfactory sac only in river lamprey. This yielded a total of 432 tissue samples across 12 taxa, with three biological replicates per taxon (Supplementary Table 1). Tissues were processed immediately or stored for up to a month at −20 °C prior to further treatment. Melanosomes were extracted using a modified version of Novellino et al.^88^, summarised below.

For each dissected tissue sample, 100 mg of tissue was added to 6 mg of collagenase and 1.5 ml of phosphate buffer solution (pH 7.4) prior to incubation for 20 h at 37 °C and 200 rpm. Samples were then centrifuged at 9000 *g* for 4 min and washed once with 1 ml acetone and three times with 1 ml DDI water, centrifuging at 9000 *g* between each wash. After a second treatment of collagenase and washing, 1 mg DL-dithiothreitol (DTT), 5 mg proteinase K and 1.5 ml of 0.1 M phosphate buffer were added to each sample prior to incubation for 20 h at 37 °C and 200 rpm. Then, 80 µl Triton X-100 was added and vortexed for 10 s prior to incubation for 4 h at room temperature (15–20 °C) at 200 rpm. Samples were then centrifuged at 9000 *g* for 4 min and washed once with 1 ml acetone and three times with 1 ml DDI water, centrifuging at 9000 *g* between each wash. Samples were treated four more times with proteinase K and DTT, with washes between each treatment. Then, 1 ml of 36% HCl was added, and the samples were shaken at 200 rpm for 20 h at room temperature. Samples were washed successively with 1 ml each of ethanol, heptane, and acetone (three times each) and vortexed for 10 s. Samples were centrifuged at 9000 × *g* for 4 min after each wash before adding 1 ml of acetone and centrifuging at 21,000 × *g* for 20 min to pelletise the solid residue. The remaining residue was dried overnight in a fume hood and then transferred to glass vials for storage.

### Scanning electron microscopy

Melanosome extracts were fixed onto aluminium stubs with carbon tape and sputter-coated with Au for 50 s using an Agar Manual Sputter Coater. Fossil specimens were analysed uncoated and were wrapped in foil, leaving only the area of analysis exposed to facilitate imaging under high vacuum. All fossil specimens were examined in their entirety as destructive sampling and gold sputter coating were not permitted. Extracts and fossils were examined with a JEOL JSM-IT100 variable-pressure scanning electron microscope at an accelerating voltage of 15 kV and a probe current of 25 nA. The contrast and brightness of SEM images were adjusted in Adobe Photoshop where necessary.

### Histology

Skin and internal tissues (approximately 3 × 3 mm) were fixed in a solution of 2% glutaraldehyde in 0.1 M phosphate buffer (pH 7.4) for 3 h at room temperature (23 °C). For eye tissues, a 2 × 2 mm window was cut in the eye wall prior to fixation to facilitate penetration^89^. Eyes were fixed in a solution of 1% formaldehyde and 1.25% glutaraldehyde in 0.1 M phosphate buffer for 36 h at room temperature^90^.

After fixation, eyes were cut into three pieces^91^ and processed for paraffin embedding using a Shandon Citadel 1000 automated embedder. Tissues were dehydrated through a graded ethanol series (30%, 50%, 70%, 80% and 2 × 95%; 6 h total), followed by three 2 h washes in 100% ethanol and two 2 h washes in butanol, before infiltration with paraffin (three × 3 h treatments). Paraffin blocks were prepared with a Tissue-Tek TEC 5 console and sectioned at 6 µm using a Leica RM2235 microtome. To facilitate mounting, wax sections were floated on a heated water bath at 40 °C to flatten the section prior to collection on glass slides. Sections were deparaffinised with two 5 m washes in xylene. Sections were left uncovered and imaged using SEM.

### Data processing and statistical analysis

Where possible, the length and width of 100 melanosomes were measured for each sample (totalling 300 across three biological replicates) from digital images using the Fiji distribution package^92^ in ImageJ2^93^ (Supplementary Data 1). For samples with markedly variable melanosome geometries (eye samples), 200 melanosomes were measured to better characterise the full range of morphological variation (totalling 600 across three biological replicates). For tissue yielding fewer than 100 observable melanosomes, all visible melanosomes were measured.

Although melanosomes were observed in the dark, carbonaceous eye region of all twenty Mazon Creek fossils analysed, three specimens lacked sufficient melanosomes for statistical analyses (Supplementary Table 2): one chondrichthyan (PF15382) and two acanthodians (PF16263, PF11509). In fossils with both cylindrical and spheroidal eye melanosomes, melanosomes are often preserved as two distinct layers, each with size-specific melanosome geometry. These layers were treated as representing the retinal pigment epithelium (RPE) and choroid/iris, which are superimposed during fossilisation^20^. A total of 100 melanosomes were measured from each layer (totalling 200 melanosomes per specimen; Supplementary Table 12).

Measurements of melanosome length and width from extant fishes were performed on melanosomes isolated from tissue by enzymatic extraction. Since melanosomes were removed from their in situ cellular context, their orientations on SEM stubs are effectively random rather than constrained by cellular architecture. Likewise, decay and early diagenetic processes in fossils can disrupt original cellular arrangements, leaving preserved melanosomes similarly disordered. SEM images of the melanosome extracts thus contain melanosomes in multiple orientations, reducing the bias inherent to two-dimensional measurements made on TEM sections of in situ tissue. Melanosomes are typically three-dimensional organelles (spheroidal/cylindrical), as demonstrated by electron tomography^94^, and measurements of their length and width therefore provide a consistent approximation of the principal geometric axes used here for comparative morphometrics.

All statistical analyses were conducted in R (version 4.5.0)^95^, with statistical significance assessed using two-sided tests at a threshold of *p* < 0.05. Statistical analyses are reported in Supplementary Tables 4–12. Data manipulation and visualisation were performed with base R, the tidyverse package (version 2.0.0),^96^ and Adobe Illustrator (version 30.8.1). To reduce pseudoreplication, melanosome measurements were first averaged within each biological replicate for each tissue. Differences in melanosome morphology among tissues within species and among species within tissues were assessed using two-sided one-way analyses of variance (ANOVA), with tissue or species, respectively, included as the fixed effect. Model assumptions were assessed using Shapiro–Wilk tests of residual normality, residual diagnostics, and Levene’s tests for homogeneity of variance (car package version 3.1.3^97^). Where significant differences were detected, estimated marginal means were calculated and pairwise comparisons performed with Tukey adjustment for multiple comparisons (emmeans package version 2.0.4^98^).

Mixture modelling was performed to assess the distribution of eye melanosome aspect ratios among species and taxonomic groups (Supplementary Data 1). Gaussian and *t*-mixture models were applied to identify finite mixtures of Gaussian and multivariate *t*-distributions, respectively, and to characterise subpopulations with distinct melanosome geometries (mclust version 6.1.3^99^ and teigen version 2.2.2^100^ packages, respectively). While *t*-mixture models are better suited to heavy-tailed data and outliers, both *t*-mixture and Gaussian mixture models produced nearly identical results. Gaussian models are presented here (Supplementary Figs. 8–10), as mclust in R provides functionality for extracting soft cluster probabilities. The optimal number of populations was determined using the Integrated Completed Likelihood (ICL) criterion. Melanosomes in internal tissues and skin were collectively described here as spheroidal to encompass variation from near-spherical to slightly prolate geometries. Mixture modelling using the ICL criterion did not resolve these subtle geometric differences into discrete populations, and thus they were treated collectively here.

For comparisons of eye melanosome populations identified by Gaussian mixture modelling, melanosome measurements were averaged for each population within each biological replicate. These values were analysed using linear mixed-effects models (nlme package version 3.1-171^101,102^). Observed combinations of taxonomic group, preservation state (extant or fossil) and melanosome population were modelled as the fixed effect, with species included as a random effect to account for variation among species. Models allowing residual variance to differ between melanosome populations 1 and 2 were compared with common-variance models using likelihood-ratio tests. As residual variance differed significantly between populations for length and width, these models were fitted allowing population-specific residual variances; this was not required for aspect ratio. Final models were fitted using restricted maximum likelihood, and the significance of fixed effects was assessed with ANOVA. Model assumptions were evaluated using Shapiro–Wilk tests of residual normality, residual diagnostics, and Levene’s tests for homogeneity of variance (car package version 3.1.3^97^). Where significant differences were detected, estimated marginal means were calculated and pairwise comparisons performed with Tukey adjustment for multiple comparisons (emmeans package version 2.0.4^98^).

To quantify overlap between melanosome population distributions, we computed multivariate Bhattacharyya coefficients (BCs) from the mean vectors and covariance matrices of length, width, and aspect ratio. BC values range from 0 (no overlap) to 1 (complete overlap), providing a quantitative measure of similarity between distributions. Bhattacharyya coefficients (BCs) were assigned to three categories: low (0–0.33), moderate (0.34–0.66) and high (0.67–1), to facilitate interpretation of morphological differentiation among tissues, species and melanosome populations.

### Reporting summary

Further information on research design is available in the Nature Portfolio Reporting Summary linked to this article.

## Supplementary information

Supplementary Information

Description of Additional Supplementary Files

Supplementary Data 1

Reporting Summary

Transparent Peer Review file

## Data Availability

All data generated and analysed during this study are included in this article and its Supplementary Information files. Processed melanosome measurement data are provided in Supplementary Information. Raw melanosome measurements are provided in Supplementary Data 1.
