# Supplementary material for: Melanosome evolution shaped sensory biology and terrestrial adaptations in early aquatic vertebrates
Source: Nat Commun. 2026 Oct 27;17:10958. doi: 10.1038/s41467-026-78877-z (PMC13714305; doi:10.1038/s41467-026-78877-z)
Supplement: Supplementary file 1 [file 41467_2026_78877_MOESM1_ESM.pdf]

Supplementary Information

Melanosome evolution shaped sensory biology and terrestrial adaptations in early aquatic vertebrates

Daniel J. Cirtina*[1, 2], Beatriz Carazo Del Hoyo[1, 2], Shaun P. Collin[3] and Maria E. McNamara*[1, 2]

[1]School of Biological, Earth and Environmental Sciences, University College Cork, Cork, Ireland

[2]Sustainability Institute, University College Cork, Cork, Ireland

[3]School of Agriculture, Biomedicine and Environment, La Trobe University, Bundoora, Victoria, Australia

*Corresponding authors; dcirtina@ucc.ie, maria.mcnamara@ucc.ie

Table of Contents

Supplementary Figures: P2–15

Supplementary Tables: P16–31

Supplementary Figures

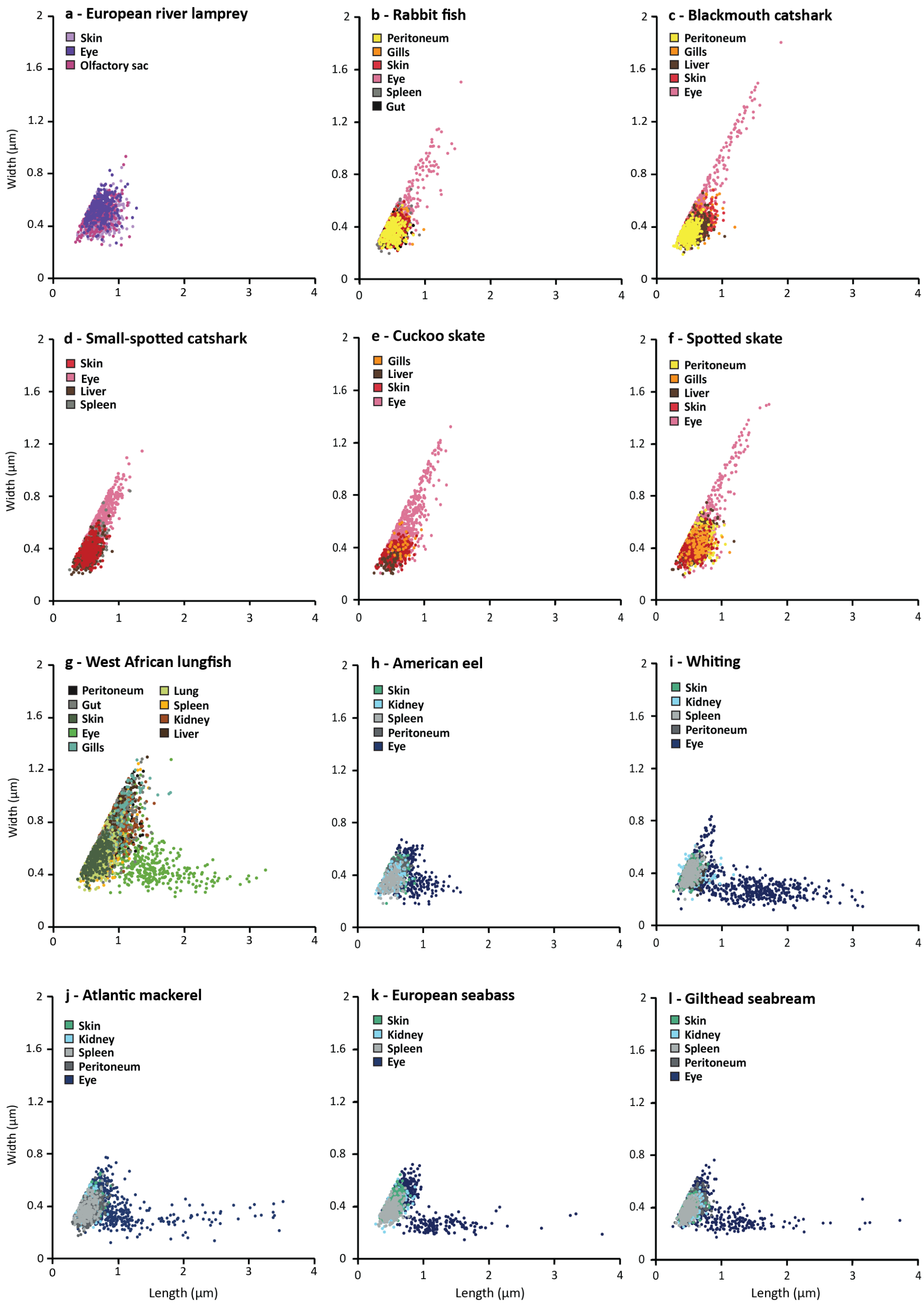

Supplementary Fig. 1 Melanosome geometry in extant fish. a Agnatha. **b–f** Chondrichthyes. **g** Sarcopterygii. **h–l** Actinopterygii. Eye tissues are represented by measurements of 600 melanosomes (n = 200 melanosomes per each of three replicates per tissue). For all other tissues, 300 melanosomes were measured where possible (n = 100 melanosomes per each of three replicates per tissue). For tissue yielding less than 100 observable melanosomes, all visible melanosomes were measured (Supplementary Table 6). Measurements were taken from melanosomes isolated from tissues using enzymatic extraction.

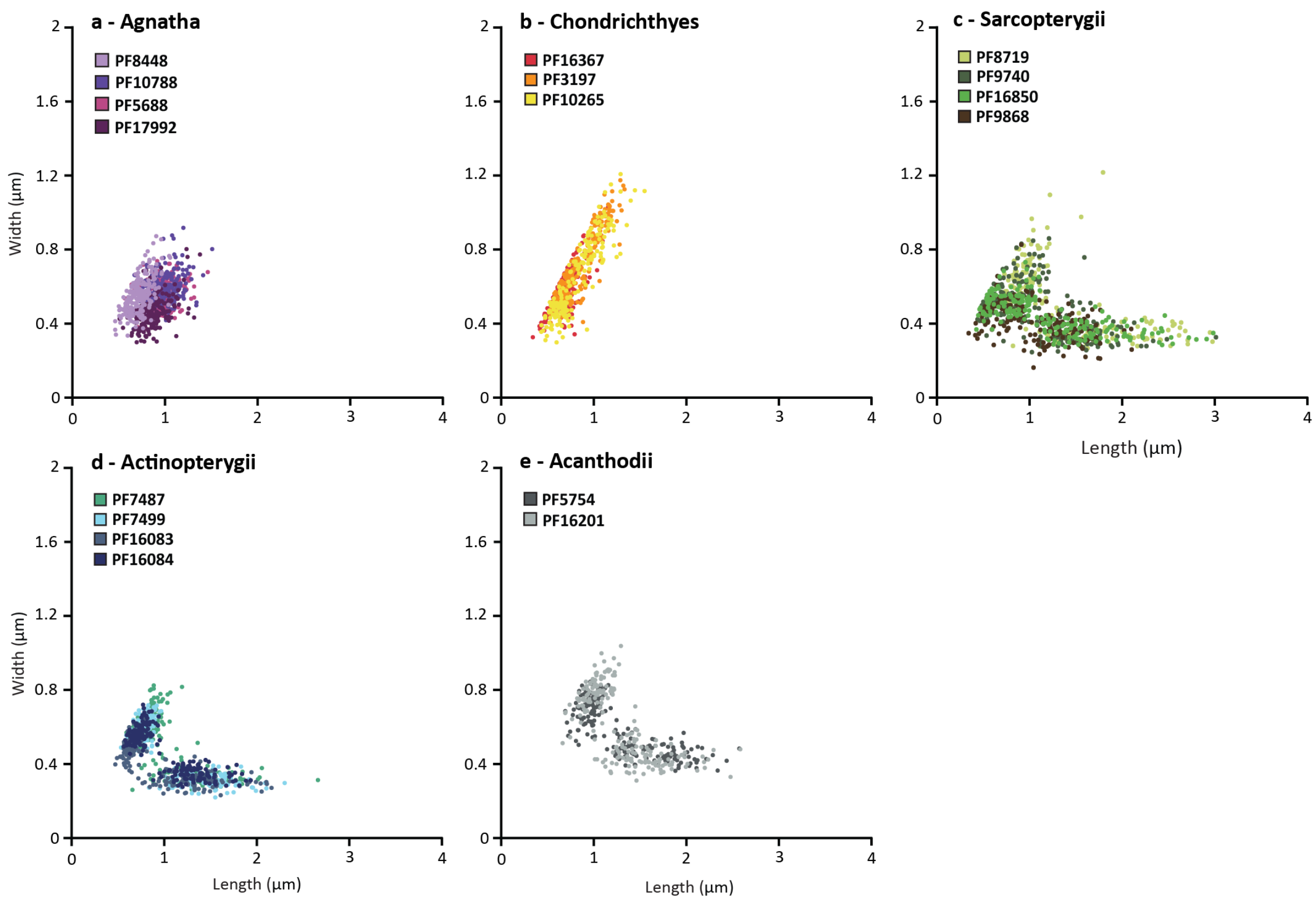

Supplementary Fig. 2 Geometry data for eye melanosomes from fossil fish. a Agnatha. **b** Chondrichthyes. **c** Sarcopterygii. **d** Actinopterygii. **e** Acanthodii All fossil specimens analysed are from the Mazon Creek biota (Late Carboniferous, USA). Each specimen is represented by measurements for 200 melanosomes (Supplementary Table 12). PF numbers denote museum specimen numbers (Field Museum of Natural History, Illinois, USA).

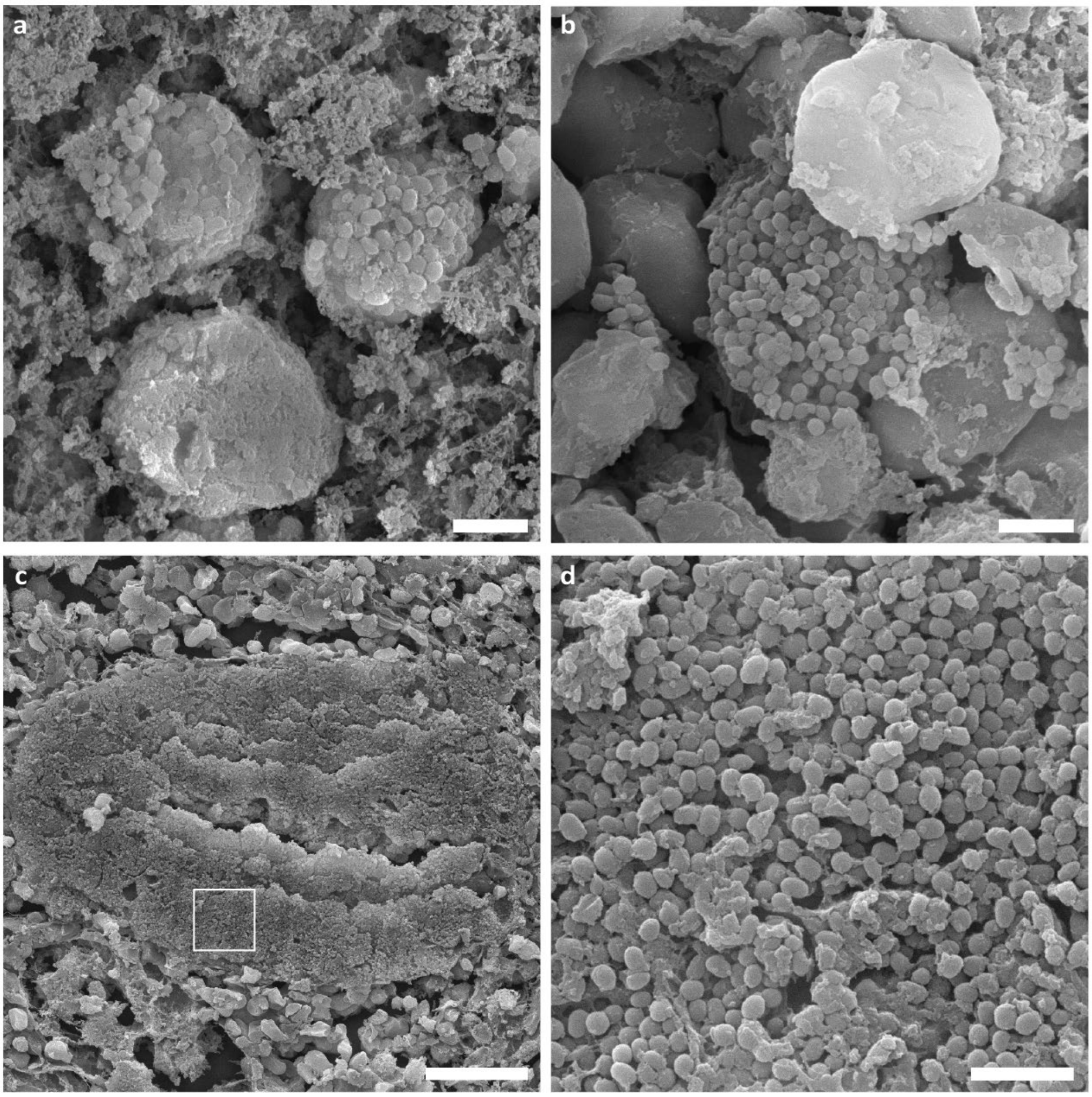

Supplementary Fig. 3 Scanning electron microscopy of melanosome clusters within the melanomacrophage centres of the kidney in Atlantic mackerel (*Scomber scombrus,* Actinopterygii). Images are of uncoverslipped histological sections. **a–c** Small **(a)**, medium **(b)** and large **(c)** melanosome clusters. **d** Detail of region indicated in **c**. Scale bars: **a, b, d** 2 μm. **c** 20 μm.

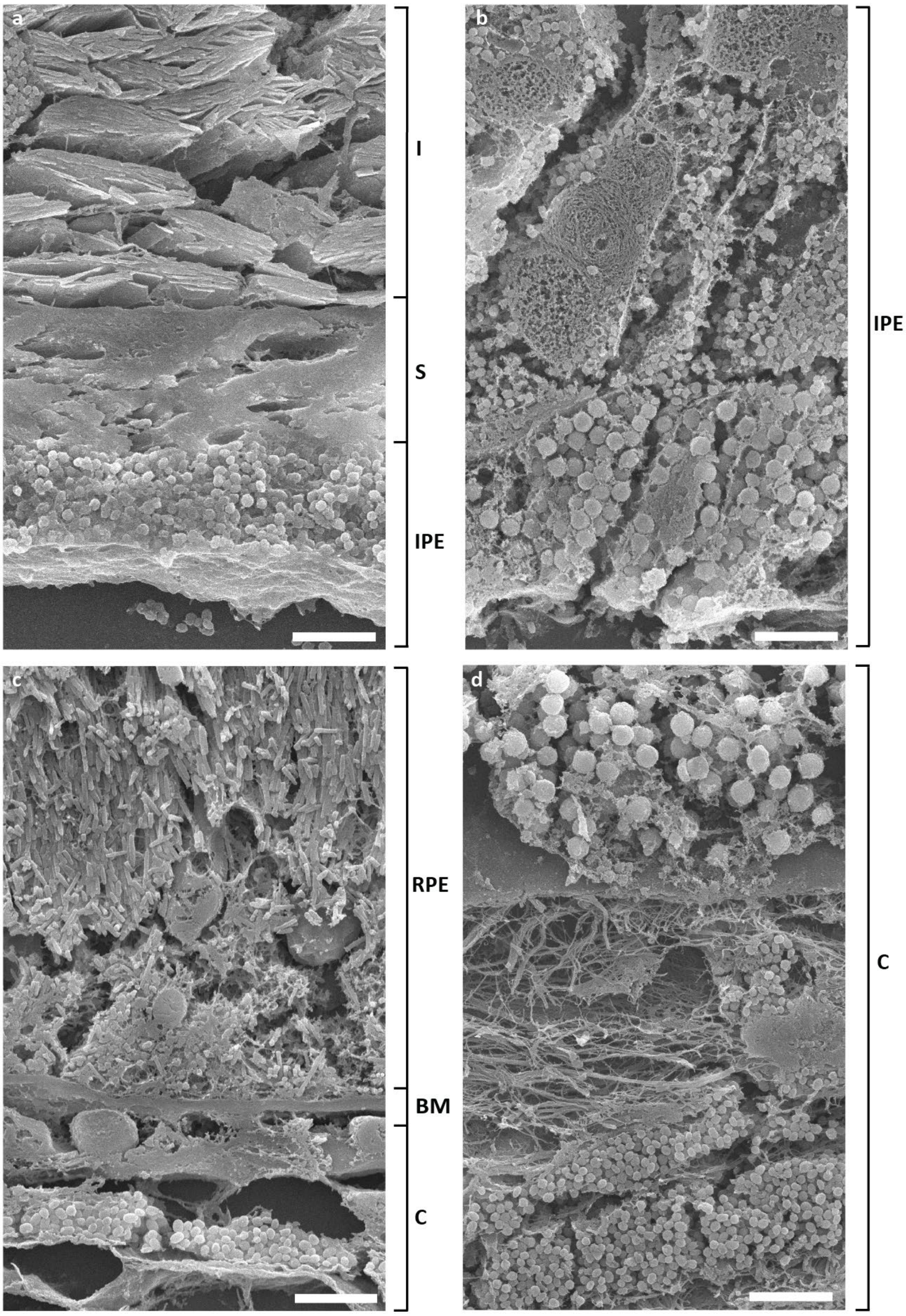

Supplementary Fig. 4 Scanning electron microscopy images of histological sections of the eye in seabass (Dicentrarchus labrax, Actinopterygii) and cuckoo skate (Leucoraja naevus, Chondrichthyes). **a** Seabass iris. Small spheroidal melanosomes occur in the iris pigment epithelium. **b** Cuckoo skate iris pigment epithelium. Small spheroidal melanosomes occur on the scleral (outer) side, while larger spheroidal melanosomes occur on the vitreal (inner) side. **c** Seabass choroid and basal retinal pigment epithelium (RPE). Small spheroidal melanosomes occur in the choroid, with cylindrical melanosomes in the RPE. **d** Cuckoo skate choroid. Small spheroidal melanosomes occur on the scleral (outer) side, whereas larger spheroidal melanosomes occur on the vitreal (inner) side. BM, Bruch's membrane; C, choroid; I, iridophore layer; IPE, iris pigment epithelium; RPE, retinal pigment epithelium; S, stroma. Scale bars: 5 µm.

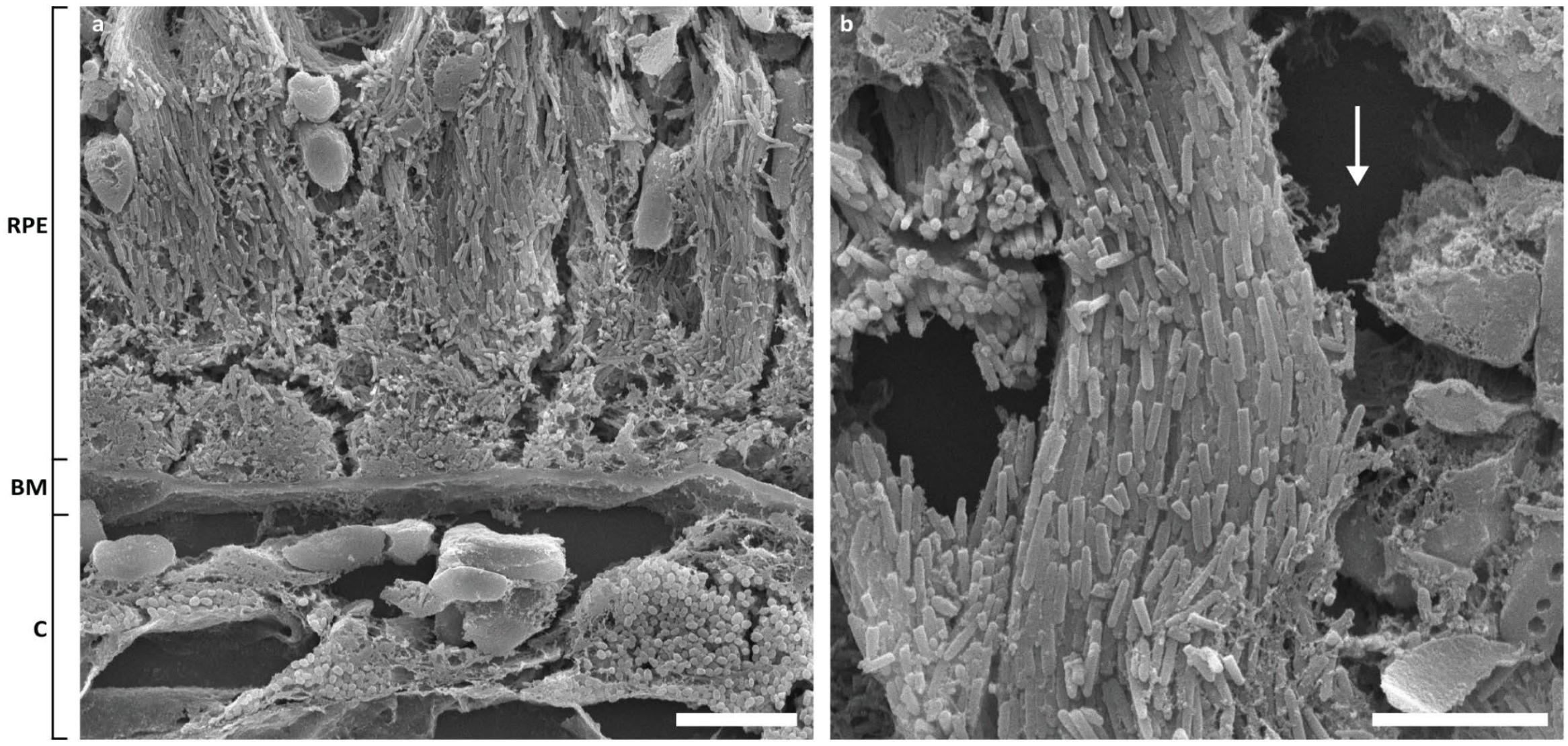

Supplementary Fig. 5 Scanning electron microscopy of melanosomes in the retinal pigment epithelium (RPE) and choroid of seabass (*Dicentrarchus labrax,* Actinopterygii). a Choroid and basal RPE. Melanosomes are spheroidal in the choroid and cylindrical in the RPE. **b** Melanosomes in the basal RPE channelling into an apical process, aligned parallel to the direction of incoming light (arrow). **BM,** Bruch's membrane. **C,** choroid. **RPE,** retinal pigment epithelium. Scale bar: **a** 10 μm. **b** 5 μm.

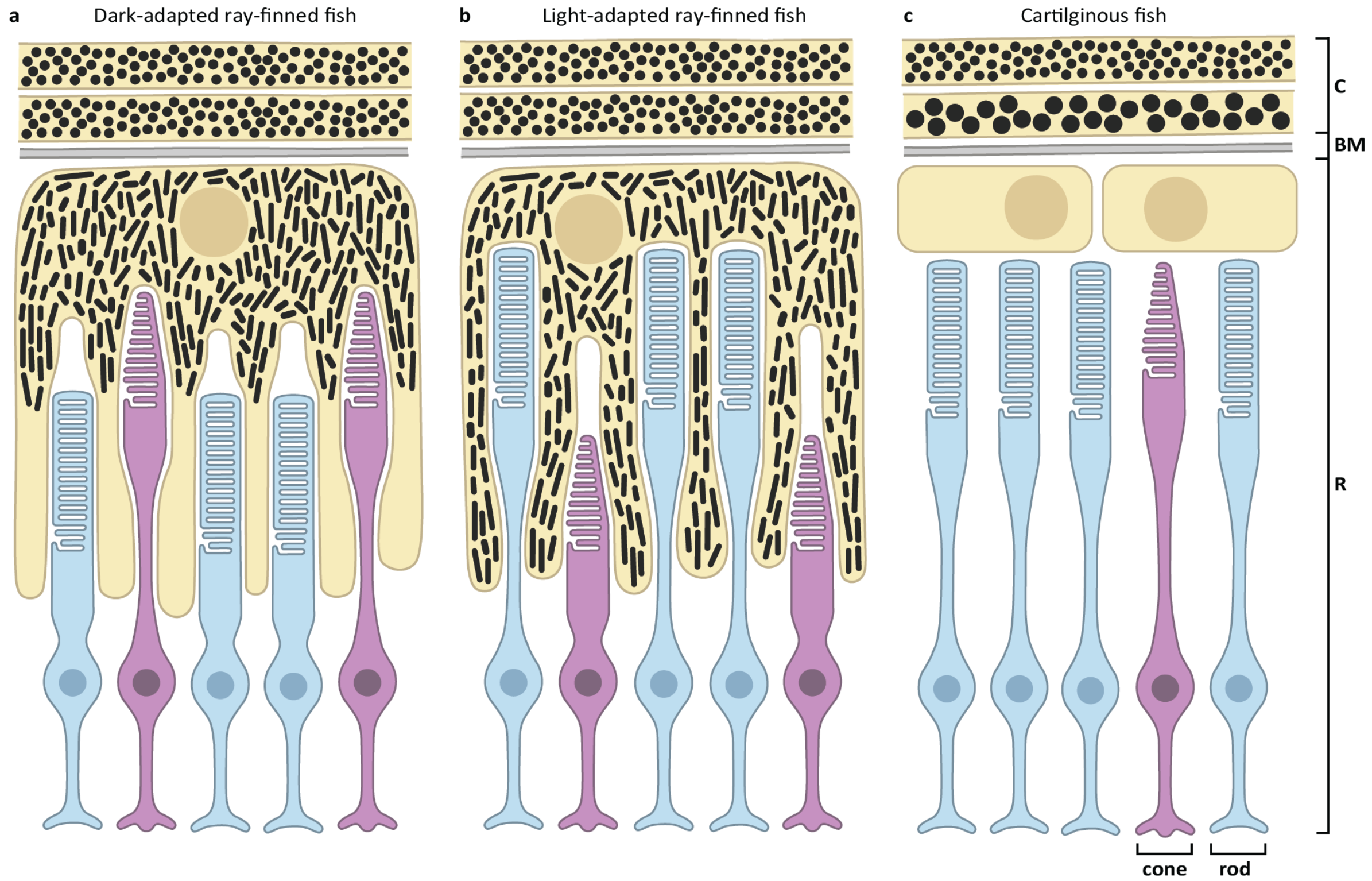

Supplementary Fig. 6 Simplified schematic illustrating the retina of ray-finned (Actinopterygii) and cartilaginous fish (Chondrichthyes). Retinomotor movements (RMM) in ray-finned fish are illustrated in **a** and **b** but are absent in cartilaginous fish **(c). a** In the dark-adapted state, cylindrical melanosomes aggregate in the cell body of retinal pigment epitheium (RPE) cells, cone (purple) myoids extend to push cone outer segments into the RPE and rod (blue) myoids retract to pull rod outer segments away from the RPE. **b** In the light-adapted state, melanosomes in the RPE migrate apically, cone-myoids retract, and rod myoids extend. Choroidal melanosomes are uniform in size and shape. **c** In cartilaginous fish, rod photoreceptors dominate but cones are present. Retinal melanosomes are scarce/absent. Choroidal melanosomes exhibit size-specific layering. BM, Bruch's membrane; C, choroid; R, retina. Illustrations are based on retinal regions in which the tapetum lucidum is absent.

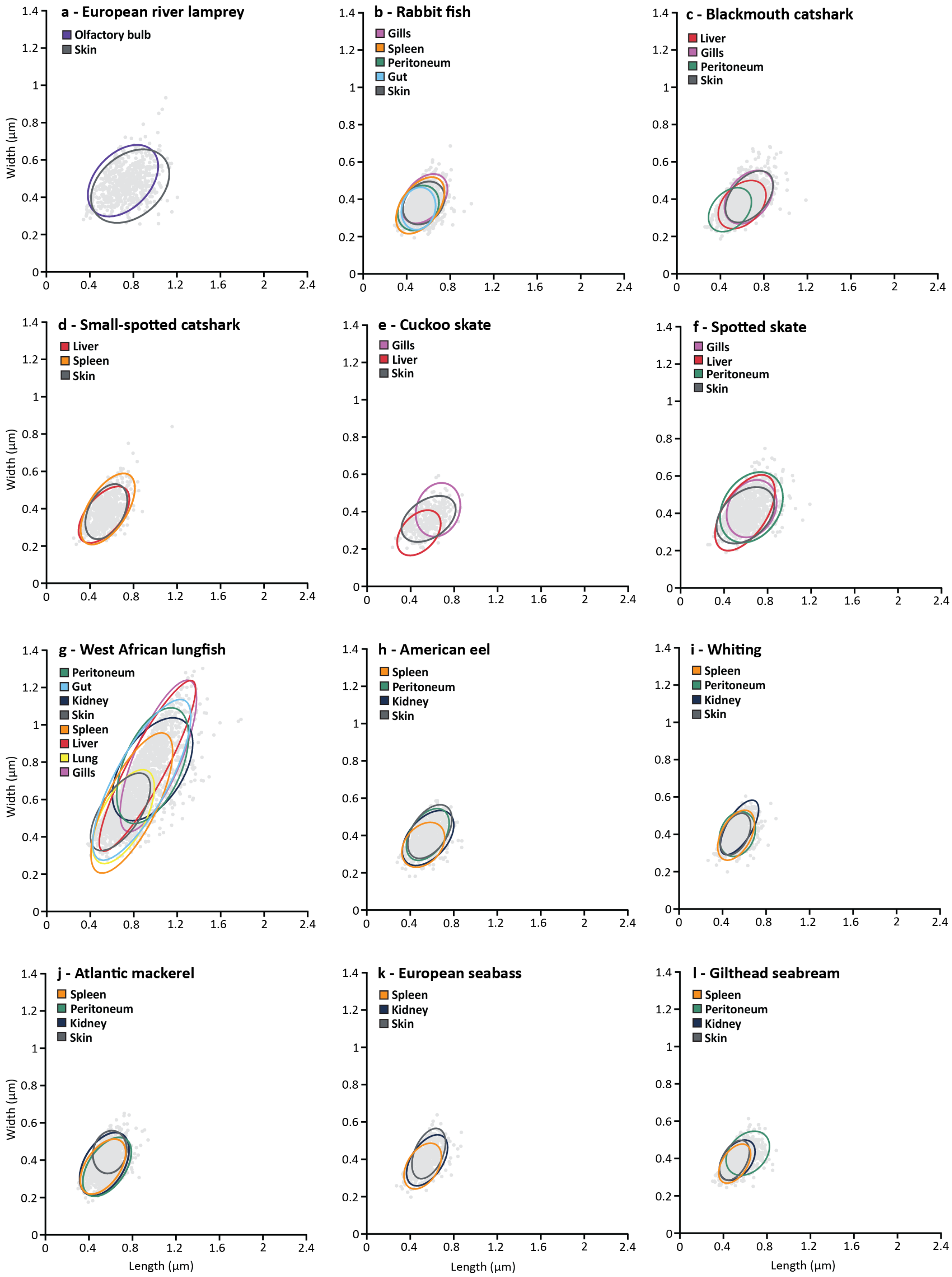

Supplementary Fig. 7 Geometry of melanosomes in tissues of extant fish. a Agnatha. **b–f** Chondrichthyes. **g** Sarcopterygii. **h–l** Actinopterygii. 95% confidence ellipses are based on measurements for 300 melanosomes where possible (n = 100 melanosomes per each of three replicates per species). For tissue yielding less than 300 observable melanosomes, all visible melanosomes were measured (Supplementary Table 6). Measurements were taken from melanosomes isolated from tissues using enzymatic extraction. See Supplementary Fig. 1 for scatterplots of raw data.

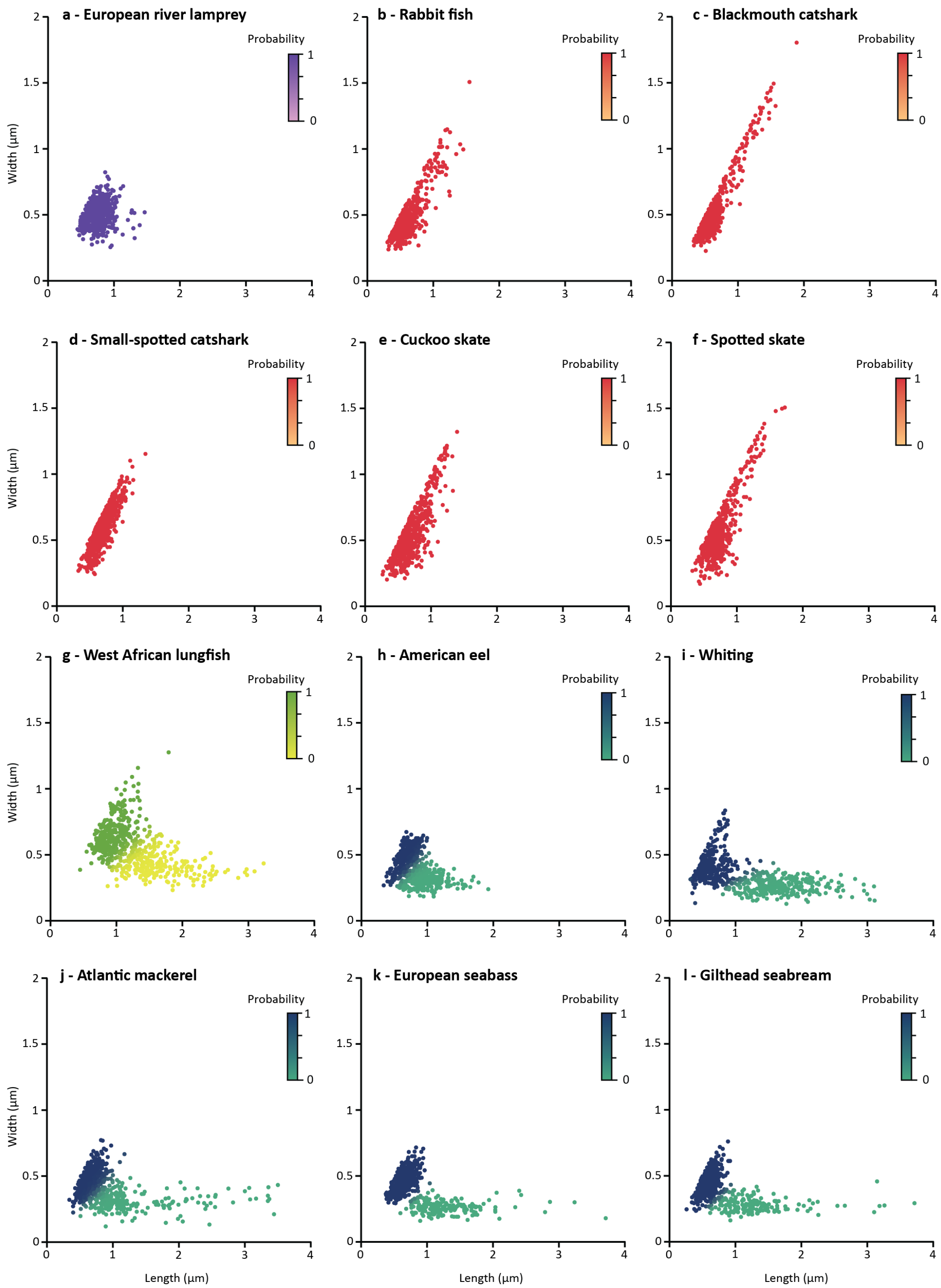

Supplementary Fig. 8 Gaussian mixture modelling of the geometry of melanosomes in the eyes of extant fish. a Agnatha. **b–f** Chondrichthyes. **g** Sarcopterygii. **h–l** Actinopterygii. Gaussian mixture models of aspect ratio, applied to scatterplots of melanosome length versus width. Datapoint colour indicates the soft clustering posterior probability of assignment to population 1 (high aspect ratio melanosomes), on a scale of 0–1. Higher values reflecting stronger population membership. Measurements were taken from melanosomes isolated from tissues using enzymatic extraction.

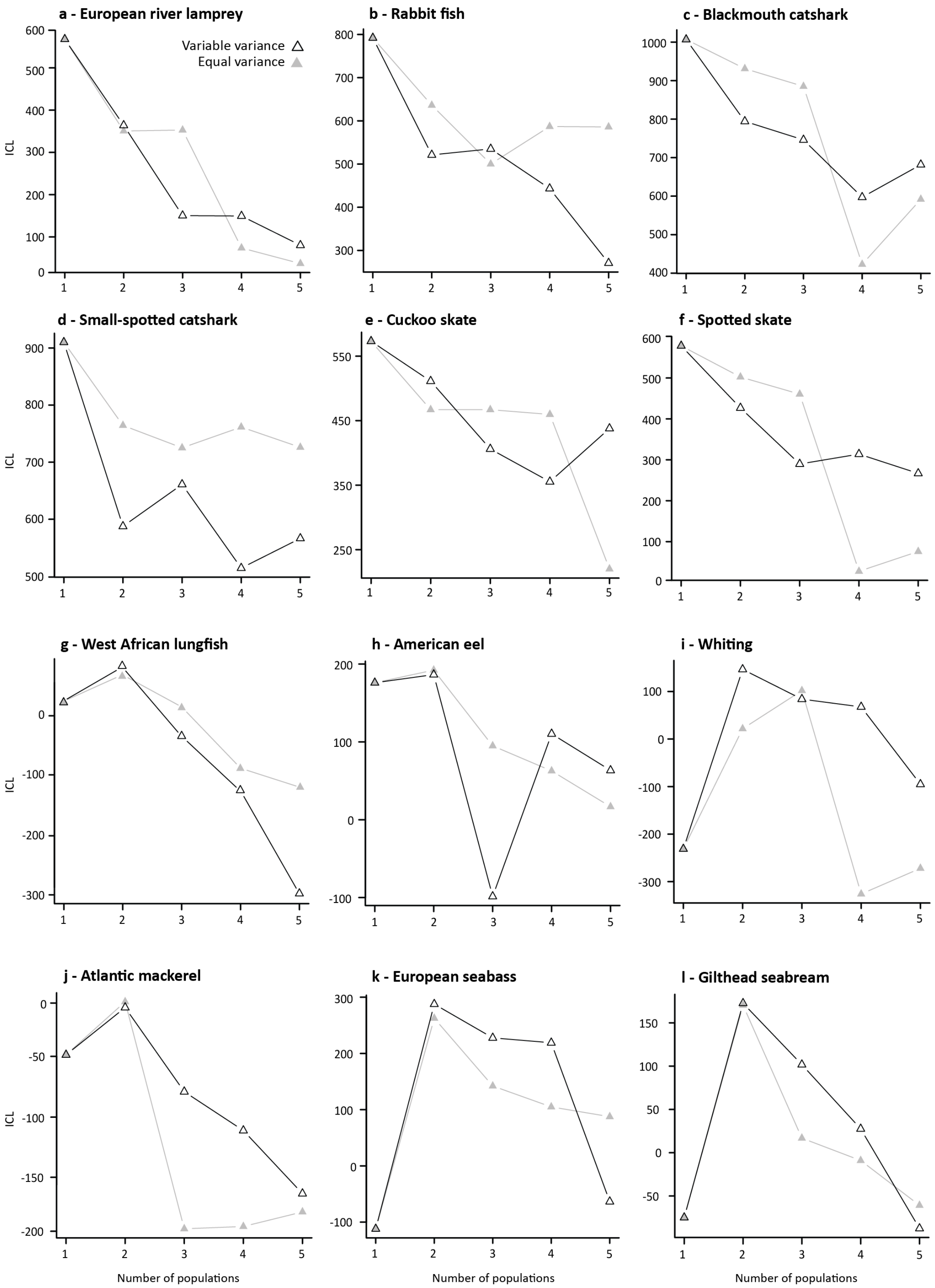

Supplementary Fig. 9 Gaussian mixture modelling and integrated completed likelihood-based model selection of population number for ocular melanosome aspect ratio from extant fish. a Agnatha. **b–f** Chondrichthyes. **g** Sarcopterygii. **h–l** Actinopterygii. The number of populations in the data for each species was determined using the ICL values returned by each model. The model with the highest ICL value was chosen and indicates the most strongly supported number of populations for a given sample. Open triangles denote the variable variance model. Filled triangles denote the equal variance model.

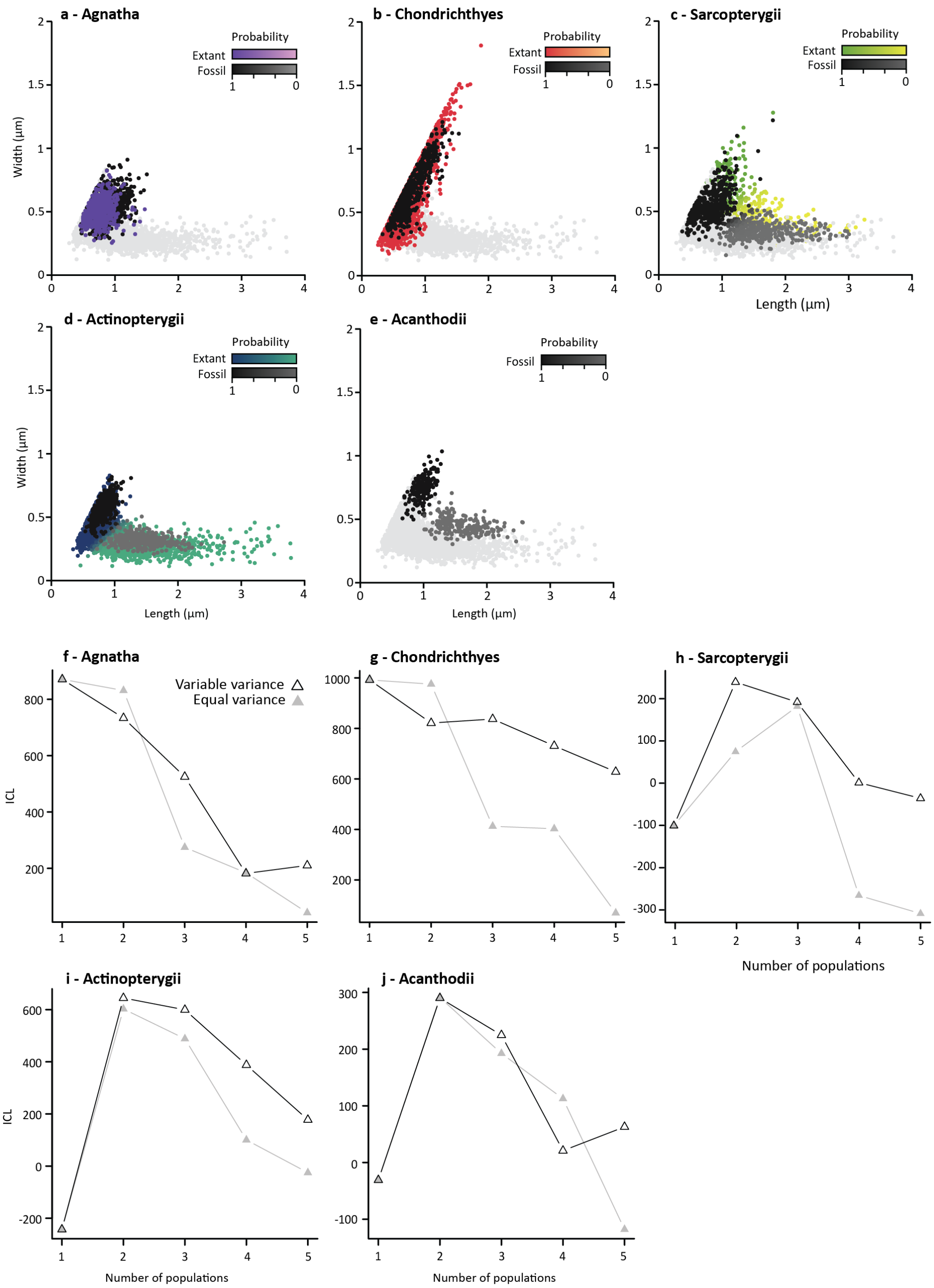

Supplementary Fig. 10 Gaussian mixture modelling and integrated completed likelihood–based model selection of the geometry of melanosomes in the eyes of fossil and extant fish. a–e Gaussian mixture models of aspect ratio, applied to scatterplots of melanosome length versus width. Datapoint colour indicates the soft clustering posterior probability for assignment to population 1 (high aspect ratio melanosomes), on a scale of 0–1. Higher values reflecting stronger population membership. Data are pooled for each major clade. Data for extant Actinopterygii are shown in light grey behind each plot for comparison. **f–j** the number of populations in the data for each species was determined using the ICL values returned by each model. The model with the highest ICL value was chosen and indicates the most strongly supported number of populations. Open triangles denote the variable variance model. Filled triangles denote the equal variance model.

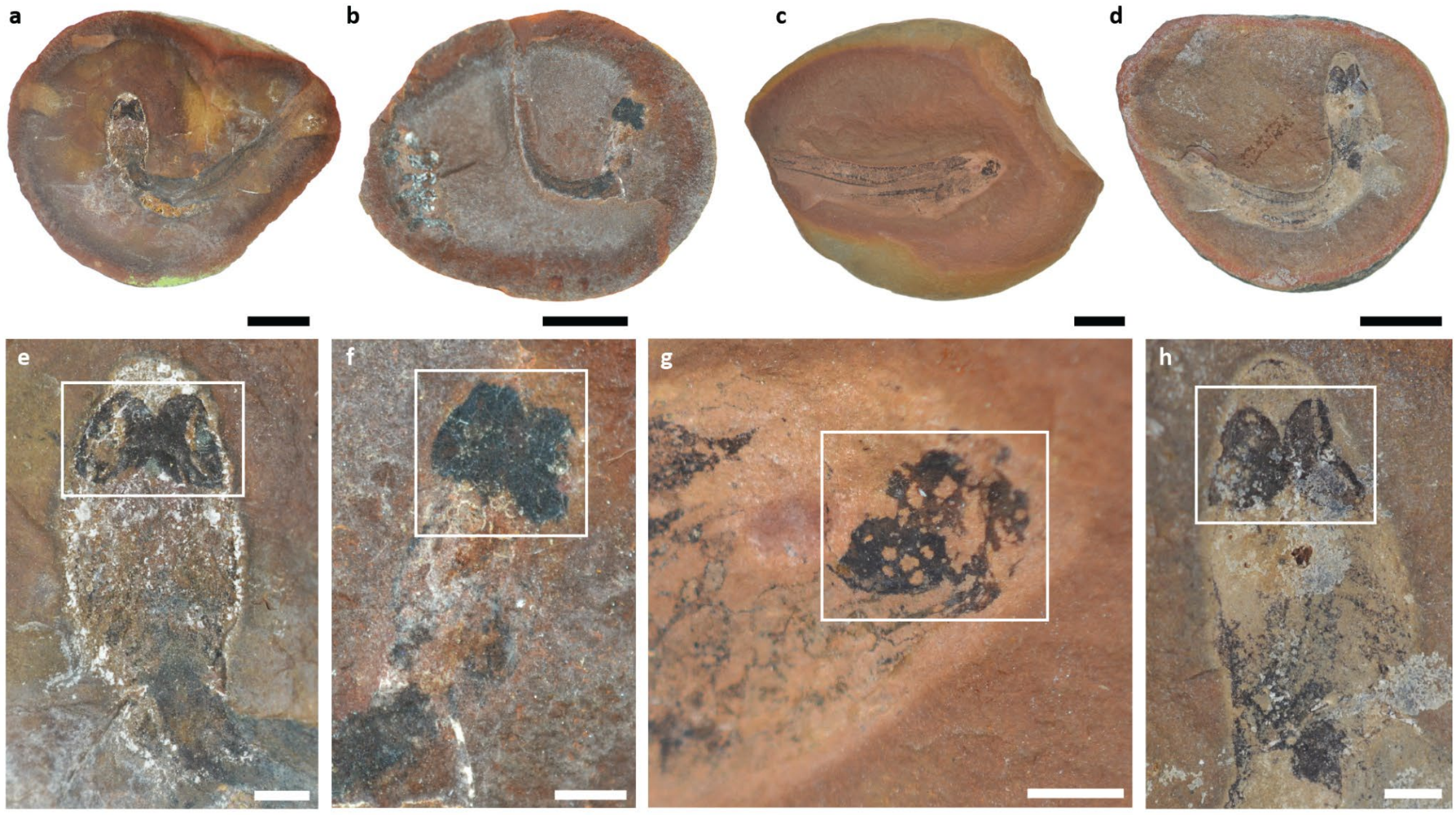

Supplementary Fig. 11 Fossil Acanthodii specimens from the Mazon Creek biota (Carboniferous, USA). All specimens are Indet. Acanthodii. **a–d** Overview of specimens. **e–h** Detail of anterior body region. **a, e** PF11509. **b, f** PF16263. **c, g** PF16201. **d, h** PF5754. White boxes show eye regions imaged with SEM. Specimen loaned from the Field Museum of Natural History, Illinois, USA. Scale bars: **a–c** 10 mm. **d–f** 2 mm.

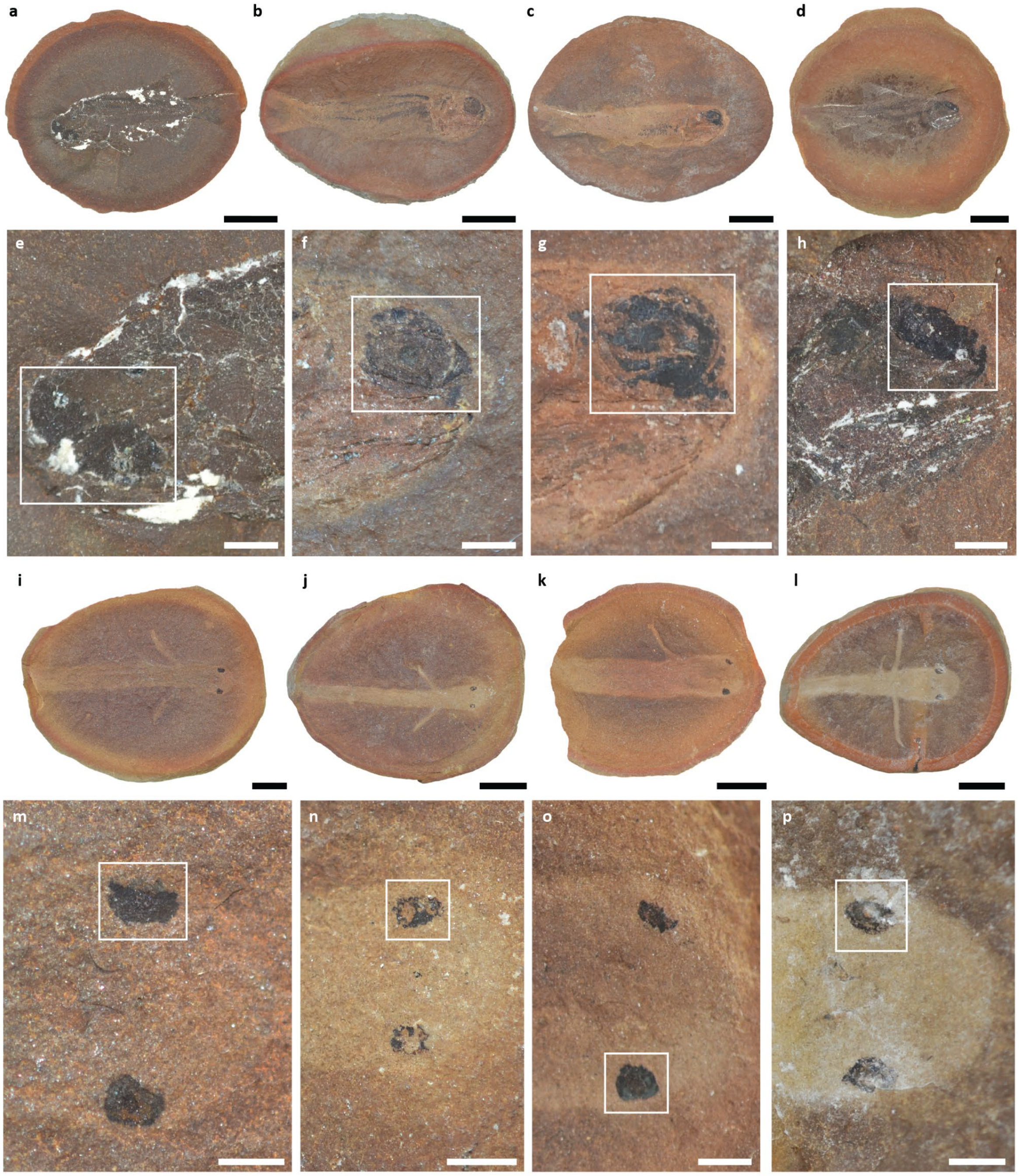

Supplementary Fig. 12 Fossil Actinopterygii and Sarcopterygii specimens from the Mazon Creek biota (Carboniferous, USA). a–h Actinopterygii. **a, e** *Elonichthys* sp. (PF16084), **b, f** *Elonichthys peltigerus* (PF7499), **c, g** *Elonichthys peltigerus (*PF7487). **d, h** *Elonichthys* sp., (PF16083). **i–p,** Sarcopterygii (*Esconicthys apopyris)*. **i, m** (PF9868). **j, n** (PF16850). **k, o** (PF9740). **l, p** *(*PF8719). White boxes show eye regions imaged with SEM. Specimen loaned from the Field Museum of Natural History, Illinois, USA. Scale bars: **a–f** 10 mm. **g–l** 2 mm.

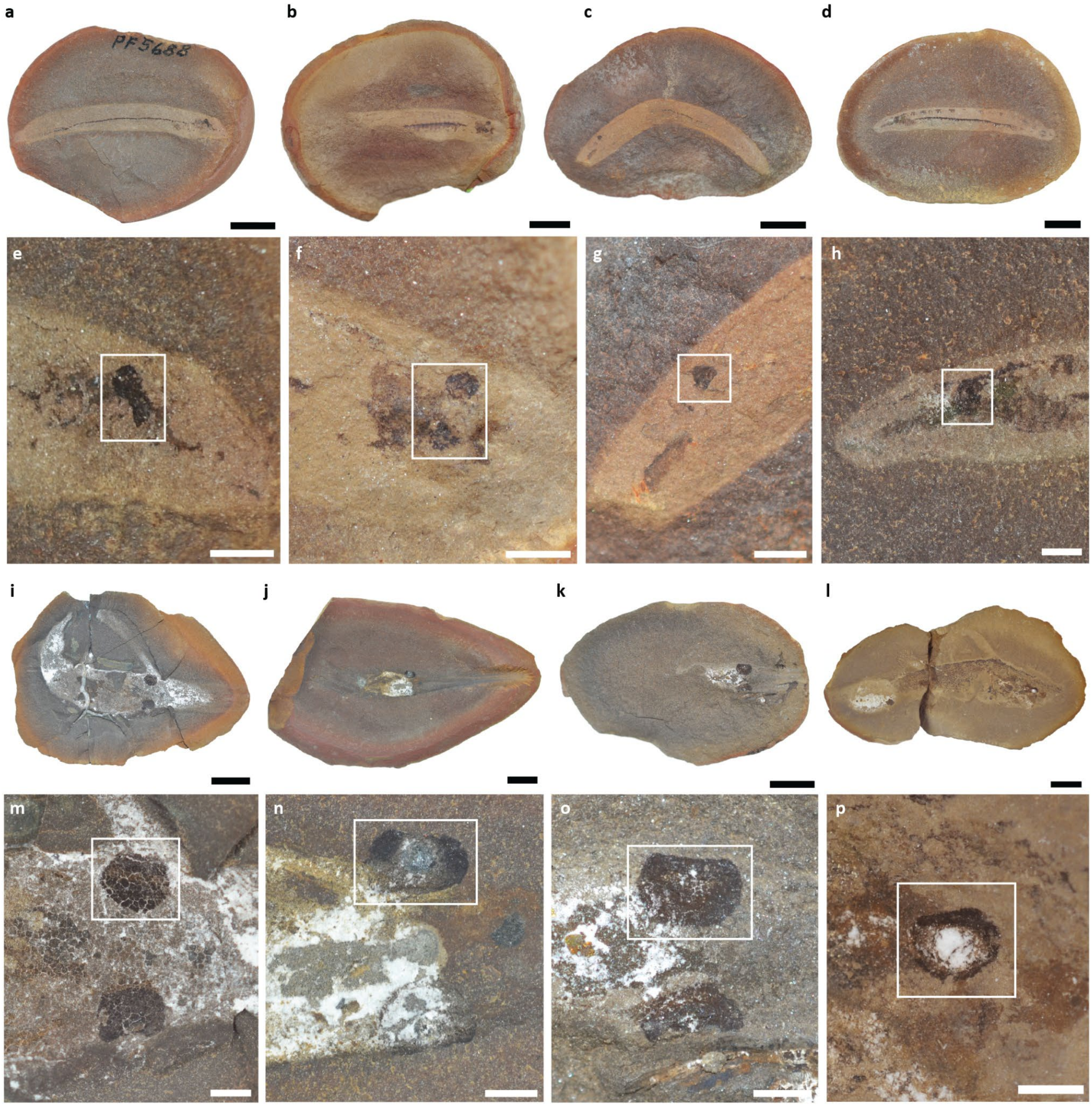

Supplementary Fig. 13 Fossil Agnatha and Chondrichthyes specimens from the Mazon Creek biota (Carboniferous, USA). a–h Agnatha. **a, e** *Mayomyzon pieckoensis* (PF5688), **b, f** *Mayomyzon pieckoensis* (PF10788), **c, g** *Gilpichthys greenei (*PF8448) **d, h** *Mayomyzon pieckoensis (*PF17992). **i–p** Chondrichthyes. **i, m** *Bandringa rayi* (PF15382). **j, n** *Bandringa* sp. (PF16367). **k, o** *Bandringa rayi (*PF3197). **l, p** *Bandringa rayi* (PF10265). White boxes show eye regions imaged with SEM. Specimen loaned from the Field Museum of Natural History, Illinois, USA. Scale bars: **a–f** 10 mm. **g–l** 2 mm.

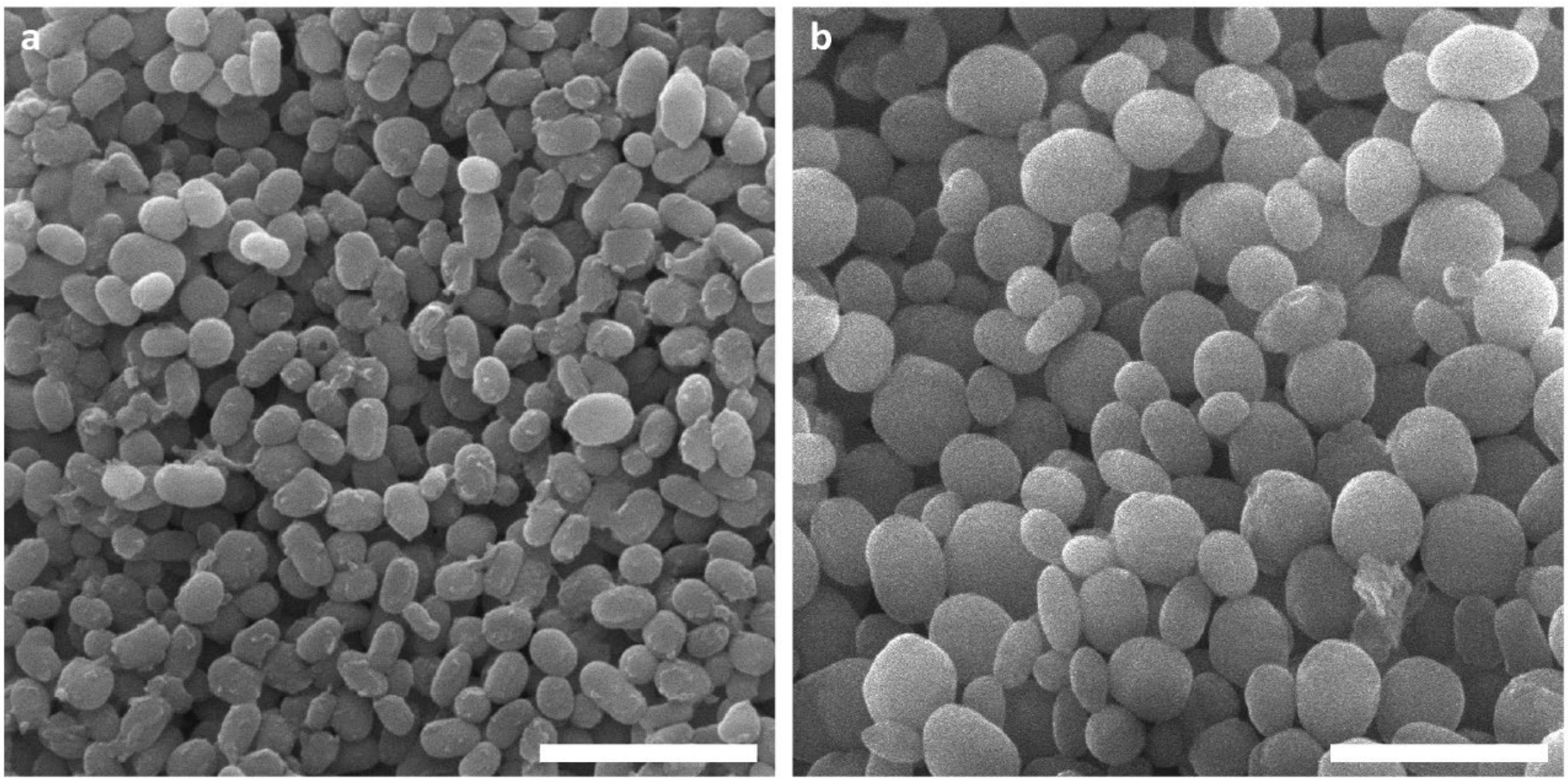

Supplementary Fig. 14 Scanning electron microscopy of melanosomes in the spleen of extant fish. a Atlantic mackerel (*Scomber scombrus,* Actinopterygii). **b** African lungfish (*Protopterus annectens,* Sarcopterygi*i*). Scale bars: 2 μm.

Supplementary Tables

Supplementary Table 1 - Details of extant fish studied. All biological replicates sampled were sexually mature.

Taxonomic group	Order	Scientific name	Common name	Biological replicate	Total length (cm)	Weight (g)
Agnatha (jawless fish)	Petromyzontiformes	*Lampetra fluviatilis*	European river lamprey	1	34	82
				2	31.5	64
				3	32.5	67
Chondrichthyes (cartilaginous fish)	Chimaerifomes	*Chimaera Monstrosa*	Rabbit fish	1	86.5	807
				2	81	539
				3	80	632
	Carcharhiniformes	*Scyliorhinus canicula*	Small-spotted catshark	1	54	582
				2	57	690
				3	57	664
		Galeus melastomus	Blackmouth catshark	1	59	554
				2	58.5	558
				3	59	573
	Rajiformes	*Raja montagui*	Spotted skate	1	59	1436
				2	60.3	1829
				3	57	1436
		Leucoraja naevus	Cuckoo skate	1	67	1763
				2	68.5	1830
				3	56	1056
Sarcopterygii (lobe-finned fish)	Ceratodontiformes	*Protopterus annectens*	West African lungfish	1	37.5	279
				2	32.3	142
				3	56	495
Actinopterygii (ray-finned fish)	Anguilliformes	*Anguilla Rostrata*	American eel	1	61	599
				2	64	694
				3	72	853
	Gadiformes	*Merlangius merlangus*	Whiting	1	38	336
				2	35.5	351
				3	35.5	345
	Scombriformes	*Scomber scombrus*	Atlantic mackerel	1	34.5	350
				2	32	275
				3	38.5	452
	Perciformes	*Dicentrarchus labrax*	European seabass	1	36.5	562
				2	34.5	464
				3	34.5	498
	Spariformes	*Sparus aurata*	Gilthead seabream	1	33	568
				2	32.5	623
				3	33	630

Supplementary Table 2 - Anatomical distribution of melanosomes in extant fish tissues. Data are based on melanosome yield from enzymatic tissue digestion.

Taxonomic group	Scientific name	Common name	Brain	Gonads	Heart	Gills	Gut	Liver	Spleen	Kidney	Peritoneum	Skin	Eye	Lung	Olfactory sac
Agnatha	*Lampetra fluviatilis*	European river lamprey	None	None	None	None	None	None	Not sampled	None	None	3 replicates	3 replicates	Not sampled	3 replicates
Chondrichthyes	*Chimaera Monstrosa*	Rabbit fish	None	None	None	3 replicates	3 replicates	None	3 replicates	None	3 replicates	3 replicates	3 replicates	Not sampled	Not sampled
	Scyliorhinus canicula	Small-spotted catshark	None	None	None	None	None	3 replicates	3 replicates	None	None	3 replicates	3 replicates	Not sampled	Not sampled
	Galeus melastomus	Blackmouth catshark	None	None	None	3 replicates	None	3 replicates	None	None	3 replicates	3 replicates	3 replicates	Not sampled	Not sampled
	Raja montagui	Spotted skate	None	None	None	1 replicate	None	3 replicates	None	None	3 replicates	3 replicates	3 replicates	Not sampled	Not sampled
	Leucoraja naevus	Cuckoo skate	None	None	None	1 replicate	None	1 replicate	None	None	None	3 replicates	3 replicates	Not sampled	Not sampled
Sarcopterygii	*Protopterus annectens*	West African lungfish	None	None	None	2 replicates	3 replicates	2 replicates	3 replicates	3 replicates	3 replicates	3 replicates	3 replicates	3 replicates	Not sampled
Actinopterygii	*Anguilla Rostrata*	American eel	None	None	None	None	None	None	2 replicates	3 replicates	3 replicates	3 replicates	3 replicates	Not sampled	Not sampled
	Merlangius merlangus	Whiting	None	None	None	None	None	None	3 replicates	3 replicates	3 replicates	3 replicates	3 replicates	Not sampled	Not sampled
	Scomber scombrus	Atlantic mackerel	None	None	None	None	None	None	3 replicates	3 replicates	3 replicates	3 replicates	3 replicates	Not sampled	Not sampled
	Dicentrarchus labrax	European seabass	None	None	None	None	None	None	3 replicates	3 replicates	None	3 replicates	3 replicates	Not sampled	Not sampled
	Sparus aurata	Gilthead seabream	None	None	None	None	None	None	3 replicates	3 replicates	3 replicates	3 replicates	3 replicates	Not sampled	Not sampled

Key
Melanosomes in 1 replicate
Melanosomes in 2 replicates
Melanosomes in 3 replicates
No melanosomes in extract
Not sampled

Supplementary Table 3 - Details of fossil specimens studied. All specimens are from the Mazon Creek Konservat-Lagerstätte and are housed in the collections of the Field Museum of Natural History, Illinois, USA.

Taxonomic group	Classification	Specimen ID	Melanosomes imaged
Agnatha	*Mayomyzon pieckoensis*	PF17992	Yes
Agnatha	*Mayomyzon pieckoensis*	PF5688	Yes
Agnatha	*Mayomyzon pieckoensis*	PF10788	Yes
Agnatha	*Gilpichthyes greenei*	PF8448	Yes
Acanthodii	Indet.	PF16201	Yes
Acanthodii	Indet.	PF5754	Yes
Acanthodii	Indet.	PF16263	No
Acanthodii	Indet.	PF11509	No
Sarcopterygii	*Esconichthys apopyris*	PF8719	Yes
Sarcopterygii	*Esconichthys apopyris*	PF9740	Yes
Sarcopterygii	*Esconichthys apopyris*	PF9868	Yes
Sarcopterygii	*Esconichthys apopyris*	PF16850	Yes
Chondrichthyes	*Bandringa rayi*	PF10265	Yes
Chondrichthyes	*Bandringa rayi*	PF15382	No
Chondrichthyes	*Bandringa* sp.	PF16367	Yes
Chondrichthyes	*Bandringa rayi*	PF3197	Yes
Actinopterygii	*Elonichthys* sp.	PF16084	Yes
Actinopterygii	*Elonichthys* sp.	PF16083	Yes
Actinopterygii	*Elonichthys peltigerus*	PF7487	Yes
Actinopterygii	*Elonichthys peltigerus*	PF7499	Yes

Supplementary Table 4 - Shapiro-Wilk and two-sided one-way ANOVA test results for melanosome length, width and aspect ratio among tissues in each extant taxon studied. ANOVAs were performed using linear models, with tissue as a fixed effect. Filled cells represent *p* values < 0.05 (statistically significant). F, F-statistic; df1, numerator degrees of freedom; df2, denominator degrees of freedom; n, number of melanosomes measured.

Taxonomic group	Scientific name	Common name	Tissue	Shapiro-Wilk			ANOVA											
				Length	Width	Aspect Ratio	Length				Width				Aspect ratio			
							F	df_1	df_2	*p*	F	df_1	df_2	*p*	F	df_1	df_2	*p*
Agnatha	*Lampetra fluviatilis*	European river lamprey	Skin<br>Eye<br>Olfactory sac	0.81	0.79	0.90	2.13	2	6	0.20	1.34	2	6	0.33	4.28	2	6	0.07
Chondrichthyes	*Chimaera monstrosa*	Rabbit fish	Gills<br>Spleen<br>Peritoneum<br>Skin<br>Gut<br>Eye	0.56	1.00	0.21	5.20	5	12	9.04E-03	8.05	5	12	1.55E-03	3.51	5	12	0.03
	Scyliorhinus canicula	Small-spotted catshark	Liver<br>Spleen<br>Skin<br>Eye	0.67	0.42	0.44	13.42	3	8	1.73E-03	30.95	3	8	9.43E-05	13.37	3	8	1.75E-03
	Galeus melastomus	Blackmouth catshark	Gills<br>Liver<br>Peritoneum<br>Skin<br>Eye	0.98	0.59	0.03	25.49	4	10	3.15E-05	58.35	4	10	6.78E-07	51.79	4	10	1.19E-06
	Raja montagui	Spotted skate	Gills<br>Liver<br>Peritoneum<br>Skin<br>Eye	0.29	0.23	0.35	5.41	4	8	0.02	12.65	4	8	1.55E-03	3.04	4	8	0.08
	Leucoraja naevus	Cuckoo skate	Gills<br>Liver<br>Skin<br>Eye	0.08	0.29	0.32	10.40	3	4	0.02	14.91	3	4	0.01	10.60	3	4	0.02
Sarcopterygii	*Protopterus annectens*	West African Lungfish	Gills<br>Liver<br>Spleen<br>Kidney<br>Peritoneum<br>Skin<br>Eye<br>Gut<br>Lung	0.21	0.73	0.42	20.10	8	16	5.78E-07	11.69	8	16	2.26E-05	14.27	8	16	6.09E-06
Actinopterygii	*Anguilla Rostrata*	American eel	Spleen<br>Kidney<br>Peritoneum<br>Skin<br>Eye	0.81	0.77	0.42	30.38	4	8	6.92E-05	1.88	4	8	0.21	33.97	4	8	4.56E-05
	Merlangius merlangus	Whiting	Spleen<br>Kidney<br>Peritoneum<br>Skin<br>Eye	0.48	0.62	0.21	105.65	4	10	3.88E-08	5.50	4	10	0.01	191.24	4	10	2.12E-09
	Scomber scombrus	Atlantic mackerel	Spleen<br>Kidney<br>Peritoneum<br>Skin<br>Eye	0.60	0.85	0.33	44.67	4	9	6.12E-06	2.66	4	9	0.10	19.06	4	9	2.03E-04
	Dicentrarchus labrax	European seabass	Spleen<br>Kidney<br>Skin<br>Eye	0.47	0.41	0.07	40.71	3	8	3.43E-05	4.15	3	8	0.05	18.44	3	8	5.95E-04
	Sparus aurata	Gilthead seabream	Spleen<br>Kidney<br>Peritoneum<br>Skin<br>Eye	0.52	0.22	1.00	83.59	4	10	1.21E-07	2.43	4	10	0.12	51.44	4	10	1.23E-06

Supplementary Table 5 - Tukey post hoc test results and multivariate Bhattacharyya coefficients for pairwise comparisons of melanosome geometry among tissues in each extant taxon studied. Pairwise comparisons are two-sided and *p* values were adjusted for multiple comparisons using Tukey's method. Bhattacharyya coefficients were computed from the length, width and aspect ratio data. Filled cells represent *p* values < 0.05 (statistically significant).

Taxonomic group	Scientific name	Common name	Sample (tissue)		Post hoc test			Bhattacharyya coefficient
			Group 1	Group 2	Length	Width	Aspect ratio	Length, width and aspect ratio
Agnatha	*Lampetra fluviatilis*	Eurpoean river lamprey	Eye	Nasal Sac	0.57	0.69	0.98	0.96
			Eye	Skin	0.60	0.30	0.09	0.94
			Nasal Sac	Skin	0.18	0.72	0.11	0.93
Chondrichthyes	*Chimaera monstrosa*	Rabbit fish	Eye	Gills	0.79	0.15	0.17	0.75
			Eye	Gut	0.02	1.99E-03	0.05	0.56
			Eye	Peritoneum	0.02	2.16E-03	0.08	0.62
			Eye	Skin	0.32	0.02	0.03	0.62
			Eye	Spleen	0.07	9.21E-03	0.13	0.75
			Gills	Gut	0.13	0.16	0.96	0.80
			Gills	Peritoneum	0.14	0.18	0.99	0.85
			Gills	Skin	0.94	0.81	0.89	0.91
			Gills	Spleen	0.44	0.56	1.00	0.93
			Gut	Peritoneum	1.00	1.00	1.00	0.98
			Gut	Skin	0.46	0.73	1.00	0.93
			Gut	Spleen	0.95	0.93	0.99	0.91
			Peritoneum	Skin	0.47	0.76	0.99	0.93
			Peritoneum	Spleen	0.96	0.94	1.00	0.95
			Skin	Spleen	0.90	1.00	0.94	0.94
	Scyliorhinus canicula	Small-spotted catshark	Eye	Liver	2.02E-03	1.36E-04	3.04E-03	0.51
			Eye	Skin	4.05E-03	2.34E-04	3.56E-03	0.54
			Eye	Spleen	0.01	4.71E-04	5.17E-03	0.66
			Liver	Skin	0.93	0.92	1.00	0.97
			Liver	Spleen	0.58	0.56	0.97	0.95
			Skin	Spleen	0.88	0.88	0.99	0.94
	Galeus melastomus	Blackmouth catshark	Eye	Gills	0.74	3.28E-05	3.28E-06	0.34
			Eye	Liver	0.35	2.23E-06	3.26E-06	0.33
			Eye	Peritoneum	2.10E-04	4.73E-07	3.73E-04	0.42
			Eye	Skin	0.65	3.81E-05	2.98E-06	0.31
			Gills	Liver	0.06	0.07	1.00	0.89
			Gills	Peritoneum	5.48E-05	2.83E-03	5.18E-03	0.45
			Gills	Skin	1.00	1.00	1.00	0.98
			Liver	Peritoneum	2.53E-03	0.27	5.13E-03	0.67
			Liver	Skin	0.05	0.06	1.00	0.89
			Peritoneum	Skin	4.61E-05	2.30E-03	4.37E-03	0.45
	Raja montagui	Spotted skate	Eye	Gills	0.49	0.04	0.23	0.60
			Eye	Liver	0.03	3.53E-03	0.32	0.70
			Eye	Peritoneum	0.23	7.14E-03	0.11	0.67
			Eye	Skin	0.02	1.37E-03	0.11	0.59
			Gills	Liver	0.81	1.00	0.90	0.88
			Gills	Peritoneum	1.00	1.00	1.00	0.97
			Gills	Skin	0.75	0.91	1.00	0.89
			Liver	Peritoneum	0.54	0.97	0.92	0.92
			Liver	Skin	1.00	0.90	0.91	0.96
			Peritoneum	Skin	0.44	0.61	1.00	0.90
	Leucoraja naevus	Cuckoo skate	Eye	Gills	0.68	0.15	0.07	0.52
			Eye	Liver	0.03	0.02	0.04	0.28
			Eye	Skin	0.04	0.02	0.04	0.52
			Gills	Liver	0.13	0.28	0.93	0.28
			Gills	Skin	0.37	0.69	0.96	0.70
			Liver	Skin	0.40	0.52	0.66	0.71

Supplementary Table 5 (continued)

Taxonomic group	Scientific name	Common name	Sample (tissue)		Post hoc test			Bhattacharyya coefficient
			Group 1	Group 2	Length	Width	Aspect ratio	Length, width and aspect ratio
Sarcopterygii	*Protopterus annectens*	West African Lungfish	Eye	Gills	0.08	1.19E-03	3.77E-05	0.28
			Eye	Gut	1.35E-04	0.12	1.53E-05	0.31
			Eye	Kidney	2.57E-03	0.02	2.60E-05	0.31
			Eye	Liver	1.65E-03	0.02	1.50E-05	0.20
			Eye	Lung	9.41E-07	1.00	2.55E-04	0.20
			Eye	Peritoneum	2.15E-03	5.59E-03	8.64E-06	0.28
			Eye	Skin	4.90E-07	1.00	1.74E-05	0.18
			Eye	Spleen	8.86E-06	0.99	1.27E-04	0.28
			Gills	Gut	0.23	0.20	1.00	0.80
			Gills	Kidney	0.93	0.69	1.00	0.88
			Gills	Liver	0.66	0.92	1.00	0.76
			Gills	Lung	1.19E-03	3.70E-04	0.70	0.34
			Gills	Peritoneum	0.91	0.92	1.00	0.89
			Gills	Skin	5.36E-04	6.42E-04	1.00	0.31
			Gills	Spleen	0.02	5.25E-03	0.87	0.60
			Gut	Kidney	0.79	0.97	1.00	0.87
			Gut	Liver	1.00	0.90	0.99	0.88
			Gut	Lung	0.10	0.03	0.76	0.65
			Gut	Peritoneum	0.84	0.78	1.00	0.87
			Gut	Skin	0.04	0.06	1.00	0.64
			Gut	Spleen	0.77	0.44	0.93	0.90
			Kidney	Liver	1.00	1.00	0.96	0.77
			Kidney	Lung	4.90E-03	3.99E-03	0.91	0.41
			Kidney	Peritoneum	1.00	1.00	1.00	0.98
			Kidney	Skin	1.96E-03	7.64E-03	1.00	0.40
			Kidney	Spleen	0.09	0.08	0.99	0.68
			Liver	Lung	0.06	5.31E-03	0.38	0.59
			Liver	Peritoneum	1.00	1.00	1.00	0.83
			Liver	Skin	0.03	9.51E-03	0.99	0.58
			Liver	Spleen	0.49	0.08	0.57	0.82
			Lung	Peritoneum	5.87E-03	1.45E-03	0.55	0.38
			Lung	Skin	1.00	1.00	0.80	0.95
			Lung	Spleen	0.83	0.80	1.00	0.87
			Peritoneum	Skin	2.35E-03	2.74E-03	1.00	0.37
			Peritoneum	Spleen	0.10	0.03	0.78	0.67
			Skin	Spleen	0.56	0.94	0.95	0.82
Actinopterygii	*Anguilla Rostrata*	American eel	Eye	Kidney	4.15E-04	1.00	4.09E-04	0.34
			Eye	Peritoneum	1.71E-04	0.99	6.15E-05	0.28
			Eye	Skin	2.96E-04	0.88	6.33E-05	0.35
			Eye	Spleen	1.20E-04	0.43	1.19E-03	0.26
			Kidney	Peritoneum	1.00	0.97	0.73	0.94
			Kidney	Skin	0.99	0.84	0.75	0.94
			Kidney	Spleen	0.69	0.61	0.85	0.92
			Peritoneum	Skin	0.96	0.99	1.00	0.96
			Peritoneum	Spleen	0.67	0.28	0.22	0.86
			Skin	Spleen	0.38	0.16	0.23	0.81
	Merlangius merlangus	Whiting	Eye	Kidney	1.75E-07	7.60E-03	3.72E-09	0.13
			Eye	Peritoneum	1.39E-07	0.08	1.09E-08	0.15
			Eye	Skin	1.03E-07	0.07	3.96E-09	0.11
			Eye	Spleen	1.33E-07	0.07	9.55E-09	0.16
			Kidney	Peritoneum	0.99	0.57	0.38	0.92
			Kidney	Skin	0.88	0.64	1.00	0.91
			Kidney	Spleen	0.99	0.61	0.50	0.93
			Peritoneum	Skin	0.98	1.00	0.43	0.94
			Peritoneum	Spleen	1.00	1.00	1.00	0.98
			Skin	Spleen	0.99	1.00	0.56	0.93
	Scomber scombrus	Atlantic mackerel	Eye	Kidney	1.12E-05	0.88	8.21E-04	0.23
			Eye	Peritoneum	1.89E-05	0.62	0.01	0.23
			Eye	Skin	3.74E-05	0.65	1.48E-04	0.18
			Eye	Spleen	2.21E-05	0.76	2.46E-03	0.19
			Kidney	Peritoneum	0.96	0.98	0.28	0.96
			Kidney	Skin	0.59	0.22	0.52	0.79
			Kidney	Spleen	1.00	1.00	1.00	0.97
			Peritoneum	Skin	0.92	0.11	0.03	0.73
			Peritoneum	Spleen	0.89	1.00	0.48	0.94
			Skin	Spleen	0.51	0.19	0.49	0.75
	Dicentrarchus labrax	European seabass	Eye	Kidney	1.03E-04	1.00	2.59E-03	0.22
			Eye	Skin	1.50E-04	0.27	5.88E-04	0.18
			Eye	Spleen	4.83E-05	0.47	2.80E-03	0.17
			Kidney	Skin	0.97	0.25	0.55	0.89
			Kidney	Spleen	0.77	0.49	1.00	0.94
			Skin	Spleen	0.53	0.03	0.51	0.80
	Sparus aurata	Gilthead seabream	Eye	Kidney	5.64E-07	0.96	7.51E-06	0.20
			Eye	Peritoneum	2.43E-05	0.66	2.27E-04	0.29
			Eye	Skin	2.49E-07	0.95	1.05E-06	0.18
			Eye	Spleen	2.47E-07	0.53	7.05E-06	0.17
			Kidney	Peritoneum	6.44E-03	0.32	0.04	0.74
			Kidney	Skin	0.71	1.00	0.18	0.97
			Kidney	Spleen	0.70	0.87	1.00	0.97
			Peritoneum	Skin	1.08E-03	0.30	1.19E-03	0.66
			Peritoneum	Spleen	1.07E-03	0.08	0.04	0.61
			Skin	Spleen	1.00	0.89	0.20	0.97

Supplementary Table 6 - Summary statistics for melanosome geometry for tissues in each extant taxon studied. IQR, interquartile range; SD, standard deviation; n, number of melanosomes measured.

Taxonomic group	Scientific name	Common name	Tissue	n	Length (µm)					Width (µm)					Aspect ratio				
					Mean	SD	Median	IQR	Range	Mean	SD	Median	IQR	Range	Mean	SD	Median	IQR	Range
Agnatha	*Lampetra fluviatilis*	European river lamprey	Skin	300	0.77	0.17	0.78	0.24	0.85	0.46	0.09	0.45	0.13	0.59	0.62	0.15	0.61	0.19	0.70
			Eye	600	0.75	0.15	0.74	0.18	1.03	0.51	0.09	0.52	0.13	0.57	0.71	0.15	0.71	0.22	0.75
			olfactory sac	300	0.72	0.16	0.71	0.21	0.83	0.49	0.09	0.49	0.12	0.68	0.70	0.15	0.69	0.22	0.75
Chondrichthyes	*Chimaera monstrosa*	Rabbit fish	Gills	126	0.56	0.10	0.55	0.14	0.63	0.40	0.06	0.40	0.08	0.28	0.74	0.13	0.74	0.18	0.67
			Spleen	300	0.52	0.11	0.52	0.14	0.65	0.37	0.07	0.36	0.09	0.49	0.73	0.14	0.74	0.21	0.62
			Peritoneum	300	0.50	0.09	0.49	0.12	0.52	0.36	0.06	0.35	0.08	0.34	0.73	0.14	0.74	0.22	0.62
			Skin	300	0.55	0.09	0.54	0.13	0.49	0.38	0.06	0.38	0.07	0.37	0.72	0.13	0.71	0.19	0.62
			Gut	300	0.50	0.08	0.50	0.10	0.51	0.35	0.05	0.35	0.06	0.29	0.72	0.14	0.73	0.21	0.61
			Eye	600	0.60	0.19	0.55	0.17	1.26	0.47	0.16	0.42	0.13	1.27	0.79	0.12	0.80	0.17	0.65
	Scyliorhinus canicula	Small-spotted catshark	Liver	300	0.54	0.11	0.54	0.14	0.62	0.37	0.07	0.36	0.09	0.45	0.70	0.13	0.69	0.18	0.60
			Spleen	150	0.58	0.12	0.57	0.16	0.92	0.40	0.10	0.39	0.11	0.60	0.71	0.14	0.70	0.20	0.55
			Skin	300	0.56	0.09	0.56	0.12	0.56	0.38	0.07	0.38	0.09	0.36	0.70	0.13	0.69	0.19	0.64
			Eye	600	0.71	0.15	0.70	0.20	1.03	0.58	0.15	0.58	0.20	0.91	0.82	0.11	0.82	0.15	0.59
	Galeus melastomus	Blackmouth catshark	Gills	300	0.67	0.11	0.66	0.13	0.81	0.42	0.07	0.41	0.08	0.42	0.64	0.12	0.63	0.15	0.65
			Liver	300	0.60	0.11	0.60	0.14	0.73	0.38	0.06	0.38	0.08	0.45	0.64	0.12	0.62	0.16	0.68
			Peritoneum	300	0.49	0.09	0.49	0.13	0.54	0.35	0.05	0.34	0.07	0.34	0.72	0.14	0.71	0.22	0.62
			Skin	300	0.67	0.11	0.66	0.14	0.60	0.42	0.07	0.41	0.08	0.42	0.64	0.11	0.63	0.14	0.56
			Eye	600	0.64	0.25	0.57	0.17	2.26	0.54	0.25	0.47	0.15	2.17	0.84	0.10	0.86	0.14	0.54
	Raja montagui	Spotted skate	Gills	100	0.65	0.12	0.65	0.13	0.76	0.43	0.07	0.42	0.11	0.29	0.67	0.14	0.64	0.19	0.66
			Liver	280	0.60	0.13	0.60	0.18	0.95	0.41	0.10	0.40	0.11	0.56	0.71	0.15	0.69	0.22	0.64
			Peritoneum	300	0.65	0.13	0.65	0.19	0.71	0.43	0.09	0.43	0.13	0.49	0.68	0.16	0.67	0.26	0.68
			Skin	300	0.59	0.12	0.60	0.17	0.65	0.39	0.07	0.39	0.09	0.41	0.68	0.15	0.66	0.21	0.66
			Eye	600	0.73	0.20	0.69	0.18	1.41	0.57	0.22	0.52	0.18	1.33	0.77	0.15	0.80	0.22	0.71
	Leucoraja naevus	Cuckoo skate	Gills	300	0.65	0.09	0.66	0.12	0.34	0.41	0.07	0.40	0.06	0.28	0.65	0.13	0.63	0.15	0.51
			Liver	50	0.47	0.09	0.49	0.13	0.42	0.29	0.05	0.28	0.07	0.20	0.62	0.13	0.60	0.20	0.56
			Skin	25	0.56	0.12	0.57	0.16	0.62	0.36	0.06	0.36	0.09	0.36	0.66	0.14	0.64	0.21	0.65
			Eye	600	0.71	0.20	0.66	0.25	1.14	0.55	0.20	0.50	0.23	1.12	0.77	0.15	0.78	0.23	0.65
Sarcopterygii	*Protopterus annectens*	West African Lungfish	Gills	115	1.05	0.19	1.03	0.21	1.06	0.84	0.18	0.84	0.28	0.77	0.80	0.12	0.81	0.16	0.47
			Liver	123	0.92	0.20	0.94	0.26	0.99	0.77	0.20	0.78	0.31	0.91	0.83	0.10	0.85	0.14	0.51
			Spleen	300	0.80	0.18	0.78	0.24	1.03	0.60	0.18	0.55	0.24	0.96	0.75	0.14	0.76	0.22	0.58
			Kidney	300	0.98	0.18	0.97	0.22	1.02	0.76	0.13	0.75	0.17	0.71	0.79	0.13	0.81	0.19	0.60
			Peritoneum	240	0.98	0.16	0.99	0.21	0.79	0.78	0.14	0.77	0.18	0.73	0.80	0.13	0.82	0.21	0.53
			Skin	300	0.69	0.13	0.67	0.17	0.94	0.54	0.10	0.54	0.14	0.59	0.80	0.12	0.80	0.19	0.53
			Eye	600	1.27	0.45	1.16	0.57	2.78	0.56	0.16	0.54	0.22	1.04	0.50	0.23	0.49	0.41	0.88
			Gut	300	0.89	0.21	0.86	0.28	1.07	0.71	0.20	0.70	0.28	0.94	0.80	0.14	0.83	0.19	0.60
			Lung	300	0.71	0.13	0.70	0.17	0.70	0.52	0.12	0.50	0.15	0.70	0.74	0.13	0.73	0.19	0.56
Actinopterygii	*Anguilla Rostrata*	American eel	Spleen	150	0.52	0.09	0.52	0.13	0.41	0.35	0.06	0.35	0.06	0.31	0.69	0.13	0.69	0.18	0.63
			Kidney	181	0.56	0.11	0.57	0.14	0.56	0.39	0.07	0.38	0.10	0.33	0.71	0.13	0.71	0.19	0.59
			Peritoneum	260	0.56	0.09	0.56	0.13	0.52	0.41	0.06	0.41	0.09	0.33	0.74	0.12	0.73	0.17	0.51
			Skin	300	0.58	0.09	0.58	0.14	0.53	0.42	0.07	0.42	0.09	0.40	0.73	0.12	0.73	0.16	0.64
			Eye	600	0.81	0.23	0.79	0.27	1.61	0.40	0.11	0.38	0.17	0.49	0.53	0.21	0.54	0.35	0.88
	Merlangius merlangus	Whiting	Spleen	230	0.53	0.08	0.52	0.10	0.58	0.40	0.06	0.39	0.09	0.40	0.76	0.12	0.76	0.16	0.58
			Kidney	265	0.56	0.08	0.56	0.12	0.51	0.43	0.07	0.44	0.09	0.33	0.79	0.10	0.79	0.12	0.52
			Peritoneum	300	0.54	0.08	0.53	0.11	0.42	0.40	0.06	0.40	0.08	0.32	0.75	0.13	0.75	0.20	0.63
			Skin	300	0.52	0.07	0.52	0.09	0.53	0.40	0.05	0.40	0.07	0.31	0.78	0.11	0.77	0.15	0.51
			Eye	600	1.16	0.61	0.99	0.98	2.84	0.34	0.12	0.31	0.14	0.71	0.42	0.29	0.29	0.52	0.95
	Scomber scombrus	Atlantic mackerel	Spleen	200	0.53	0.10	0.53	0.13	0.48	0.37	0.07	0.36	0.10	0.42	0.71	0.13	0.71	0.18	0.57
			Kidney	300	0.54	0.11	0.54	0.14	0.65	0.38	0.08	0.38	0.12	0.38	0.72	0.14	0.72	0.22	0.64
			Peritoneum	300	0.57	0.11	0.56	0.14	0.62	0.37	0.07	0.37	0.11	0.44	0.66	0.13	0.65	0.17	0.58
			Skin	300	0.59	0.08	0.59	0.10	0.52	0.44	0.06	0.44	0.07	0.35	0.76	0.12	0.76	0.18	0.55
			Eye	600	0.92	0.55	0.75	0.35	4.30	0.41	0.11	0.40	0.16	0.65	0.55	0.25	0.59	0.43	0.94
	Dicentrarchus labrax	European seabass	Spleen	250	0.51	0.08	0.52	0.11	0.46	0.37	0.06	0.37	0.08	0.29	0.73	0.12	0.72	0.16	0.59
			Kidney	300	0.55	0.09	0.55	0.12	0.60	0.40	0.06	0.40	0.09	0.40	0.73	0.12	0.71	0.15	0.56
			Skin	248	0.57	0.07	0.57	0.09	0.46	0.43	0.06	0.43	0.08	0.35	0.77	0.11	0.76	0.15	0.52
			Eye	600	0.88	0.45	0.74	0.43	4.27	0.40	0.12	0.41	0.20	0.55	0.56	0.26	0.65	0.50	0.95
	Sparus aurata	Gilthead seabream	Spleen	300	0.50	0.07	0.50	0.09	0.50	0.38	0.05	0.37	0.07	0.29	0.76	0.10	0.76	0.14	0.62
			Kidney	300	0.52	0.08	0.52	0.11	0.43	0.39	0.05	0.40	0.07	0.30	0.76	0.11	0.75	0.16	0.54
			Peritoneum	300	0.62	0.09	0.62	0.12	0.59	0.43	0.06	0.43	0.08	0.34	0.71	0.12	0.70	0.18	0.60
			Skin	300	0.50	0.07	0.50	0.08	0.50	0.39	0.05	0.39	0.07	0.26	0.80	0.10	0.80	0.17	0.54
			Eye	600	0.80	0.42	0.66	0.30	3.46	0.41	0.11	0.40	0.17	0.59	0.61	0.26	0.69	0.44	0.93

Supplementary Table 7 - Summary statistics for melanosome geometry for tissues in each major extant taxonomic group studied. IQR, interquartile range; SD, standard deviation.

Tissue	Taxonomic group	Length (µm)					Width (µm)					Aspect ratio				
		Mean	SD	Median	IQR	Range	Mean	SD	median	IQR	range	Mean	SD	median	IQR	range
Skin	Actinopterygii	0.55	0.09	0.55	0.11	0.63	0.42	0.06	0.42	0.08	0.47	0.77	0.11	0.77	0.16	0.64
	Agnatha	0.77	0.17	0.78	0.24	0.85	0.46	0.09	0.45	0.13	0.59	0.62	0.15	0.61	0.19	0.70
	Chondrichthyes	0.59	0.12	0.59	0.15	0.76	0.39	0.07	0.39	0.09	0.47	0.68	0.14	0.66	0.19	0.66
	Sarcopterygii	0.69	0.13	0.67	0.17	0.94	0.54	0.10	0.54	0.14	0.59	0.80	0.12	0.80	0.19	0.53
Olfactory sac	Agnatha	0.72	0.16	0.71	0.21	0.83	0.49	0.09	0.49	0.12	0.68	0.70	0.15	0.69	0.22	0.75
Spleen	Actinopterygii	0.52	0.08	0.51	0.11	0.58	0.37	0.06	0.37	0.08	0.46	0.74	0.12	0.73	0.16	0.63
	Chondrichthyes	0.54	0.12	0.53	0.16	0.92	0.38	0.08	0.37	0.10	0.64	0.72	0.14	0.73	0.21	0.63
	Sarcopterygii	0.80	0.18	0.78	0.24	1.03	0.60	0.18	0.55	0.24	0.96	0.75	0.14	0.76	0.22	0.58
Gills	Chondrichthyes	0.64	0.12	0.64	0.15	0.84	0.42	0.07	0.41	0.09	0.42	0.67	0.13	0.65	0.18	0.67
	Sarcopterygii	1.05	0.19	1.03	0.21	1.06	0.84	0.18	0.84	0.28	0.77	0.80	0.12	0.81	0.16	0.47
Kidney	Actinopterygii	0.55	0.09	0.55	0.12	0.68	0.40	0.07	0.40	0.09	0.41	0.74	0.12	0.74	0.18	0.64
	Sarcopterygii	0.98	0.18	0.97	0.22	1.02	0.76	0.13	0.75	0.17	0.71	0.79	0.13	0.81	0.19	0.60
Peritoneum	Actinopterygii	0.57	0.10	0.56	0.14	0.62	0.40	0.07	0.40	0.09	0.44	0.72	0.13	0.71	0.19	0.63
	Chondrichthyes	0.55	0.13	0.53	0.17	0.80	0.38	0.08	0.37	0.10	0.50	0.71	0.15	0.71	0.23	0.68
	Sarcopterygii	0.98	0.16	0.99	0.21	0.79	0.78	0.14	0.77	0.18	0.73	0.80	0.13	0.82	0.21	0.53
Gut	Chondrichthyes	0.50	0.08	0.50	0.10	0.51	0.35	0.05	0.35	0.06	0.29	0.72	0.14	0.73	0.21	0.61
	Sarcopterygii	0.89	0.21	0.86	0.28	1.07	0.71	0.20	0.70	0.28	0.94	0.80	0.14	0.83	0.19	0.60
Lung	Sarcopterygii	0.71	0.13	0.70	0.17	0.70	0.52	0.12	0.50	0.15	0.70	0.74	0.13	0.73	0.19	0.56
Liver	Chondrichthyes	0.57	0.12	0.57	0.16	0.96	0.38	0.08	0.37	0.09	0.56	0.68	0.14	0.66	0.19	0.69
	Sarcopterygii	0.92	0.20	0.94	0.26	0.99	0.77	0.20	0.78	0.31	0.91	0.83	0.10	0.85	0.14	0.51
Eye	Actinopterygii	0.91	0.49	0.75	0.45	4.38	0.39	0.12	0.38	0.18	0.71	0.53	0.26	0.59	0.47	0.95
	Agnatha	0.75	0.15	0.74	0.18	1.03	0.51	0.09	0.52	0.13	0.57	0.71	0.15	0.71	0.22	0.75
	Chondrichthyes	0.68	0.21	0.64	0.23	2.32	0.54	0.20	0.49	0.20	2.23	0.80	0.13	0.82	0.18	0.71
	Sarcopterygii	1.27	0.45	1.16	0.57	2.78	0.56	0.16	0.54	0.22	1.04	0.50	0.23	0.49	0.41	0.88

Supplementary Table 8 - Shapiro–Wilk and two-sided one-way ANOVA test results for melanosome length, width and aspect ratio among extant species within each tissue studied. ANOVAs were performed using linear models, with species as a fixed effect. Filled cells represent *p* values < 0.05 (statistically significant). F, F-statistic; df1, numerator degrees of freedom; df2, denominator degrees of freedom.

Tissue	Common name	Shapiro-Wilk			ANOVA											
					Length				Width				Aspect ratio			
		Length	Width	Aspect ratio	F	df_1	df_2	*p*	F	df_1	df_2	*p*	F	df_1	df_2	*p*
Gills	Rabbit fish<br>Black mouthed catshark<br>Cuckoo skate<br>Spotted skate<br>West African lungfish	0.68	0.02	0.53	17.06	4	5	4.05E-03	12.56	4	5	8.07E-03	10.08	4	5	1.30E-02
Kidney	West African lungfish<br>American Eel<br>Whiting<br>Atlantic Mackerel<br>European seabass<br>Gilthead seabream	0.38	0.33	0.06	34.66	5	11	2.26E-06	59.70	5	11	1.35E-07	3.11	5	11	5.45E-02
Gut	Rabbit fish<br>West African lungfish	0.67	0.54	0.22	82.28	1	4	8.19E-04	101.18	1	4	5.49E-04	16.00	1	4	1.61E-02
Spleen	Rabbit fish<br>Small-spotted catshark<br>West African lungfish<br>American eel<br>Whiting<br>Atlantic Mackerel<br>European seabass<br>Gilthead seabream	0.65	0.36	0.91	20.26	7	14	2.60E-06	13.18	7	14	3.44E-05	2.41	7	14	7.64E-02
Skin	River lamprey<br>Rabbit fish<br>Blackmouth catshark<br>Small-spotted catshark<br>Cuckoo skate<br>Spotted skate<br>West African lungfish<br>American eel<br>Whiting<br>Atlantic Mackerel<br>European seabass<br>Gilthead seabream	0.76	0.91	0.62	17.18	11	24	8.72E-09	8.05	11	24	1.13E-05	10.44	11	24	1.11E-06
Peritoneum	Rabbit fish<br>Blackmouth catshark<br>Spotted skate<br>West African lungfish<br>American eel<br>Whiting<br>Atlantic Mackerel	0.65	0.82	0.23	80.87	7	16	2.72E-11	74.37	7	16	5.19E-11	3.92	7	16	1.12E-02
Liver	Blackmouth catshark<br>Small-spotted catshark<br>Cuckoo skate<br>Spotted skate<br>West African lungfish	0.82	0.64	0.07	27.86	4	7	2.15E-04	53.62	4	7	2.46E-05	31.20	4	7	1.49E-04
Eye	River lamprey<br>Rabbit fish<br>Blackmouth catshark<br>Small-spotted catshark<br>Cuckoo skate<br>Spotted skate<br>West African lungfish<br>American eel<br>Whiting<br>Atlantic Mackerel<br>European seabass<br>Gilthead seabream	0.48	0.32	0.13	51.32	11	24	6.08E-14	27.59	11	24	5.98E-11	67.89	11	24	2.49E-15

Supplementary Table 9 - Tukey post hoc test results and multivariate Bhattacharyya coefficients for pairwise comparisons of melanosome geometry among extant fish taxa in different tissues. Pairwise comparisons are two-sided and *p* values were adjusted for multiple comparisons using Tukey's method. Bhattacharyya coefficient were computed from length, width, and aspect ratio data. Filled cells represent *p* values < 0.05 (statistically significant).

Tissue	Specimen (Common name)		Post hoc test			Bhattacharyya coefficient
	Group 1	Group 2	Length	Width	Aspect ratio	Length, width and aspect ratio
Gills	Blackmouth catshark	Rabbit fish	0.48	1.00	0.07	0.79
	Blackmouth catshark	Cuckoo skate	1.00	1.00	1.00	0.94
	Blackmouth catshark	West African lungfish	7.71E-03	9.01E-03	0.01	0.15
	Blackmouth catshark	Spotted skate	1.00	1.00	0.89	0.96
	Rabbit fish	Cuckoo skate	0.81	1.00	0.25	0.79
	Rabbit fish	West African lungfish	2.81E-03	8.26E-03	0.23	0.09
	Rabbit fish	Spotted skate	0.82	1.00	0.50	0.86
	Cuckoo skate	West African lungfish	0.02	0.03	0.05	0.10
	Cuckoo skate	Spotted skate	1.00	1.00	0.98	0.89
	West African lungfish	Spotted skate	0.02	0.03	0.08	0.15
Kidney	American eel	West African lungfish	2.93E-05	1.02E-06	0.15	0.07
	American eel	Atlantic Mackerel	1.00	1.00	1.00	0.98
	American eel	European seabass	1.00	1.00	0.96	0.96
	American eel	Gilthead seabream	0.96	1.00	0.50	0.87
	American eel	Whiting	1.00	0.73	0.16	0.83
	West African lungfish	Atlantic Mackerel	6.17E-06	2.61E-07	0.18	0.08
	West African lungfish	European seabass	8.06E-06	4.27E-07	0.34	0.07
	West African lungfish	Gilthead seabream	4.07E-06	3.59E-07	0.91	0.04
	West African lungfish	Whiting	7.70E-06	1.11E-06	1.00	0.08
	Atlantic Mackerel	European seabass	1.00	0.98	1.00	0.96
	Atlantic Mackerel	Gilthead seabream	1.00	1.00	0.61	0.85
	Atlantic Mackerel	Whiting	1.00	0.49	0.18	0.82
	European seabass	Gilthead seabream	0.98	1.00	0.85	0.94
	European seabass	Whiting	1.00	0.84	0.34	0.91
	Gilthead seabream	Whiting	0.98	0.73	0.92	0.91
Gut	West African lungfish	Rabbit fish	8.19E-04	5.49E-04	0.02	0.15
Spleen	American eel	Rabbit fish	1.00	0.98	0.53	0.94
	American eel	West African lungfish	1.97E-05	6.69E-05	0.20	0.27
	American eel	Atlantic Mackerel	1.00	0.99	0.96	0.96
	American eel	European seabass	1.00	0.99	0.64	0.95
	American eel	Gilthead seabream	1.00	0.98	0.11	0.85
	American eel	Small-spotted catshark	0.63	0.69	0.97	0.85
	American eel	Whiting	1.00	0.71	0.13	0.90
	Rabbit fish	West African lungfish	6.66E-06	7.97E-05	0.99	0.38
	Rabbit fish	Atlantic Mackerel	1.00	1.00	0.99	0.98
	Rabbit fish	European seabass	1.00	1.00	1.00	0.92
	Rabbit fish	Gilthead seabream	0.99	1.00	0.91	0.83
	Rabbit fish	Small-spotted catshark	0.61	0.98	0.94	0.95
	Rabbit fish	Whiting	1.00	0.98	0.94	0.94
	West African lungfish	Atlantic Mackerel	3.82E-05	2.40E-04	0.77	0.33
	West African lungfish	European seabass	6.08E-06	7.20E-05	0.96	0.26
	West African lungfish	Gilthead seabream	2.60E-06	8.68E-05	1.00	0.22
	West African lungfish	Small-spotted catshark	9.11E-05	3.32E-04	0.56	0.52
	West African lungfish	Whiting	1.19E-05	3.10E-04	1.00	0.33
	Atlantic Mackerel	European seabass	1.00	1.00	1.00	0.97
	Atlantic Mackerel	Gilthead seabream	0.97	1.00	0.56	0.88
	Atlantic Mackerel	Small-spotted catshark	0.87	0.98	1.00	0.92
	Atlantic Mackerel	Whiting	1.00	0.99	0.62	0.96
	European seabass	Gilthead seabream	1.00	1.00	0.83	0.96
	European seabass	Small-spotted catshark	0.56	0.97	0.98	0.81
	European seabass	Whiting	1.00	0.98	0.88	0.96
	Gilthead seabream	Small-spotted catshark	0.24	0.99	0.34	0.70
	Gilthead seabream	Whiting	0.93	0.99	1.00	0.92
	Small-spotted catshark	Whiting	0.84	1.00	0.40	0.87
Liver	Blackmouth catshark	Cuckoo skate	0.24	0.31	0.93	0.57
	Blackmouth catshark	West African lungfish	6.41E-04	3.84E-05	1.15E-04	0.18
	Blackmouth catshark	Small-spotted catshark	0.52	1.00	0.06	0.86
	Blackmouth catshark	Spotted skate	1.00	0.64	0.02	0.86
	Cuckoo skate	West African lungfish	5.50E-04	7.25E-05	4.32E-04	0.06
	Cuckoo skate	Small-spotted catshark	0.76	0.39	0.08	0.76
	Cuckoo skate	Spotted skate	0.23	0.09	0.04	0.54
	West African lungfish	Small-spotted catshark	2.17E-04	3.38E-05	1.12E-03	0.18
	West African lungfish	Spotted skate	6.50E-04	7.94E-05	2.03E-03	0.33
	Small-spotted catshark	Spotted skate	0.51	0.50	0.94	0.88

Supplementary Table 9 (continued)

Tissue	Specimen (Common name)		Post hoc test			Bhattacharyya coefficient
	Group 1	Group 2	Length	Width	Aspect ratio	Length, width and aspect ratio
Peritoneum	American eel	Blackmouth catshark	0.14	0.23	1.00	0.78
	American eel	Rabbit fish	0.31	0.42	1.00	0.84
	American eel	West African lungfish	2.86E-10	5.72E-10	0.32	0.06
	American eel	Atlantic Mackerel	1.00	0.73	0.36	0.88
	American eel	Gilthead seabream	0.36	0.96	1.00	0.89
	American eel	Spotted skate	0.03	0.93	0.74	0.69
	American eel	Whiting	0.98	1.00	1.00	0.97
	Blackmouth catshark	Rabbit fish	1.00	1.00	1.00	0.96
	Blackmouth catshark	West African lungfish	2.82E-11	5.85E-11	0.18	0.02
	Blackmouth catshark	Atlantic Mackerel	0.09	0.97	0.57	0.82
	Blackmouth catshark	Gilthead seabream	1.88E-03	0.03	1.00	0.56
	Blackmouth catshark	Spotted skate	1.20E-04	0.03	0.91	0.49
	Blackmouth catshark	Whiting	0.53	0.35	0.97	0.83
	Rabbit fish	West African lungfish	4.01E-11	8.15E-11	0.21	0.03
	Rabbit fish	Atlantic Mackerel	0.22	1.00	0.52	0.89
	Rabbit fish	Gilthead seabream	4.99E-03	0.08	1.00	0.66
	Rabbit fish	Spotted skate	2.96E-04	0.06	0.88	0.58
	Rabbit fish	Whiting	0.81	0.59	0.98	0.88
	West African lungfish	Atlantic Mackerel	3.55E-10	1.29E-10	5.17E-03	0.06
	West African lungfish	Gilthead seabream	2.72E-09	1.66E-09	0.10	0.08
	West African lungfish	Spotted skate	1.33E-08	1.84E-09	0.02	0.18
	West African lungfish	Whiting	1.26E-10	4.30E-10	0.65	0.04
	Atlantic Mackerel	Gilthead seabream	0.49	0.20	0.76	0.81
	Atlantic Mackerel	Spotted skate	0.04	0.17	1.00	0.76
	Atlantic Mackerel	Whiting	0.94	0.88	0.14	0.83
	Gilthead seabream	Spotted skate	0.80	1.00	0.98	0.83
	Gilthead seabream	Whiting	0.08	0.86	0.88	0.80
	Spotted skate	Whiting	4.77E-03	0.81	0.40	0.60
Skin	American eel	Blackmouth catshark	0.08	1.00	0.05	0.80
	American eel	Rabbit fish	0.98	0.92	1.00	0.92
	American eel	Cuckoo skate	1.00	0.37	0.30	0.84
	American eel	River River lamprey	7.68E-06	0.77	0.01	0.53
	American eel	West African lungfish	0.02	1.92E-03	0.55	0.67
	American eel	Atlantic Mackerel	1.00	0.99	1.00	0.94
	American eel	European seabass	1.00	1.00	0.95	0.89
	American eel	Gilthead seabream	0.17	0.99	0.55	0.77
	American eel	Small-spotted catshark	1.00	0.92	0.97	0.95
	American eel	Spotted skate	1.00	0.99	0.75	0.91
	American eel	Whiting	0.48	1.00	0.80	0.77
	Blackmouth catshark	Rabbit fish	4.73E-03	0.90	0.19	0.67
	Blackmouth catshark	Cuckoo skate	0.02	0.34	1.00	0.71
	Blackmouth catshark	River River lamprey	0.02	0.80	1.00	0.71
	Blackmouth catshark	West African lungfish	1.00	2.22E-03	2.50E-04	0.61
	Blackmouth catshark	Atlantic Mackerel	0.22	0.99	5.95E-03	0.73
	Blackmouth catshark	European seabass	0.04	1.00	1.84E-03	0.58
	Blackmouth catshark	Gilthead seabream	6.92E-05	0.99	2.59E-04	0.42
	Blackmouth catshark	Small-spotted catshark	0.01	0.90	0.48	0.72
	Blackmouth catshark	Spotted skate	0.23	0.99	0.84	0.83
	Blackmouth catshark	Whiting	3.41E-04	1.00	6.91E-04	0.44
	Rabbit fish	Cuckoo skate	1.00	1.00	0.71	0.91
	Rabbit fish	River River lamprey	5.12E-07	0.08	0.05	0.39
	Rabbit fish	West African lungfish	1.18E-03	5.19E-05	0.19	0.46
	Rabbit fish	Atlantic Mackerel	0.81	0.33	0.89	0.80
	Rabbit fish	European seabass	1.00	0.51	0.63	0.84
	Rabbit fish	Gilthead seabream	0.82	1.00	0.20	0.87
	Rabbit fish	Small-spotted catshark	1.00	1.00	1.00	0.98
	Rabbit fish	Spotted skate	0.80	1.00	0.98	0.87
	Rabbit fish	Whiting	0.99	1.00	0.38	0.85
	Cuckoo skate	River River lamprey	1.71E-06	8.52E-03	0.89	0.46
	Cuckoo skate	West African lungfish	4.54E-03	5.03E-06	2.49E-03	0.44
	Cuckoo skate	Atlantic Mackerel	0.98	0.05	0.05	0.65
	Cuckoo skate	European seabass	1.00	0.09	0.02	0.65
	Cuckoo skate	Gilthead seabream	0.49	0.95	2.58E-03	0.70
	Cuckoo skate	Small-spotted catshark	1.00	1.00	0.96	0.91
	Cuckoo skate	Spotted skate	0.98	0.94	1.00	0.94
	Cuckoo skate	Whiting	0.87	0.81	6.82E-03	0.66
	River River lamprey	West African lungfish	0.08	0.13	5.20E-05	0.69
	River River lamprey	Atlantic Mackerel	2.74E-05	1.00	1.22E-03	0.46
	River River lamprey	European seabass	3.98E-06	0.99	3.73E-04	0.38
	River River lamprey	Gilthead seabream	1.49E-08	0.18	5.39E-05	0.27
	River River lamprey	Small-spotted catshark	1.12E-06	0.08	0.17	0.45
	River River lamprey	Spotted skate	2.81E-05	0.19	0.45	0.62
	River River lamprey	Whiting	5.48E-08	0.33	1.41E-04	0.27
	West African lungfish	Atlantic Mackerel	0.07	0.03	0.97	0.64
	West African lungfish	European seabass	0.01	0.01	1.00	0.55
	West African lungfish	Gilthead seabream	1.80E-05	1.31E-04	1.00	0.39
	West African lungfish	Small-spotted catshark	2.86E-03	4.98E-05	0.06	0.53
	West African lungfish	Spotted skate	0.08	1.50E-04	0.02	0.63
	West African lungfish	Whiting	8.60E-05	3.26E-04	1.00	0.39
	Atlantic Mackerel	European seabass	1.00	1.00	1.00	0.91
	Atlantic Mackerel	Gilthead seabream	0.06	0.55	0.97	0.67
	Atlantic Mackerel	Small-spotted catshark	0.95	0.32	0.56	0.82
	Atlantic Mackerel	Spotted skate	1.00	0.59	0.24	0.77
	Atlantic Mackerel	Whiting	0.21	0.78	1.00	0.71
	European seabass	Gilthead seabream	0.28	0.75	1.00	0.82
	European seabass	Small-spotted catshark	1.00	0.50	0.29	0.86
	European seabass	Spotted skate	1.00	0.78	0.10	0.71
	European seabass	Whiting	0.67	0.92	1.00	0.88
	Gilthead seabream	Small-spotted catshark	0.61	1.00	0.06	0.83
	Gilthead seabream	Spotted skate	0.05	1.00	0.02	0.68
	Gilthead seabream	Whiting	1.00	1.00	1.00	0.98
	Small-spotted catshark	Spotted skate	0.94	1.00	1.00	0.90
	Small-spotted catshark	Whiting	0.94	1.00	0.14	0.82
	Spotted skate	Whiting	0.20	1.00	0.04	0.65

Supplementary Table 9 (continued)

Tissue	Specimen (Common name)		Post hoc test			Bhattacharyya coefficient
	Group 1	Group 2	Length	Width	Aspect ratio	Length, width and aspect ratio
Eye	American eel	Blackmouth catshark	9.14E-03	5.13E-05	2.20E-10	0.20
	American eel	Rabbit fish	7.28E-04	0.14	1.42E-08	0.36
	American eel	Cuckoo skate	0.31	2.64E-05	3.37E-08	0.41
	American eel	River River lamprey	0.84	1.17E-03	8.43E-06	0.62
	American eel	West African lungfish	2.12E-09	1.64E-05	1.00	0.37
	American eel	Atlantic Mackerel	0.36	1.00	1.00	0.69
	American eel	European seabass	0.88	1.00	0.99	0.75
	American eel	Gilthead seabream	1.00	1.00	0.09	0.77
	American eel	Small-spotted catshark	0.32	1.30E-06	1.43E-09	0.28
	American eel	Spotted skate	0.62	4.46E-06	3.49E-08	0.37
	American eel	Whiting	4.12E-07	0.34	5.25E-03	0.60
	Blackmouth catshark	Rabbit fish	0.99	0.07	0.47	0.80
	Blackmouth catshark	Cuckoo skate	0.85	1.00	0.24	0.78
	Blackmouth catshark	River River lamprey	0.32	0.97	7.08E-04	0.44
	Blackmouth catshark	West African lungfish	2.27E-12	1.00	4.25E-11	0.13
	Blackmouth catshark	Atlantic Mackerel	2.07E-05	1.78E-04	9.48E-10	0.14
	Blackmouth catshark	European seabass	1.85E-04	8.57E-05	1.65E-09	0.16
	Blackmouth catshark	Gilthead seabream	0.02	1.51E-04	9.33E-08	0.18
	Blackmouth catshark	Small-spotted catshark	0.84	0.88	0.99	0.80
	Blackmouth catshark	Spotted skate	0.54	0.99	0.23	0.81
	Blackmouth catshark	Whiting	1.32E-10	1.74E-07	3.20E-13	0.11
	Rabbit fish	Cuckoo skate	0.25	0.04	1.00	0.91
	Rabbit fish	River River lamprey	0.04	0.61	0.15	0.67
	Rabbit fish	West African lungfish	5.87E-13	0.02	2.18E-09	0.17
	Rabbit fish	Atlantic Mackerel	1.92E-06	0.35	7.66E-08	0.24
	Rabbit fish	European seabass	1.55E-05	0.22	1.45E-07	0.27
	Rabbit fish	Gilthead seabream	1.78E-03	0.32	1.52E-05	0.31
	Rabbit fish	Small-spotted catshark	0.25	1.69E-03	0.98	0.85
	Rabbit fish	Spotted skate	0.10	6.46E-03	1.00	0.88
	Rabbit fish	Whiting	2.49E-11	4.00E-04	6.91E-12	0.20
	Cuckoo skate	River River lamprey	1.00	0.90	0.32	0.72
	Cuckoo skate	West African lungfish	2.67E-11	1.00	4.89E-09	0.25
	Cuckoo skate	Atlantic Mackerel	1.22E-03	9.04E-05	1.90E-07	0.26
	Cuckoo skate	European seabass	0.01	4.38E-05	3.66E-07	0.29
	Cuckoo skate	Gilthead seabream	0.52	7.68E-05	4.28E-05	0.32
	Cuckoo skate	Small-spotted catshark	1.00	0.96	0.85	0.85
	Cuckoo skate	Spotted skate	1.00	1.00	1.00	0.99
	Cuckoo skate	Whiting	2.43E-09	9.76E-08	1.33E-11	0.21
	River River lamprey	West African lungfish	1.14E-10	0.81	8.84E-07	0.28
	River River lamprey	Atlantic Mackerel	0.01	4.12E-03	6.22E-05	0.37
	River River lamprey	European seabass	0.09	1.97E-03	1.32E-04	0.41
	River River lamprey	Gilthead seabream	0.96	3.49E-03	0.02	0.43
	River River lamprey	Small-spotted catshark	1.00	0.19	9.89E-03	0.60
	River River lamprey	Spotted skate	1.00	0.46	0.33	0.68
	River River lamprey	Whiting	1.32E-08	2.81E-06	8.98E-10	0.29
	West African lungfish	Atlantic Mackerel	2.91E-07	5.56E-05	0.76	0.55
	West African lungfish	European seabass	4.45E-08	2.71E-05	0.56	0.50
	West African lungfish	Gilthead seabream	1.10E-09	4.73E-05	9.44E-03	0.44
	West African lungfish	Small-spotted catshark	2.72E-11	0.99	2.51E-10	0.15
	West African lungfish	Spotted skate	6.10E-11	1.00	5.06E-09	0.23
	West African lungfish	Whiting	0.27	6.46E-08	0.05	0.52
	Atlantic Mackerel	European seabass	1.00	1.00	1.00	0.97
	Atlantic Mackerel	Gilthead seabream	0.19	1.00	0.41	0.93
	Atlantic Mackerel	Small-spotted catshark	1.25E-03	4.13E-06	6.79E-09	0.18
	Atlantic Mackerel	Spotted skate	4.35E-03	1.47E-05	1.97E-07	0.24
	Atlantic Mackerel	Whiting	1.30E-04	0.14	6.57E-04	0.94
	European seabass	Gilthead seabream	0.69	1.00	0.60	0.98
	European seabass	Small-spotted catshark	0.01	2.09E-06	1.23E-08	0.20
	European seabass	Spotted skate	0.04	7.28E-06	3.81E-07	0.26
	European seabass	Whiting	1.48E-05	0.24	3.06E-04	0.92
	Gilthead seabream	Small-spotted catshark	0.53	3.54E-06	9.22E-07	0.21
	Gilthead seabream	Spotted skate	0.83	1.25E-05	4.48E-05	0.28
	Gilthead seabream	Whiting	1.91E-07	0.16	2.17E-06	0.88
	Small-spotted catshark	Spotted skate	1.00	1.00	0.84	0.85
	Small-spotted catshark	Whiting	2.48E-09	7.32E-09	1.25E-12	0.14
	Spotted skate	Whiting	6.35E-09	2.10E-08	1.36E-11	0.19

Supplementary Table 10 - Summary statistics, Shapiro–Wilk and two-sided ANOVA test results for geometry data for ocular melanosomes in extant and fossil fish. Data are for melanosome populations identified using Gaussian mixture modelling. ANOVA tests performed using linear mixed-effects models. Observed combinations of taxonomic group, preservation state (extant or fossil) and melanosome population were modelled as the fixed effect, with species included as a random effect to account for variation among species. Filled cells represent *p* values < 0.05 (statistically significant). IQR, interquartile range; SD, Standard deviation; F, F-statistic; df1, numerator degrees of freedom; df2, denominator degrees of freedom.

Taxonomic group	Specimen type	Population	Length (µm)					Width (µm)					Aspect ratio				
			Mean	SD	Median	IQR	Range	Mean	SD	Median	IQR	Range	Mean	SD	Median	IQR	Range
Agnatha	Extant	1	0.75	0.15	0.74	0.18	1.03	0.51	0.09	0.52	0.13	0.57	0.71	0.15	0.71	0.22	0.75
Agnatha	Fossil	1	0.88	0.17	0.87	0.24	1.04	0.54	0.10	0.54	0.13	0.62	0.63	0.14	0.60	0.20	0.70
Chondrichthyes	Extant	1	0.68	0.21	0.64	0.23	2.32	0.54	0.20	0.49	0.20	2.23	0.80	0.13	0.82	0.18	0.71
Chondrichthyes	Fossil	1	0.78	0.21	0.72	0.29	1.21	0.65	0.18	0.61	0.25	0.91	0.84	0.10	0.86	0.13	0.60
Acanthodii	Fossil	1	0.98	0.12	0.99	0.16	0.63	0.75	0.09	0.75	0.11	0.53	0.77	0.09	0.77	0.12	0.45
Acanthodii	Fossil	2	1.61	0.32	1.56	0.46	1.56	0.46	0.06	0.45	0.08	0.40	0.30	0.08	0.29	0.13	0.39
Sarcopterygii	Extant	1	0.97	0.19	0.95	0.26	1.34	0.66	0.13	0.64	0.15	0.88	0.70	0.14	0.69	0.22	0.55
Sarcopterygii	Extant	2	1.62	0.42	1.53	0.47	2.37	0.43	0.08	0.42	0.11	0.42	0.29	0.09	0.28	0.13	0.33
Sarcopterygii	Fossil	1	0.84	0.21	0.86	0.30	1.44	0.55	0.12	0.53	0.15	0.94	0.68	0.15	0.67	0.23	0.63
Sarcopterygii	Fossil	2	1.64	0.42	1.55	0.49	2.18	0.35	0.06	0.35	0.08	0.36	0.23	0.06	0.22	0.09	0.29
Actinopterygii	Extant	1	0.65	0.13	0.64	0.17	1.13	0.46	0.09	0.46	0.13	0.71	0.72	0.14	0.73	0.19	0.71
Actinopterygii	Extant	2	1.30	0.55	1.15	0.64	4.14	0.29	0.06	0.29	0.08	0.39	0.26	0.11	0.24	0.17	0.48
Actinopterygii	Fossil	1	0.73	0.12	0.73	0.17	0.73	0.57	0.09	0.56	0.11	0.47	0.78	0.10	0.79	0.12	0.52
Actinopterygii	Fossil	2	1.35	0.29	1.31	0.36	2.02	0.32	0.04	0.32	0.05	0.29	0.25	0.06	0.25	0.09	0.34

Shapiro-Wilk			ANOVA											
			Length				Width				Aspect ratio			
Length	Width	Aspect ratio	F	df_1	df_2	*p*	F	df_1	df_2	*p*	F	df_1	df_2	*p*
0.21	0.98	5.22E-05	30.51	1	13	1.00E-04	55.69	1	13	1.00E-04	207.61	1	13	1.00E-04

Supplementary Table 11 - Tukey post hoc test results and multivariate Bhattacharyya coefficients for pairwise comparisons of ocular melanosome geometry between melanosome populations. Pairwise comparisons are two-sided and *p* values are adjusted for multiple comparisons using Tukey's method. Bhattacharyya coefficient was computed from length, width, and aspect ratio data. Filled cells represent *p* values < 0.05 (statistically significant).

Taxonomic group, state (fossil/extant) and melanosome population		post hoc test			Bhattacharyya coefficient
Group 1	Group 2	Length	Width	Aspect ratio	Length, width and aspect ratio
Acanthodii fossil population 1	Acanthodii fossil population 2	8.18E-03	2.92E-10	3.61E-12	3.41E-03
Acanthodii fossil population 1	Actinopterygii extant population 1	5.82E-06	3.28E-06	1.00	0.17
Acanthodii fossil population 1	Actinopterygii extant population 2	4.44E-03	5.00E-12	3.80E-12	1.04E-03
Acanthodii fossil population 1	Actinopterygii fossil population 1	0.01	0.09	1.00	0.38
Acanthodii fossil population 1	Actinopterygii fossil population 2	0.12	2.95E-09	2.17E-10	2.08E-04
Acanthodii fossil population 1	Agnatha extant population 1	0.11	0.05	1.00	0.28
Acanthodii fossil population 1	Agnatha fossil population 1	0.88	0.08	0.55	0.37
Acanthodii fossil population 1	Chondrichthyes extant population 1	4.31E-03	0.03	1.00	0.36
Acanthodii fossil population 1	Chondrichthyes fossil population 1	0.21	0.87	0.98	0.53
Acanthodii fossil population 1	Sarcopterygii extant population 1	1.00	0.95	0.99	0.68
Acanthodii fossil population 1	Sarcopterygii extant population 2	9.11E-04	1.02E-05	3.49E-09	3.46E-03
Acanthodii fossil population 1	Sarcopterygii fossil population 1	0.61	0.04	0.95	0.46
Acanthodii fossil population 1	Sarcopterygii fossil population 2	5.42E-05	2.00E-08	4.55E-11	2.20E-04
Acanthodii fossil population 2	Actinopterygii extant population 1	2.24E-06	1.00	9.55E-11	0.02
Acanthodii fossil population 2	Actinopterygii extant population 2	0.79	4.07E-04	1.00	0.17
Acanthodii fossil population 2	Actinopterygii fossil population 1	3.03E-05	0.52	1.22E-09	3.31E-03
Acanthodii fossil population 2	Actinopterygii fossil population 2	0.98	0.12	1.00	0.33
Acanthodii fossil population 2	Agnatha extant population 1	4.70E-03	0.99	6.08E-04	0.04
Acanthodii fossil population 2	Agnatha fossil population 1	0.02	0.88	3.65E-03	0.07
Acanthodii fossil population 2	Chondrichthyes extant population 1	1.81E-03	0.49	7.46E-06	6.30E-03
Acanthodii fossil population 2	Chondrichthyes fossil population 1	6.31E-03	0.06	3.82E-05	2.01E-03
Acanthodii fossil population 2	Sarcopterygii extant population 1	7.11E-03	6.77E-03	5.75E-07	0.05
Acanthodii fossil population 2	Sarcopterygii extant population 2	1.00	1.00	1.00	0.87
Acanthodii fossil population 2	Sarcopterygii fossil population 1	4.13E-04	0.75	1.28E-06	0.06
Acanthodii fossil population 2	Sarcopterygii fossil population 2	1.00	0.42	0.99	0.56
Actinopterygii extant population 1	Actinopterygii extant population 2	3.65E-12	3.61E-12	3.61E-12	0.05
Actinopterygii extant population 1	Actinopterygii fossil population 1	0.86	0.28	0.96	0.68
Actinopterygii extant population 1	Actinopterygii fossil population 2	1.65E-06	0.01	3.81E-12	0.02
Actinopterygii extant population 1	Agnatha extant population 1	0.73	0.97	1.00	0.86
Actinopterygii extant population 1	Agnatha fossil population 1	0.01	0.75	0.68	0.65
Actinopterygii extant population 1	Chondrichthyes extant population 1	1.00	0.13	0.23	0.76
Actinopterygii extant population 1	Chondrichthyes fossil population 1	0.39	0.02	0.35	0.61
Actinopterygii extant population 1	Sarcopterygii extant population 1	1.08E-06	7.95E-04	1.00	0.41
Actinopterygii extant population 1	Sarcopterygii extant population 2	1.13E-08	1.00	1.07E-11	0.02
Actinopterygii extant population 1	Sarcopterygii fossil population 1	4.41E-03	0.54	1.00	0.73
Actinopterygii extant population 1	Sarcopterygii fossil population 2	7.59E-11	0.12	3.67E-12	0.01
Actinopterygii extant population 2	Actinopterygii fossil population 1	9.67E-10	1.31E-07	3.63E-12	4.19E-03
Actinopterygii extant population 2	Actinopterygii fossil population 2	1.00	1.00	1.00	0.60
Actinopterygii extant population 2	Agnatha extant population 1	1.75E-04	5.18E-03	1.28E-05	0.06
Actinopterygii extant population 2	Agnatha fossil population 1	1.57E-03	1.43E-03	7.26E-05	0.07
Actinopterygii extant population 2	Chondrichthyes extant population 1	7.20E-06	3.47E-06	4.16E-09	0.01
Actinopterygii extant population 2	Chondrichthyes fossil population 1	3.02E-04	6.16E-05	7.81E-07	2.68E-03
Actinopterygii extant population 2	Sarcopterygii extant population 1	8.30E-04	9.56E-11	7.99E-12	0.03
Actinopterygii extant population 2	Sarcopterygii extant population 2	0.53	2.75E-03	1.00	0.39
Actinopterygii extant population 2	Sarcopterygii fossil population 1	4.85E-07	6.33E-07	1.10E-11	0.06
Actinopterygii extant population 2	Sarcopterygii fossil population 2	0.24	0.82	1.00	0.54
Actinopterygii fossil population 1	Actinopterygii fossil population 2	2.44E-05	4.69E-12	3.61E-12	8.68E-04
Actinopterygii fossil population 1	Agnatha extant population 1	1.00	1.00	0.97	0.69
Actinopterygii fossil population 1	Agnatha fossil population 1	0.43	1.00	0.32	0.54
Actinopterygii fossil population 1	Chondrichthyes extant population 1	0.99	1.00	1.00	0.69
Actinopterygii fossil population 1	Chondrichthyes fossil population 1	1.00	0.92	1.00	0.76
Actinopterygii fossil population 1	Sarcopterygii extant population 1	0.01	0.87	0.94	0.46
Actinopterygii fossil population 1	Sarcopterygii extant population 2	3.85E-07	0.24	2.27E-10	3.36E-03
Actinopterygii fossil population 1	Sarcopterygii fossil population 1	0.78	1.00	0.79	0.60
Actinopterygii fossil population 1	Sarcopterygii fossil population 2	5.58E-09	1.48E-03	5.88E-12	5.25E-04
Actinopterygii fossil population 2	Agnatha extant population 1	7.58E-03	0.06	1.36E-04	0.03
Actinopterygii fossil population 2	Agnatha fossil population 1	0.04	0.02	7.35E-04	0.05
Actinopterygii fossil population 2	Chondrichthyes extant population 1	1.79E-03	1.51E-03	1.20E-06	3.99E-03
Actinopterygii fossil population 2	Chondrichthyes fossil population 1	0.01	9.35E-04	9.76E-06	4.96E-04
Actinopterygii fossil population 2	Sarcopterygii extant population 1	0.07	3.33E-07	7.42E-09	0.02
Actinopterygii fossil population 2	Sarcopterygii extant population 2	0.93	0.33	1.00	0.48
Actinopterygii fossil population 2	Sarcopterygii fossil population 1	2.31E-03	7.05E-04	1.56E-08	0.03
Actinopterygii fossil population 2	Sarcopterygii fossil population 2	0.80	1.00	1.00	0.71

Supplementary Table 11 (continued)

Taxonomic group, state (fossil/extant) and melanosome population		post hoc test			Bhattacharyya coefficient
Group 1	Group 2	Length	Width	Aspect ratio	Length, width and aspect ratio
Agnatha extant population 1	Agnatha fossil population 1	0.64	1.00	0.96	0.88
Agnatha extant population 1	Chondrichthyes extant population 1	0.96	1.00	0.73	0.65
Agnatha extant population 1	Chondrichthyes fossil population 1	1.00	0.47	0.56	0.59
Agnatha extant population 1	Sarcopterygii extant population 1	0.11	0.37	1.00	0.63
Agnatha extant population 1	Sarcopterygii extant population 2	9.31E-04	0.90	3.58E-04	0.05
Agnatha extant population 1	Sarcopterygii fossil population 1	0.91	1.00	1.00	0.91
Agnatha extant population 1	Sarcopterygii fossil population 2	2.24E-04	0.14	7.82E-05	0.02
Agnatha fossil population 1	Chondrichthyes extant population 1	0.03	1.00	0.06	0.47
Agnatha fossil population 1	Chondrichthyes fossil population 1	0.89	0.66	0.08	0.44
Agnatha fossil population 1	Sarcopterygii extant population 1	0.92	0.55	0.99	0.79
Agnatha fossil population 1	Sarcopterygii extant population 2	3.62E-03	0.64	2.13E-03	0.09
Agnatha fossil population 1	Sarcopterygii fossil population 1	1.00	1.00	1.00	0.92
Agnatha fossil population 1	Sarcopterygii fossil population 2	8.96E-04	0.06	3.94E-04	0.04
Chondrichthyes extant population 1	Chondrichthyes fossil population 1	0.71	0.43	1.00	0.90
Chondrichthyes extant population 1	Sarcopterygii extant population 1	2.46E-03	0.31	0.56	0.49
Chondrichthyes extant population 1	Sarcopterygii extant population 2	2.84E-04	0.22	3.46E-06	8.28E-03
Chondrichthyes extant population 1	Sarcopterygii fossil population 1	0.09	1.00	0.32	0.63
Chondrichthyes extant population 1	Sarcopterygii fossil population 2	5.65E-05	4.65E-03	7.13E-07	2.33E-03
Chondrichthyes fossil population 1	Sarcopterygii extant population 1	0.22	1.00	0.44	0.56
Chondrichthyes fossil population 1	Sarcopterygii extant population 2	1.29E-03	0.03	2.27E-05	2.22E-03
Chondrichthyes fossil population 1	Sarcopterygii fossil population 1	0.99	0.80	0.28	0.61
Chondrichthyes fossil population 1	Sarcopterygii fossil population 2	3.13E-04	2.01E-03	6.18E-06	2.72E-04
Sarcopterygii extant population 1	Sarcopterygii extant population 2	2.03E-04	1.67E-09	3.61E-12	0.06
Sarcopterygii extant population 1	Sarcopterygii fossil population 1	0.67	0.70	1.00	0.85
Sarcopterygii extant population 1	Sarcopterygii fossil population 2	1.97E-05	2.57E-06	1.34E-09	0.02
Sarcopterygii extant population 2	Sarcopterygii fossil population 1	1.01E-05	0.43	2.90E-07	0.07
Sarcopterygii extant population 2	Sarcopterygii fossil population 2	1.00	0.74	1.00	0.76
Sarcopterygii fossil population 1	Sarcopterygii fossil population 2	5.07E-08	7.34E-10	3.61E-12	0.03

Supplementary Table 12 - Summary statistics, Shapiro–Wilk and ANOVA test results for ocular melanosome geometry in fossil fish. Filled cells represent *p* values < 0.05 (statistically significant). IQR, Interquartile range; SD, standard deviation; F, F-statistic; df1, numerator degrees of freedom; df2, denominator degrees of freedom; n, number of melanosomes measured.

Taxonomic group	Scientific name	Specimen number	n	Length (µm)					Width (µm)					Aspect ratio					Shapiro-Wilk			ANOVA											
				Mean	SD	Median	IQR	Range	Mean	SD	Median	IQR	Range	Mean	SD	Median	IQR	Range	Length	Width	Aspect ratio	Length				Width				Aspect ratio			
																						F	df_1	df_2	p-value	F	df_1	df_2	p-value	F	df_1	df_2	p-value
Agnatha	*Mayomyzon pieckoensis*	PF5688	200	0.95	0.14	0.95	0.16	0.89	0.55	0.07	0.55	0.10	0.38	0.59	0.11	0.57	0.12	0.58															
	Mayomyzon pieckoensis	PF10788	200	0.98	0.15	0.99	0.21	0.92	0.59	0.08	0.59	0.10	0.55	0.61	0.11	0.59	0.13	0.62															
	Mayomyzon pieckoensis	PF17992	200	0.85	0.14	0.84	0.15	0.87	0.46	0.09	0.44	0.09	0.50	0.54	0.10	0.52	0.12	0.60															
	Gilpichthyes Greenei	PF8448	200	0.72	0.11	0.71	0.16	0.59	0.55	0.10	0.55	0.12	0.54	0.78	0.11	0.78	0.16	0.51															
Chondrichthyes	*Bandringa sp.*	PF16367	200	0.67	0.13	0.66	0.14	0.82	0.58	0.12	0.57	0.15	0.70	0.87	0.10	0.89	0.11	0.56															
	Bandringa rayi	PF3197	200	0.84	0.20	0.79	0.33	0.81	0.72	0.18	0.66	0.30	0.77	0.85	0.09	0.87	0.10	0.53															
	Bandringa rayi	PF10265	200	0.81	0.23	0.77	0.34	1.12	0.65	0.21	0.62	0.34	0.91	0.80	0.11	0.80	0.15	0.60															
Acanthodii	*Acanthodii* indet.	PF5754	200	1.32	0.43	1.22	0.70	1.87	0.58	0.14	0.56	0.26	0.48	0.51	0.24	0.46	0.45	0.83															
	Acanthodii indet.	PF16201	200	1.30	0.36	1.22	0.49	1.91	0.61	0.19	0.56	0.36	0.73	0.53	0.26	0.42	0.50	0.84	0.94	0.39	1.57E-03	14.545	4	12	1.49E-04	10.2	4	12	7.77E-04	25.366	4	12	8.91E-06
Sarcopterygii	*Esconichthys apopyris*	PF8719	200	1.38	0.57	1.15	0.78	2.38	0.51	0.18	0.50	0.29	0.96	0.46	0.26	0.49	0.51	0.86															
	Esconichthys apopyris	PF9740	200	1.17	0.44	1.11	0.62	2.56	0.47	0.15	0.46	0.23	0.62	0.48	0.26	0.48	0.46	0.89															
	Esconichthys apopyris	PF9868	200	1.07	0.40	1.03	0.68	1.75	0.41	0.09	0.41	0.12	0.67	0.46	0.23	0.42	0.36	0.86															
	Esconichthys apopyris	PF16850	200	1.24	0.57	1.09	0.80	2.54	0.44	0.10	0.43	0.16	0.46	0.46	0.27	0.44	0.44	0.89															
Actinopterygii	*Elonichthys peltigerus*	PF7487	200	1.09	0.35	0.95	0.50	2.08	0.48	0.16	0.46	0.29	0.56	0.51	0.27	0.50	0.52	0.88															
	Elonichthys peltigerus	PF7499	200	1.08	0.39	0.92	0.62	1.78	0.45	0.15	0.45	0.29	0.50	0.51	0.29	0.56	0.56	0.87															
	Elonichthys sp.	PF16083	200	0.96	0.41	0.76	0.63	1.70	0.40	0.10	0.39	0.19	0.41	0.53	0.28	0.51	0.56	0.87															
	Elonichthys sp.	PF16084	200	1.02	0.35	0.93	0.58	1.38	0.45	0.12	0.43	0.22	0.44	0.53	0.28	0.43	0.55	0.81															
