# Supplementary material for: Melanosome evolution shaped sensory biology and terrestrial adaptations in early aquatic vertebrates
Source: Nat Commun. 2026 Oct 27;17:10958. doi: 10.1038/s41467-026-78877-z (PMC13714305; doi:10.1038/s41467-026-78877-z)
Supplement: Supplementary file 2 [file 41467_2026_78877_MOESM2_ESM.pdf]

Description of Additional Supplementary Files

File Name – Supplementary Data 1

Description - Raw melanosome geometry data for length, width and aspect ratio from extant and fossil fish tissues, including Gaussian mixture model populations for ocular tissues.
