# Supplementary material for: Melanosome evolution shaped sensory biology and terrestrial adaptations in early aquatic vertebrates
Source: Nat Commun. 2026 Oct 27;17:10958. doi: 10.1038/s41467-026-78877-z (PMC13714305; doi:10.1038/s41467-026-78877-z)
Supplement: Supplementary file 3 [file 41467_2026_78877_MOESM4_ESM.pdf]

nature portfolio

Corresponding author(s): Daniel J. Cirtina and Maria E. McNamara

Last updated by author(s): Sep 16, 2026

Reporting Summary

Nature Portfolio wishes to improve the reproducibility of the work that we publish. This form provides structure for consistency and transparency in reporting. For further information on Nature Portfolio policies, see our Editorial Policies and the Editorial Policy Checklist.

Statistics

For all statistical analyses, confirm that the following items are present in the figure legend, table legend, main text, or Methods section.

n/a	Confirmed	
☐	☒	The exact sample size (*n*) for each experimental group/condition, given as a discrete number and unit of measurement
☐	☒	A statement on whether measurements were taken from distinct samples or whether the same sample was measured repeatedly
☐	☒	The statistical test(s) used AND whether they are one- or two-sided<br>*Only common tests should be described solely by name; describe more complex techniques in the Methods section.*
☒	☐	A description of all covariates tested
☐	☒	A description of any assumptions or corrections, such as tests of normality and adjustment for multiple comparisons
☐	☒	A full description of the statistical parameters including central tendency (e.g. means) or other basic estimates (e.g. regression coefficient) AND variation (e.g. standard deviation) or associated estimates of uncertainty (e.g. confidence intervals)
☐	☒	For null hypothesis testing, the test statistic (e.g. *F*, *t*, *r*) with confidence intervals, effect sizes, degrees of freedom and *P* value noted<br>*Give P values as exact values whenever suitable.*
☒	☐	For Bayesian analysis, information on the choice of priors and Markov chain Monte Carlo settings
☒	☐	For hierarchical and complex designs, identification of the appropriate level for tests and full reporting of outcomes
☒	☐	Estimates of effect sizes (e.g. Cohen's *d*, Pearson's *r*), indicating how they were calculated

Our web collection on statistics for biologists contains articles on many of the points above.

Software and code

Policy information about availability of computer code

Data collection	Melanosome length and width measurements were made using Fiji distribution package for ImageJ2
Data analysis	All statistical analyses were conducted in R (v4.5.0) using the following packages: tidyverse (v.2.0.0), nlme (v.3.1-171), car (3v.1.3), emmeans (v2.0.4), mclust (v6.1.3) and teigen (v2.2.2).

For manuscripts utilizing custom algorithms or software that are central to the research but not yet described in published literature, software must be made available to editors and reviewers. We strongly encourage code deposition in a community repository (e.g. GitHub). See the Nature Portfolio guidelines for submitting code & software for further information.

Data

Policy information about availability of data

All manuscripts must include a data availability statement. This statement should provide the following information, where applicable:

- Accession codes, unique identifiers, or web links for publicly available datasets
- A description of any restrictions on data availability
- For clinical datasets or third party data, please ensure that the statement adheres to our policy

All data generated and analysed during this study are included in this article and its Supplementary Information files. Processed melanosome measurement data are provided in Supplementary Information. Raw melanosome measurements are provided in Supplementary Data 1.

Research involving human participants, their data, or biological material

Policy information about studies with human participants or human data. See also policy information about sex, gender (identity/presentation), and sexual orientation and race, ethnicity and racism.

Reporting on sex and gender	N/A
Reporting on race, ethnicity, or other socially relevant groupings	N/A
Population characteristics	N/A
Recruitment	N/A
Ethics oversight	N/A

Note that full information on the approval of the study protocol must also be provided in the manuscript.

Field-specific reporting

Please select the one below that is the best fit for your research. If you are not sure, read the appropriate sections before making your selection.

☐ Life sciences ☐ Behavioural & social sciences ☒ Ecological, evolutionary & environmental sciences

For a reference copy of the document with all sections, see nature.com/documents/nr-reporting-summary-flat.pdf

Ecological, evolutionary & environmental sciences study design

All studies must disclose on these points even when the disclosure is negative.

Study description	We examined the morphology of ocular, cutaneous, and internal melanosomes in jawless (Agnatha), cartilaginous (Chondrichthyes), ray-finned (Actinopterygii) and lobe-finned (Sarcopterygii) fishes to assess potential trends between melanosome geometry, anatomical distribution and function across different phylogenetic groups. Thirty-six extant specimens (three replicates of each of twelve species) were used for enzymatic melanin extraction. Additionally, twenty fossil specimens from the Mazon Creek Lagerstätte (Four replicates of each group: Agnatha, Acanthodii, Chondrichthyes, Sarcopterygii and Actinopterygii) were selected to identify any changes in melanosome geometry/function over time.
Research sample	Extant specimens were selected based on local availability, with the goal of assembling a dataset representing a wide phylogenetic diversity. Thirty-six extant specimens (n = three replicates of each of twelve species) were used for enzymatic melanosome extraction and selected to represent four major vertebrate groups : Agnatha, Chondrichthyes, Sarcopterygii and Actinopterygii. Chimaera monstrosa (rabbit fish; Chondrichthyes), Galeus melastomus (blackmouth catshark; Chondrichthyes), Raja montagui (spotted skate; Chondrichthyes) and Leucoraja naevus (cuckoo skate; Chondrichthyes) were caught by the Irish Marine Institute during an annual Anglerfish and Megrim survey. Lampetra fluviatilis (river lamprey; Agnatha), Scyliorhinus canicula (small-spotted catshark; Chondrichthyes) and Anguilla rostrata (American eel; Actinopterygii) were purchased online from UK-based commercial suppliers (Gerry's Fishing, Darwin Biological and Bradley's Fish, respectively). Merlangius merlangus (whiting; Actinopterygii), Scomber scombrus (mackerel; Actinopterygii), Dicentrarchus labrax (sea bass; Actinopterygii) and Sparus aurata (sea bream; Actinopterygii) were purchased from local suppliers (K O'Connell's Fishmongers, English Market, Cork). Protopterus annectens (African lungfish, Sarcopterygii) were supplied by Sims Tropical Fish (UK).<br><br>Fossil specimens were obtained on loan from the Field Museum of Natural History, originating from the Mazon Creek Carboniferous deposits (ca. 306 Ma). Mazon Creek fossils were selected as this fossil site provide representative fossils for all of the extant groups in our study as well as preserving melanosome microbodies, enabling direct comparison of melanosome geometry and chemistry across deep evolutionary lineages. Fossil specimens represent five major vertebrate groups (n = 4 per taxonomic group): Agnatha, Acanthodii, Chondrichthyes, Sarcopterygii and Actinopterygii. Agnathan specimens include three identified as Mayomyzon pieckoensis (PF17992, PF5688, PF10788) and one stem agnathan identified as Gilpichthyes greenei (PF8448). All acanthodian

	specimens (PF16201, PF5754, PF16263, PF11509) are of undetermined affinity. Sarcopterygian specimens are designated as Esconicthys apopyris (PF8719, PF9740, PF9868, PF16850). Three chondrichthyans are identified as Bandringa rayi (PF10265, PF15328, PF3197) and one as an undetermined Bandringa species (PF16367). Among the actinopterygians, two are assigned to Elonichthyes peltigerus (PF7487, PF7499) and two are undetermined Elonichthyes (PF16084, PF16083).
Sampling strategy	No sample size calculation was perfomed. The sample size of extant specimens was limited by species availability and temporal contraints. Three replicates of each extant species was used to assess reproducibility.<br><br>Wherever possible, 100 melanosomes were measured per tissue; for ocular tissues, 200 melanosomes were measured to capture their greater geometric diversity. In samples with sparse melanosomes (n < 100), all available melanosomes were measured.
Data collection	All data collection and analyses by Daniel Cirtina. Scanning electron microscopy (SEM) images were acquired from enzymatic melanosome extracts (extant taxa) and whole speciemen (fossils). Melanosome length and width was measured in Fiji (ImageJ2) with calibration based on the scale bar in each micrograph.
Timing and spatial scale	Data was not collected with a time or spatial scale.
Data exclusions	No data was excluded from the analyses.
Reproducibility	No attempts were made to repeat this study. The methodological approach is outlined in full to ensure complete reproucibility of future studies.
Randomization	Samples were allocated to groups based on taxonomic affiliation (Agnatha, Actinopterygii, Chondrichthyes, Sarcopterygii and Acanthodii) rather than random assignment. All specimens represented adult life stages, and specimen replication was used to account for random variation.
Blinding	Blinding was not implemented during data acquisition or analysis, as samples were grouped by taxonomic affiliation and group identity was intrinsic to the specimens. Broad phylogenetic sampling was required to track changes in melanosome geometry among major vertebrate lineages, making blinding impractical and not relevant to the study design.

Did the study involve field work? ☐ Yes ☒ No

Reporting for specific materials, systems and methods

We require information from authors about some types of materials, experimental systems and methods used in many studies. Here, indicate whether each material, system or method listed is relevant to your study. If you are not sure if a list item applies to your research, read the appropriate section before selecting a response.

Materials & experimental systems

n/a	Involved in the study	
☒	☐	Antibodies
☒	☐	Eukaryotic cell lines
☐	☒	Palaeontology and archaeology
☐	☒	Animals and other organisms
☒	☐	Clinical data
☒	☐	Dual use research of concern
☒	☐	Plants

Methods

n/a	Involved in the study	
☒	☐	ChIP-seq
☒	☐	Flow cytometry
☒	☐	MRI-based neuroimaging

Palaeontology and Archaeology

Specimen provenance	Fossil specimens examined in this study were sourced from the vertebrate collections of the Field Museum of Natural History (FMNH), Chicago, Illinois. Specimens were accessed through a formal, short-term loan (Issued 2 May 2025; FMNH invoice no. G-55337), approved by William F. Simpson and authorised by Arjan Mann. Specimen were temporarily exported from the USA and imported into the UK for study. Permission was granted for non-destructive imaging with scanning-electron microscopy (SEM). No import or export permits were required. No type specimen where included.<br><br>No ethical approval was required for analysis of fossil material. Approval and guidance for the study of fossil material was granted by staff of the FMNH (William F. Simpson, Head of geological collections; Arjan Mann, Assistant Curator of Fossil Fishes and Early Tetrapods).
Specimen deposition	Field Museum of Natural History, Chicago, Illinois.
Dating methods	No new dates are provided for fossil specimens in this study.

☐ Tick this box to confirm that the raw and calibrated dates are available in the paper or in Supplementary Information.

Ethics oversight	No ethical approval or guidance was required as specimens were not recovered in the field.

Note that full information on the approval of the study protocol must also be provided in the manuscript.

Animals and other research organisms

Policy information about studies involving animals; ARRIVE guidelines recommended for reporting animal research, and Sex and Gender in Research

Laboratory animals	N/A
Wild animals	Specimens of Chimaera monstrosa (rabbit fish; Chondrichthyes), Galeus melastomus (blackmouth catshark; Chondrichthyes), Raja montagui (spotted skate; Chondrichthyes) and Leucoraja naevus (cuckoo skate; Chondrichthyes) were caught by the Irish Marine Institute in International Council for the Exploration of the Sea (ICES) subareas 6 (division a, in waters south of 58°N) and 7 (division b, c, j and k, in waters west of 8°W) during an annual Anglerfish and Megrim survey. Specimens were frozen at sea, defrosted immediately after transport to the laboratory and dissected. Lampetra fluviatilis (Eropean river lamprey; Agnatha), Scyliorhinus canicula (small-spotted catshark; Chondrichthyes) and Anguilla rostrata (American eel; Actinopterygii) were purchased online from UK-based commercial suppliers (Gerry’s Fishing, Darwin Biological and Bradley’s Fish, respectively). Specimens were supplied frozen and dissected immediately after defrosting. Merlangius merlangus (whiting; Actinopterygii), Scomber scombrus (Atlantic mackerel; Actinopterygii), Dicentrarchus labrax (Eropean seabass; Actinopterygii) and Sparus aurata (Gilthead seabream; Actinopterygii) were purchased freshly deceased from local suppliers (K O’Connell’s Fishmongers, English Market, Cork). Specimens were dissected within 24 h after death. Protopterus annectens (West African lungfish, Sarcopterygii) were supplied live by Sims Tropical Fish (UK), transported to Ireland by car andeuthanised via cranial concussion on arrival at the laboratory and dissected within 12 h. All specimens analyzed were adult individuals.
Reporting on sex	Sex was not included as a covariate because it could not be reliably determined for all specimens. Samples were collected opportunistically without prior knowledge of sex, and sex-specific analyses were therefore not possible.
Field-collected samples	This study did not involve samples collected from the field.
Ethics oversight	All research reported here was conducted in accordance with the relevant ethical guidelines of University College Cork. West African lungfish specimen (Protopterus annectens) were imported from the UK for scientific purposes under a Movement Approval Notice issued by the Marine Institute Fish Health Unit (OIMP-3346), with entry documented by a Common Health Entry Document for Animals (CHED-A; CHEDA.IE.2023.0004216). Euthanasia was approved by the University College Cork Animal Experimentation Ethics Committee (approval number: UCC-AEEC-23/001; amendment 10/07/2023) and was performed by Luke Harman (School of BEES; HPRA authorisation AE19130/I087). Wild-caught fish specimens were provided to D.C. by the Marine Institute following collection during the 2023 Irish Anglerfish and Megrim Survey (IAMS; cruise CE23003), conducted by the Marine Institute in fulfilment of Ireland's obligations under the Common Fisheries Policy (CFP).

Note that full information on the approval of the study protocol must also be provided in the manuscript.

Plants

Seed stocks	N/A
Novel plant genotypes	N/A
Authentication	N/A
