# Supplementary material for: Melanosome evolution shaped sensory biology and terrestrial adaptations in early aquatic vertebrates
Source: Nat Commun. 2026 Oct 27;17:10958. doi: 10.1038/s41467-026-78877-z (PMC13714305; doi:10.1038/s41467-026-78877-z)
Supplement: Supplementary file 4 [file 41467_2026_78877_MOESM5_ESM.pdf]

nature portfolio

Peer Review File

Melanosome evolution shaped sensory biology and terrestrial adaptations in early aquatic vertebrates

Corresponding Author: Mr Daniel Cirtina

This file contains all reviewer reports in order by version, followed by all author rebuttals in order by version.

Version 0:

Reviewer comments:

Reviewer #1

(Remarks to the Author)
The submitted manuscript reports on an impressive dataset and a robust statistical analysis of the melanosome morphology in both extant and fossil fishes. These results are complemented by an interesting discussion in which mostly the optical properties (though not exclusively) are discussed, based on published literature.
I regard these results as meaningful and important for the scientific community.
However, I fear the presentation of the the statistical outcomes (in the Results section) may not be entirely accessible to the broad and interdisciplinary readership of Nature Communications. The manuscript may be a stronger fit for a more specialized journal in zoology or evolutionary biology.
It may also be regretted that no optical measurement (e.g., light scattering, or absorption) is presented in this manuscript (if none was performed at the moment that this research was carried out, I understand that it is too late to ask for this at this stage). This could have supported the discussion rather than relying solely on literature and have made the conclusions stronger.
Specific comments:
- It could be fair to mention “fish” or “aquatic environment” in the title. As it is now, I find it broad and could possibly mislead some readers.
- For some readers, it could be useful to comment in the manuscript whether the investigated melanosomes are eumelanosomes and pheomelanosomes, as both have been reported in fish [see e.g., 10.1038/ng.3098], since this may impact on morphology.
- Similarly, the authors could comment in their manuscript on the influence of factors such temperature, pH, ROS and generally bleaching on the morphology and optical properties of melanosomes [see e.g., 10.3389/fonc.2022.887770, 10.3390/ijms22158273, 10.3390/opt4040041, 10.1364/BOE.8.003966] (in addition to lines 214-217, 220-226, 239-250), as it may influence some of the discussion and conclusions.
- In lines 262-264: “this [...] suggests that melanosomes modulate light absorbance and scattering”. I am not sure why such caution oratory here. Melanosomes do influence light absorption and scattering in general [e.g., 10.1364/BOE.8.003966].
- Apart from the subject and the last complement in sentence 423-426, paragraphs 411-418 and 419-427 are the same.
- Long space should be deleted in line 431.

Reviewer #2

(Remarks to the Author)
In this study, the authors analyze the geometric characteristics of melanosomes from both extant and fossil fishes. Their approach is built on three complementary comparisons, each designed to test how melanosome morphology varies across evolutionary lineages (deep time), tissue types, and potential functional roles (see below).
The dataset comprises melanosomes sampled across major extant and fossil fish lineages and tissue types. For extant taxa, the authors include representatives of the four principal classes of fishes—Agnatha (1 species), Chondrichthyes (3 species), Actinopterygii (5 species), and Sarcopterygii (1 species). They further distinguish melanosomes from internal tissues (kidney, spleen, gut, and others) versus light-exposed tissues (skin and eye, the latter discussed in greater detail). The fossil dataset consists of ocular melanosomes from four fossil specimens corresponding to the same major extant groups, plus Acanthodii, a stem lineage of Chondrichthyes. This design allows the authors to test whether melanosomes preserve taxon-specific geometric signatures and whether these signatures are stable enough to support phylogenetic inference

through deep time.
While the study is original, important for the scientific community, extensive and well executed, several issues require clarification or revision
The authors accurately arrive to two main conclusions:
1) - By comparing extant and extinct taxa, they demonstrate that phylogenetic trends in melanosome geometry in fishes have deep evolutionary origins, established more than 306 MYA.
2) Comparative analysis of tissue-specific melanosomes indicates strong phylogenetic structuring. In general, internal melanosomes exhibit a spheroidal shape, which is consistent with their functional role in metal sequestration and scavenging of harmful reactive oxygen species (ROS). In contrast, "light-exposed melanosomes" (cutaneous or ocular tissues) also include a cylindrical population. This is particularly important in ocular melanosomes from actinopterygians and sarcopterygians.
The conclusions regarding functional differences, while discussed at length, are insufficiently supported by experimental approaches and therefore remain largely speculative.
Differences in ocular melanosome structure led the authors to hypothesize that "they reflect group-specific visual adaptations." However, these proposed adaptations are extensively discussed but not demonstrated. Essentially, the authors suggest that a cylindrical melanosome subpopulation—associated with efficient retinomotor movements—is characteristic of actinopterygians and sarcopterygians, whereas predominantly spheroidal ocular melanosomes correlate with a pupillary light reflex, as commonly observed in agnathans and chondrichthyans. Although this interpretation relies heavily on a zebrafish model in which PMEL was knocked down using morpholinos (Burgoyne et al., 2015; Ref. 8), it remains speculative in the absence of broader functional or experimental evidence.

Other comments
1) Abstract, line 23: "Unusually large and abundant melanosomes in extant lobe-finned fishes reflect physiological adaptations that underpin the vertebrate transition from water to land." Since only one extant species of lungfish was analyzed (out of six possible species, four of which belong to the same genus -African lungfish- Protopterus), this statement is an overinterpretation. Melanosome characteristics in additional lungfish species or tetrapods are neither analyzed nor discussed in the manuscript.

2) The existence of cylindrical and spheroidal melanosomes has been described in both extant species (Liu et al., 2005; Ref. 43) as well as in fossil eyes, where their presence has been suggested to be informative for phylogenetic analysis. Indeed, Clements et al. (Nature, 2016) noted: "Our analysis indicates that in addition to evidence of colour patterning, ecology and thermoregulation, fossil melanosomes can also carry a phylogenetic signal. Identification in Tullimonstrum of spheroidal and cylindrical melanosomes forming the remains of retinal pigment epithelium indicates that it is a vertebrate." This reference is omitted in the present manuscript.

3) - Discussion, line 257: Melanosome geometry in the skin of extant fishes.
The data presented show that skin melanosomes are more elongate in Sarcopterygii and Actinopterygii than in Chondrichthyes and Agnatha. The two primary roles of skin melanophores in species with light-exposed integument are colour change/reflectance for UV protection and background adaptation. To the best of my knowledge, lampreys are not capable of background adaptation. Could the authors comment on whether melanosome morphology in these taxa is expected to regulate either of these functions, and how the observed differences relate to known physiological capabilities?

4) The two discussion paragraphs in lines 411 and 419 are essentially the same paragraph, repeated with only minimal wording changes.

Reviewer #3

(Remarks to the Author)
I'm glad that I had the opportunity to review this report and learn so much about the subject, which is of great interest to me. I strongly believe it provides invaluable information about the evolution of the vertebrate visual system, which is something that I'm also curious about and working on in a related area. As a result, I am certain that the acceptance of this paper will attract the attention of scientists from other related research areas.

The manuscript is well written, the methods used are widely adopted and were apparently performed well. The results are sound, and the discussions are well presented, which led to a fine conclusion. In fact, I admit that I have very few, but genuine, comments to be made on this study. Anyhow, the authors deserve all the congratulations for the good work they have done. I think this report fills all the requirements of Nature Communications and should be published without many significant changes.

Cheers,

Gustavo Prado

REVIEW

Line 87: Curious that melanosomes were found in the gills of several species. This organ is generally filled with erythrocytes and rarely with melanin [1]. The presence of melanosomes in gills as well as in several organs of fish is generally

associated with environmental stressors, such as waterborne insults (e.g., surfactants and heavy metal pollution), microbial and parasitic infections, and high salinity, all of which point to a general health problem [2-5]. Furthermore, melanosomes in gills are generally linked with melanomacrophage centres (MMCs) [6]. These MMCs are formed by the accumulation of macrophage cells rich in melanosomes; however, they can also be rich in lipofuscins (deposits of lipids), and siderosomes (an organelle with the accumulation of effete red blood cells or haemosiderin) [6-7]. Because these cells are not able to digest melanin, their size increases to several microns whilst transported together with other immune cells to the damaged tissue [8].
References:
[1] Daskalov, H., Robertson, P.A., & Austin, B. (2000). Influence of oxidized lipids in diets on the development of rainbow trout fry syndrome. Journal of Fish Diseases, 23, 7-14. DOI: 10.1046/j.1365-2761.2000.00215.x
[2] Mallatt, J. (1985). Fish gill structural changes induced by toxicants and other irritants: A statistical review. Canadian Journal of Fisheries and Aquatic Sciences, 42, 630-648. DOI: 10.1139/f85-083
[3] Dane, H., & Şişman, T. (2015). Histopathological changes in gill and liver of Capoeta capoeta living in the Karasu River, Erzurum. Environmental Toxicology, 30, 904-917. DOI: 10.1002/tox.21965
[4] Baiomy, A.A. (2016). Histopathological biomarkers and genotoxicity in gill and liver tissues of Nile tilapia Oreochromis niloticus from a polluted part of the Nile River, Egypt. African Journal of Aquatic Science, 41, 181-191. DOI: 10.2989/16085914.2016.1168734
[5] Pagenkopf, G.K. (1983). Gill surface interaction model for trace-metal toxicity to fishes: Role of complexation, pH, and water hardness. Environmental Science & Technology, 17, 342-347. DOI: 10.1021/es00112a007
[6] Wolke, R.E. (1992). Piscine macrophage aggregates: A review. Annual Review of Fish Diseases, 2, 91-108. DOI: 10.1016/0959-8030(92)90058-6
[7] Roberts, R.J. (1975). Melanin-containing cells of teleost fish and their relation to disease. In: Ribelin, W.E. (org.). (1975). The pathology of fishes. The University of Wisconsin Press, 1st Ed., pp. 399-428
[8] Agius, C.,& Roberts, R.J. (2003). Melano-macrophage centres and their role in fish pathology. Journal of Fish Diseases, 26, 499-509. DOI: 10.1046/j.13652761.2003.00485.x

Lines 168-179: I don't get why the authors used the MM if their dataset clearly shows a difference in the geometry of ocular melanosomes. Isn't the BC sufficient to demonstrate it? Though I think that perhaps this method was performed to provide further support to previous interpretations. Anyway, the results are great, as expected!

Lines 252-253: Besides Mazon Creek, to which fossils does this phrase apply? I mean, did you include specimens from other deposits? Are all sarcopterygians preserved only in estuarine sediments?

Lines 262-265: Yeah, I might be wrong, but isn't this widely acknowledged? Perhaps rephrasing to something like "your results give further support to this interpretation" would be better than what is written.

Lines 277-279: Just to complement, melanosomes at the RPE Tapetum are mostly oval [1-3], occasionally spherical [1], but rarely elongated (or spindle-shaped [4]. Sure, this trait vary according to species and habitat, but this trend is seen in most vertebrates, including fishes.
References:
[1] Arnott, H.J., Maciolek, N.J., & Nicol, J.A.C. (1970). Retinal Tapetum lucidum: A novel reflecting system in the eye of teleosts. Science, 169, 478-480. DOI: 10.1126/science.169.3944.478
[2] Zueva, L., Tsytsarev, V., Alves, J., & Inyushin, M. (2024). Melanin in the Retinal Epithelium and Magnetic Sensing: A Review of Current Studies. Biophysica, 4, 466-476. DOI: 10.3390/biophysica4040030
[3] Braekevelt, C.R., & Thorlakson, I.J. (1993). Histology and Histopathology Fine structure of the retinal pigment epithelium of the great horned owl (Bubo virginianus). Histology and Histopathology, 8, 17-23. PMID: 8443429
[4] Braekvelt, C.R. (1980). Fine Structure of the Retinal Epithelium and Tapetum Lucidum in the Giant Danio (Danio malabaricus) (Teleost). Anatomy and Embryology, 158, 317-328. DOI: 10.1007/BF00301820

Lines 362-365: This is a very intriguing subject!! Better yet, it would be nice to test if they could be older by analysing the (four) eyes of early vertebrates. Cool finding!

Line 375: Perhaps it was just nature experimenting with different approaches. It's not surprising that these animals became extinct...

Lines 380-381: Not necessarily. What about the hypothesis of melanosome alteration through ageing? Some studies have demonstrated that, at least in humans, eye melanosomes shrink with age. This is because new melanosomes are not synthesised de novo, since eye melanosomes are created only during embryonic development [1-3]. Therefore, the question that arises would be relative to the age of the fish sampled. Aside from that, just food for thought, one should consider that melanosome size and density diminish in some regions of the RPE [4]. Consequently, the apparent reduction of microbody size in fossils (from the Mazon Creek) could be biased as a direct result of this sampled area.
References:
[1] Nagase, S., Kajiura, Y., Mamada, A., Abe, H., Shibuichi, S., Satoh, N., Itou, T., Shinohara, Y. and Amemiya, Y. (2010), Changes in structure and geometric properties of human hair by aging. International Journal of Cosmetic Science, 32, 314. DOI: 10.1111/j.1468-2494.2010.00580_5.x
[2] Itou, T. (2018). Morphological changes in hair melanosomes by aging. Pigment Cell and Melanoma Research, 31, 630-635. DOI: 10.1111/pcmr.12697
[3] Itou, T., Ito, S., & Wakamatsu, K. (2019). Effects of Aging on Hair Color, Melanosome Morphology, and Melanin Composition in Japanese Female. International Journal of Molecular Sciences, 20, 3739. DOI: 10.3390/ijms20153739

[4] Schmidt, S.Y., & Peisch, R.D. (1986). Melanin concentration in normal human retinal pigment epithelium. Regional variation and age-related reduction. Investigative Ophthalmology & Visual Science, 27, 1063-1070. PMID: 3721785

Line 384: Albeit not wrong, I feel this excerpt is a little bolder because vertebrate visual systems imply the preservation of other structures, such as photoreceptors and ganglia. However, it is undeniable that the importance of this study lies in the strong evidence it provides for eye melanisation (and its relative properties) among fish.

Lines 411-417: This paragraph is repeated, though the next one shows more information.

Line 431: Delete the space.

Lines 433-434: Trying to understand why a group of animals doesn't use a particular evolutionary strategy is misguided, as it would imply an idea of directed evolution. In other words, things in nature happen arbitrarily, without purpose or direction. Therefore, trying to understand is merely a mental exercise.

Lines 444-445: What about dwelling in shallow bodies of water?

Lines 448-452: Just to add. What about the hypothesis of signalling among these animals or sexual selection? I guess that the enhancement of the visual system would favour chondrichthyes to communicate or better identify sexual partners [1]. Taking into consideration these aspects could also modulate the evolution of visual traits (in addition to increasing diversity of melanosomes) without requiring the explanations of constant changing of light levels.
References:
[1] Gayford, J.H. (2023). The evolution of sexual dimorphism in Chondrichthyes: drivers, uncertainties, and future directions. Environmental Biology of Fishes, 106, 1463–1475. DOI: 10.1007/s10641-023-01425-x

Lines 690-692: Do all dark regions preserve melanosomes?

Lines 693-694: Just out of curiosity, were the fossils examined in their entirety? I mean, were they placed directly under a SEM? Why not collect samples by extracting small fragments (<1 mm)?

Line 822: Has this study been approved by an ethics committee? If yes, then it would be nice to put in brackets the number of the protocol (if it exists).

Version 1:

Reviewer comments:

Reviewer #1

(Remarks to the Author)
The authors rigorously addressed all my comments. I congratulate them on their good results and nice manuscript.
I am looking forward reading their ms in prep addressing the question about eumelanosomes and phaeomelanosomes in fish, when it is published.

Reviewer #2

(Remarks to the Author)
I appreciate the effort and dedication the authors invested in this new version, which substantially improves the previous manuscript. Regarding the main concerns raised by the Reviewer, the authors conducted additional histological and SEM analyses that provide stronger support for their proposed functional interpretations. Textual statements have been moderated to avoid over interpretation. Importantly, the authors also clarified the PMEL knockout experiments, adding new explanations directly into the manuscript. Other minor concerns were properly addressed through targeted modifications in the text.

Reviewer #3

(Remarks to the Author)
I have nothing new to say about the manuscript, I only have positive view about it. The MS is great and now can be published since the authors have performed all changes I suggested or considered my comments.

Open Access This Peer Review File is licensed under a Creative Commons Attribution 4.0 International License, which permits use, sharing, adaptation, distribution and reproduction in any medium or format, as long as you give appropriate credit to the original author(s) and the source, provide a link to the Creative Commons license, and indicate if changes were made.
In cases where reviewers are anonymous, credit should be given to 'Anonymous Referee' and the source.
The images or other third party material in this Peer Review File are included in the article's Creative Commons license, unless indicated otherwise in a credit line to the material. If material is not included in the article's Creative Commons license and your intended use is not permitted by statutory regulation or exceeds the permitted use, you will need to obtain permission directly from the copyright holder.
To view a copy of this license, visit https://creativecommons.org/licenses/by/4.0/

Response to referees' comments

Handling editor

Comment 1. The reviewers raise important concerns. We find that these concerns limit the strength of the study, and therefore we ask you to address them with additional work.

Response: We have addressed all of the reviewers' concerns by incorporating changes to the text, figures, tables and extended data. We have also conducted additional analyses as requested. We believe that these changes have substantially improved the manuscript and the strength of our interpretations, and we trust that they satisfy the reviewers' requests.

Reviewer 1

Comment 1: "The submitted manuscript reports on an impressive dataset and a robust statistical analysis of the melanosome morphology in both extant and fossil fishes. These results are complemented by an interesting discussion in which mostly the optical properties (though not exclusively) are discussed, based on published literature. I regard these results as meaningful and important for the scientific community."
Response: We thank the reviewer for his/her positive comment on the importance of our paper and on our analytical rigour.

Comment 2: I fear the presentation of the statistical outcomes (in the Results section) may not be entirely accessible to the broad and interdisciplinary readership of Nature Communications. The manuscript may be a stronger fit for a more specialized journal in zoology or evolutionary biology.
Response: The reviewer does not provide specific line numbers in this comment; the original manuscript included the results of statistical tests throughout much of the Results section, from L126 – 248. We have modified the text throughout the Results section (L81 – 247) to improve readability and accessibility to a broad audience, as follows: (1) Mid-sentence references to statistical values have been moved to the ends of sentences where possible. (2) Additional references to figures and tables have been included to better guide the reader to the appropriate data. (3) Text relating to mixture modelling has been revised for clarity (L196–248 of the revised manuscript). (4) A brief synopsis has been added to the results to state the approaches used and explain their purpose (L118–124, L196–200). (5) Additional histological work has been included to better summarise and visualise key findings (L102–115, Extended Data Figs. 3–5 and 14).

In addition, we have moved the density histogram originally provided in Extended Data Fig. 4 to Fig. 4 in the main manuscript. This figure provides a clear visual representation of the bimodal melanosome distributions in various phylogenetic groups; it allows the reader to quickly identify key differences in melanosome geometry without any prior understanding of mixture model analysis.

Comment 3: It may also be regretted that no optical measurement (e.g., light scattering, or absorption) is presented in this manuscript (if none was performed at the moment that this research was carried out, I understand that it is too late to ask for this at this stage). This could have supported the discussion rather than relying solely on literature and have made the conclusions stronger.

Response: This is an interesting point. While direct optical measurements can be useful in certain contexts, they are neither appropriate nor fully applicable to our dataset and come with assumptions and uncertainties that we would prefer to avoid introducing to our study.

(1) Direct measurements to quantify optical performance at the cellular level are normally applied to small-scale, targeted experimental systems, and light scattering can be quite complex, incorporating forward-scatter, backward scatter, narrow-angle and straylight components. As such, this falls beyond the scope of our large-scale comparative study. The aim of our study is to resolve broad evolutionary patterns in melanosome geometry across tissues and vertebrate lineages, including fossil taxa, for which direct optical characterisation is not feasible.

(2) Current understanding of the relationship between melanosome geometry and optical function is based largely on rigorous modelling and targeted experimental studies of specific tissues (e.g., retinal pigment epithelium). Collectively, this research supports the general principle that melanosome shape and organisation influence interactions with light [1–3]. The interpretations presented here are grounded within this well-established framework.

(3) The optical properties of melanosomes are unlikely to be determined solely by melanosome geometry. Chemical composition, including variation in eumelanin–pheomelanin ratios, monomer structure, and degree of polymerisation, may also contribute to absorption and scattering [4]. However, these chemical data are not currently known for fish and cannot be fully assessed in fossils. Consequently, the relative contributions of melanosome chemistry and geometry to optical behaviour remain unresolved at macro-evolutionary scales and likely require integrated experimental approaches beyond the scope of this study. Linking optical properties to melanosome geometry is therefore somewhat premature.

Our work provides an exhaustive dataset on melanosome geometry across fish tissues and taxa that will act as a critical foundation for future work, enabling future systematic assessment of the link between melanosome optical properties and geometry, and chemistry at different scales spanning the monomer unit, oligomer stacking and gross polymer organization.

Notwithstanding these issues, we appreciate the reviewer's suggestion that additional data could support our discussion. The revised manuscript therefore includes additional support for the discussion and conclusions in the form of new data on eye structure from SEM and histology (Extended Data Figs. 3, 5, 6 and 14).

[1] Song, W., Zhang, L., Ness, S. & Yi, J. Wavelength-dependent optical properties of melanosomes in retinal pigmented epithelium and their changes with melanin bleaching: a numerical study. Biomed. Opt. Express 8, 3966–3980 (2017). https://doi.org/10.1364/BOE.8.003966
[2] Davis, A. L. et al. Ultra-black camouflage in deep-sea fishes. Curr. Biol. 30, 3470–3476 (2020). https://doi.org/10.1016/j.cub.2020.06.044
[3] Kim, I. T. & Choi, J. B. Melanosomes of retinal pigment epithelium–distribution, shape, and acid phosphatase activity. Korean J. Ophthalmol. 12, 85–91 (1998). https://doi.org/https://doi.org/10.3341/kjo.1998.12.2.85
[4] Micillo, R. et al. "Fifty Shades" of black and red or how carboxyl groups fine tune eumelanin and pheomelanin properties. Int. J. Mol. Sci. 17, 746 (2016). https://doi.org/10.3390/ijms17050746

Comment 4: It could be fair to mention “fish” or “aquatic environment” in the title. As it is now, I find it broad and could possibly mislead some readers.
Response: Title has been modified as requested.

Comment 4: For some readers, it could be useful to comment in the manuscript whether the investigated melanosomes are eumelanosomes and pheomelanosomes, as both have been reported in fish [see e.g., 10.1038/ng.3098], since this may impact on morphology.
Response: The reviewer touches on a very interesting point here. The presence of pheomelanin in fish is currently debated and the evidence presented in the cited study has been questioned [1]. To date, direct chemical assay of fish tissues has identified only eumelanin [2 and 3].

The terms *eumelanosome* and *phaeomelanosome* were originally coined in studies of melanosomes in feathers in the 1970s [4] and since then have been applied widely in studies of feathers and mammal skin/ hair, where there is a broad relationship between melanosome geometry and visible of the tissue colour (rufous-yellow *pheomelanosomes* are spherical while and black-brown *eumelanosomes* are prolate spheroids to cylindrical). To date, there is no evidence that this relationship applies to other tissues and taxa. Indeed, work on the skin of reptiles and birds reported no link between melanosome shape and visible colour [5]. Previous work by some members of this author team showed that melanosomes of disparate shapes in internal tissues and skin in amphibians, reptiles, birds and mammals all have a mixed eumelanin-phaeomelanin composition [6]. Further, two recent studies have called into question the link between melanosome shape and feather colour [7 and 8]. Clearly this issue is far from resolved.

The authors currently have another paper in preparation that specifically addresses this question, by combining the data on melanosome geometry (from the current manuscript) with a new and exhaustive dataset on melanin monomer chemistry in fish. Therefore, it is prudent to avoid statements regarding *eumelanosomes* and *phaeomelanosomes* in fish until our follow-up paper is published. The dark colouration of melanosome extracts is suggestive that all melanosomes reported reflect

predominantly eumelanin-rich melanosomes. Text has been added to the Results section to address this point as requested (L82–83 and L256–261).

[1] Kottler, V. A., Kunstner, A. & Schartl, M. Pheomelanin in fish? Pigment Cell Melanoma Res. 28, 355–356 (2015). https://doi.org/10.1111/pcmr.12359
[2] Adachi, K. et al. The histological analysis, colorimetric evaluation, and chemical quantification of melanin content in 'suntanned' fish. Pigment Cell Res. 18, 465–468 (2005). https://doi.org/10.1111/j.1600-0749.2005.00272.x
[3] Adachi, K. et al. A close relationship between androgen levels and eumelanogenesis in the teleost red seabream (Pagrus major): Quantitative analysis of its seasonal variation and effects of oral treatment with methyl-testosterone. Comp. Biochem. Physiol. A Mol. Integr. Physiol. 156, 184–189 (2010). https://doi.org/10.1016/j.cbpa.2010.01.021
[4] Brumbaugh, J. A. The ultrastructural effects of the I and S loci upon black-red melanin differentiation in the fowl. Dev. Biol. 24, 392–412 (1971). https://doi.org/10.1016/0012-1606(71)90088-1
[5] Li, Q. et al. Melanosome evolution indicates a key physiological shift within feathered dinosaurs. Nature 507, 350–353 (2014). https://doi.org/10.1038/nature12973
[6] Rossi, V., McNamara, M. E., Webb, S. M., Ito, S. & Wakamatsu, K. Tissue-specific geometry and chemistry of modern and fossilized melanosomes reveal internal anatomy of extinct vertebrates. Proc. Natl. Acad. Sci. U.S.A. 116, 17880–17889 (2019). https://doi.org/10.1073/pnas.1820285116
[7] Norden, K. K., Cooney, C. R., Babarovic, F. & Stoddard, M. C. Drab cuckoos retain the melanosome types of brilliantly coloured relatives. J. R. Soc. Interface 23, 20251024 (2026). https://doi.org/10.1098/rsif.2025.1024
[8] Sutter, S. M. et al. A multiscale physicochemical characterization of melanin-based feather colouration in the rapid capuchino seedeater radiation. R. Soc. Open Sci. 13 (2026). https://doi.org/10.1098/rsos.252505

Comment 5: Similarly, the authors could comment in their manuscript on the influence of factors such temperature, pH, ROS and generally bleaching on the morphology and optical properties of melanosomes, as it may influence some of the discussion and conclusions. (in addition to lines 214-217, 220-226, 239-250)
[1] 10.3389/fonc.2022.887770,
[2] 10.3390/ijms22158273,
[3] 10.3390/opt404004,
[4] 10.1364/BOE.8.003966
Response: Although pH can influence melanin synthesis and melanosome morphology, these effects occur intravascularly during melanosome biogenesis rather than as post-depositional modification [1,2]. Regulation is primarily governed by intramelanosomal pH and is therefore not directly driven by external environmental pH. To our knowledge, changes in melanosome geometry resulting from environmental changes in pH have not been reported.

Temperature has been reported to influence melanin production and distribution in some systems [3–4]; however, we are currently not aware of any evidence for temperature-dependent variation in melanosome geometry. Experimental taphonomic

studies indicate that post-depositional shrinkage of melanosomes may occur under elevated temperatures [5], which should be considered when interpreting fossil material.

Text and relevant citations have been included to discuss these additional factors (L327–333 and 455–468).

[1] You J, Yusupova M and Zippin JH (2022) The potential impact of melanosomal pH and metabolism on melanoma. Front. Oncol. 12:887770. doi: 10.3389/fonc.2022.887770
[2] Koirala M, Shashikala HBM, Jeffries J, Wu B, Loftus SK, Zippin JH, Alexov E. Computational Investigation of the pH Dependence of Stability of Melanosome Proteins: Implication for Melanosome formation and Disease. International Journal of Molecular Sciences. 2021; 22(15):8273. https://doi.org/10.3390/ijms22158273
[3] Barni, S. et al. Increase in liver pigmentation during natural hibernation in some amphibians. J. Anat. 195, 19–25 (1999). https://doi.org/10.1046/j.1469-7580.1999.19510019.x
[4] McNamara, M. E., Briggs, D. E., Orr, P. J., Field, D. J. & Wang, Z. Experimental maturation of feathers: implications for reconstructions of fossil feather colour. Biol. Lett. 9, 20130184 (2013). https://doi.org/10.1098/rsbl.2013.0184

Comment 6: In lines 262-264: "this [...] suggests that melanosomes modulate light absorbance and scattering". I am not sure why such caution oratory here. Melanosomes do influence light absorption and scattering in general [e.g., 10.1364/BOE.8.003966].
Response: It is widely known that melanosomes absorb and scatter light. In fish, however, these properties have been associated with melanosome attributes (e.g. size/shape/packing density) only for deep sea species [1]. We have amended the text to clarify (L319–322).

[1] Davis, A. L. et al. Ultra-black camouflage in deep-sea fishes. Curr. Biol. 30, 3470–3476 (2020). https://doi.org/10.1016/j.cub.2020.06.044

Comment 7: Apart from the subject and the last complement in sentence 423-426, paragraphs 411-418 and 419-427 are the same.
Response: Apologies for the confusion. Repeated text has been deleted.

Comment 8: Long space should be deleted in line 431.
Response: Space deleted, along with other double spaces identified in the manuscript.

Reviewer 2
Comment 1: "While the study is original, important for the scientific community, extensive and well executed, several issues require clarification or revision".
Response: We thank the reviewer for his/her positive comment on the quality and importance of our study. We have addressed all of the issues raised by incorporating appropriate clarifications and revisions of the text and figures.

Comment 2: The conclusions regarding functional differences, while discussed at length, are insufficiently supported by experimental approaches and therefore remain largely speculative. Differences in ocular melanosome structure led the authors to hypothesize that "they reflect group-specific visual adaptations." However, these proposed adaptations are extensively discussed but not demonstrated. Essentially, the authors suggest that a cylindrical melanosome subpopulation—associated with efficient retinomotor movements—is characteristic of actinopterygians and sarcopterygians, whereas predominantly spheroidal ocular melanosomes correlate with a pupillary light reflex, as commonly observed in agnathans and chondrichthyans. Although this interpretation relies heavily on a zebrafish model in which PMEL was knocked down using morpholinos (Burgoyne et al., 2015; Ref. 8), it remains speculative in the absence of broader functional or experimental evidence.
Response: We thank the reviewer for raising this point. We note that the reviewer does not dispute our main results, which are that eye melanosome geometry holds a strong phylogenetic signal, but rather the functional implications of the differences in (eye melanosome) geometry among major fish clades.

As suggested by the reviewer, the revised manuscript includes additional support for our interpretations in the form of new experimental evidence and broader evidence from across vertebrates on ocular function.

(1) We have conducted additional analyses of the eyes of fish species from all four major phylogenetic groups cited above. Our new histological and SEM data demonstrate marked differences in RPE architecture among these groups. In Osteichthyes (bony fishes), cylindrical melanosomes are present in the retinal pigment epithelium (RPE), where they are oriented parallel to the photoreceptors (Extended Data Figs. 4–6). In contrast, cylindrical melanosomes are absent in chondrichthyans and agnathans. Notably, melanosomes are substantially reduced/absent within the RPE of Chondrichthyes. In all groups, spheroidal melanosomes occur in the choroid and iris, but in Chondrichthyes and Sarcopterygii they are typically stratified into size-specific layers, with larger melanosomes on the vitreal (inner-facing) side (Extended Data Figs. 4–6). This agrees with previous studies of Chondrichthyes and Sarcopterygii [1–4]. These critical new data on eye architecture directly support our interpretations of functional divergence in ocular light regulatory organisation (L102–115).

(2) We did not mean to imply that PMEL knockdown experiments in zebrafish are the primary basis for our interpretations. Instead, these experiments are integrated with established differences in ocular physiology and morphology across vertebrate lineages more broadly, spanning mammals, urodeles, reptiles, anurans, birds and fishes [5–7] (L359 – 407). The revised manuscript provides additional text to better clarify these broad and established patterns (L372– 376, L384–392, L399–406). Our data clearly show that in fish, cylindrical melanosomes are associated with RPE systems exhibiting RMMs, and that ocular systems relying on PLR and/or choroidal melanosome movements show only spheroidal melanosomes, and in Chondrichthyes, reduction / loss of melanosomes in RPE cells (Supplementary Figs. 4 and 6).

3) In defence of our choice of zebrafish (a teleost) as our model system: teleosts show the most diverse RMMs known and thus provide a particularly well-resolved system for investigating mechanisms linking melanosome morphology to dynamic light regulation in the RPE.

4) Finally, PMEL downregulation during pheomelanin synthesis is widely understood across vertebrates and results in the characteristic spheroidal geometry of pheomelanin-rich melanosomes [8, 9] and (in mice) the loss of elongate melanosomes [10]. Thus, it is widely accepted that PMEL downregulation results in a loss of cylindrical melanosomes across vertebrates, not just in zebrafish [11–12].

Therefore, our interpretations are based on (1) our new data on melanosome geometry, (2) our new data on eye ultrastructure, (3) previous comparative data on RMMs across vertebrates, (4) previous data on how PMEL impacts melanosome geometry across vertebrates, and (5) established experimental evidence from model systems. Text and references have been added throughout the manuscript to provide context on the above, and strengthen the arguments presented.

[1] Schieber, N. L., Collin, S. P. & Hart, N. S. Comparative retinal anatomy in four species of elasmobranch. J. Morphol. 273, 423–440 (2012). https://doi.org/10.1002/jmor.11033
[2] Litherland, L., Collin, S. P. & Fritsches, K. A. Visual optics and ecomorphology of the growing shark eye: a comparison between deep and shallow water species. J. Exp. Biol. 212, 3583–3594 (2009). https://doi.org/10.1242/jeb.028589
[3] Theiss, S. M., Collin, S. P. & Hart, N. S. Interspecific visual adaptations among wobbegong sharks (Orectolobidae). Brain Behav. Evol. 76, 248–260 (2010). https://doi.org/10.1159/000321330
[4] Collin, H. B., Ratcliffe, J. & Collin, S. P. Morphology of the cornea and iris in the Australian lungfish Neoceratodus forsteri (Krefft 1870) (Dipnoi): Functional and evolutionary perspectives of transitioning from an aquatic to a terrestrial environment. J. Morphol. 285, e21662 (2024). https://doi.org/10.1002/jmor.21662
[5] Douglas, R. H. The pupillary light responses of animals; a review of their distribution, dynamics, mechanisms and functions. Prog. Retin. Eye Res. 66, 17–48 (2018). https://doi.org/10.1016/j.preteyeres.2018.04.005
[6] Burnside, B. & King-Smith, C. Fish Retinomotor Movements. in Encyclopedia of the eye (ed A. D. Dartt) 142–150 (Academic Press, 2010).
[7] Kim, I. T. & Choi, J. B. Melanosomes of retinal pigment epithelium–distribution, shape, and acid phosphatase activity. Korean J. Ophthalmol. 12, 85–91 (1998). https://doi.org/https://doi.org/10.3341/kjo.1998.12.2.85
[8] Kobayashi, T. et al. Modulation of melanogenic protein expression during the switch from eu-to pheomelanogenesis. (1995). https://doi.org/10.1242/jcs.108.6.2301
[9] Ito, S. & Jimbow, K. Quantitative analysis of eumelanin and pheomelanin in hair and melanomas. J. Invest. Dermatol. 80, 268–272 (1983). https://doi.org/10.1111/1523-1747.ep12534616
[10] Hellstrom, A. R. et al. Inactivation of Pmel alters melanosome shape but has only a subtle effect on visible pigmentation. PLoS Genet. 7, e1002285 (2011). https://doi.org/10.1371/journal.pgen.1002285

[11] D'Alba, L. & Shawkey, M. D. Melanosomes: Biogenesis, properties, and evolution of an ancient organelle. Physiol. Rev. 99, 1–19 (2019). https://doi.org/10.1152/physrev.00059.2017
[12] Raposo, G. & Marks, M. S. Melanosomes—dark organelles enlighten endosomal membrane transport. Nat. Rev. Mol. Cell Biol. 8, 786–797 (2007). https://doi.org/10.1038/nrm2258

Comment 3: Abstract, line 23: "Unusually large and abundant melanosomes in extant lobe-finned fishes reflect physiological adaptations that underpin the vertebrate transition from water to land." Since only one extant species of lungfish was analysed (out of six possible species, four of which belong to the same genus -African lungfish- *Protopterus*), this statement is an overinterpretation. Melanosome characteristics in additional lungfish species or tetrapods are neither analysed nor discussed in the manuscript.

Response: We agree that a broader comparative context from additional lungfish and tetrapods is important. Widespread melanisation has also been reported in the African Lungfish *Protopterus aethiopicus* [1], and this work is now cited in the revised manuscript (L277–278).

Additionally, we have expanded the discussion to include relevant evidence from amphibians and other tetrapods where internal melanins have been implicated in physiological and ecological adaptations to terrestrial environments [2–4]. This additional text provides a richer comparative framework for interpreting the potential functional implications of melanosome abundance in the context of vertebrate terrestrialisation (L298–305).

In addition, we have toned down the language to avoid overinterpretation of our data (L20–22).

[1] Agius, C. Phylogenetic development of melano-macrophage centres in fish. J. Zool. 191, 11–31 (1980). https://doi.org/https://doi.org/10.1111/j.1469-7998.1980.tb01446.x
[2] Franco-Belussi, L., Provete, D. B. & De Oliveira, C. Environmental correlates of internal coloration in frogs vary throughout space and lineages. Ecol. Evol. 7, 9222–9233 (2017). https://doi.org/10.1002/ece3.3438
[3] Barni, S. et al. Increase in liver pigmentation during natural hibernation in some amphibians. J. Anat. 195, 19–25 (1999). https://doi.org/10.1046/j.1469-7580.1999.19510019.x
[4] Moore, V. D. G. & Haenel, G. J. Variation in melanin content of lizard livers: Hybrids turning to the dark side. Physiol. Biochem. Zool. 95, 536–543 (2022). https://doi.org/10.1086/721445

Comment 4: The existence of cylindrical and spheroidal melanosomes has been described in both extant species (Liu et al., 2005; Ref. 43) as well as in fossil eyes, where their presence has been suggested to be informative for phylogenetic analysis. Indeed, Clements et al. (Nature, 2016) noted: "Our analysis indicates that in addition to evidence of colour patterning, ecology and thermoregulation, fossil melanosomes can

also carry a phylogenetic signal. Identification in Tullimonstrum of spheroidal and cylindrical melanosomes forming the remains of retinal pigment epithelium indicates that it is a vertebrate." This reference is omitted in the present manuscript.
Response: Agreed. Text has been amended and the suggested reference has been included (L471–475).

Comment 5: Discussion, line 257: Melanosome geometry in the skin of extant fishes. The data presented show that skin melanosomes are more elongate in Sarcopterygii and Actinopterygii than in Chondrichthyes and Agnatha. The two primary roles of skin melanophores in species with light-exposed integument are colour change/reflectance for UV protection and background adaptation. To the best of my knowledge, lampreys are not capable of background adaptation. Could the authors comment on whether melanosome morphology in these taxa is expected to regulate either of these functions, and how the observed differences relate to known physiological capabilities?
Response: These are really good questions. To our knowledge, there has not been a comprehensive study of physiological colour change in non-teleost fish, and it's not clear if melanosome migration in melanophore processes is subject to the same physical constraints as in RPE cells. Further work here is needed, but we have amended the text to include brief discussion of this point (L322–325).

Comment 6: The two discussion paragraphs in lines 411 and 419 are essentially the same paragraph, repeated with only minimal wording changes.
Response: Apologies for the confusion. Repeated text has been deleted.

Reviewer 3

Comment 1: "I'm glad that I had the opportunity to review this report and learn so much about the subject, which is of great interest to me. I strongly believe it provides invaluable information about the evolution of the vertebrate visual system, which is something that I'm also curious about and working on in a related area. As a result, I am certain that the acceptance of this paper will attract the attention of scientists from other related research areas. The manuscript is well written, the methods used are widely adopted and were apparently performed well. The results are sound, and the discussions are well presented, which led to a fine conclusion."
Response: We thank the reviewer for their positive comments on the quality, rigour and broad interest of our manuscript.

Comment 2: Line 87: Curious that melanosomes were found in the gills of several species. This organ is generally filled with erythrocytes and rarely with melanin [1]. The presence of melanosomes in gills as well as in several organs of fish is generally associated with environmental stressors, such as waterborne insults (e.g., surfactants and heavy metal pollution), microbial and parasitic infections, and high salinity, all of which point to a general health problem [2-5]. Furthermore, melanosomes in gills are generally linked with melanomacrophage centres (MMCs) [6]. These MMCs are formed by the accumulation of macrophage cells rich in melanosomes; however, they can also be rich in lipofuscins (deposits of lipids), and siderosomes (an organelle with the accumulation of effete red blood cells or haemosiderin) [6-7]. Because these cells are

not able to digest melanin, their size increases to several microns whilst transported together with other immune cells to the damaged tissue [8].

Refs:
[1] 10.1046/j.1365-2761.2000.00215.x
[2] 10.1139/f85-083
[3] 10.1002/tox.21965
[4] 10.2989/16085914.2016.1168734
[5] 10.1021/es00112a007
[6] 10.1016/0959-8030(92)90058-6
[7] Roberts, R.J. (1975). Melanin-containing cells of teleost fish and their relation to disease.
[8] 10.1046/j.13652761.2003.00485.x

Response: The reviewer does not request any particular changes here, but in the revised manuscript we have included a brief summary of the cellular context of melanin within internal tissues (L268–273). We have also included new histological data showing images of melanosomes within melanomacrophage centres (L102–106; Extended Data Fig. 3)

Comment 3: Lines 168-179: I don't get why the authors used the MM if their dataset clearly shows a difference in the geometry of ocular melanosomes. Isn't the BC sufficient to demonstrate it? Though I think that perhaps this method was performed to provide further support to previous interpretations. Anyway, the results are great, as expected!

Response: While the Bhattacharyya coefficient (BC) is sufficient to demonstrate that significant differences exist between groups, mixture modelling (MM) demonstrates *how* the groups differ. MM was particularly important for ocular tissues as they often include diverse melanosome geometries. In our study, the MM provided a statistical basis for assigning melanosomes of different geometries to separate populations. This, in turn, facilitated pairwise post-hoc tests for a more granular understanding of how melanosome geometry varies in the dataset.

Comment 4: Lines 252-253: Besides Mazon Creek, to which fossils does this phrase apply? I mean, did you include specimens from other deposits? Are all sarcopterygians preserved only in estuarine sediments?

Response: We used fossil specimens from Mazon Creek only, for two reasons. (1) Fossil specimens from different localities inherently differ in their taphonomic history. It is not fully known which taphonomic processes may impact melanosome geometry, but fossilization experiments by one of us (MMN) have demonstrated that melanosomes shrink during thermal maturation, where shrinkage increases with temperature. As burial temperature is unknown for many fossil deposits preserving melanosomes in fish, we decided to eliminate this variable by using specimens from a single fossil locality (and thus that have the same thermal history). (2) Mazon Creek includes specimens from each of the four extant fish groups studied in the current manuscript, which preserve soft tissues, including eyespots. [1-2]. This has now been clarified in the methods (L791–797).

Regarding the distribution of the sarcopterygians: the Mazon Creek fossils occur as two spatially separated faunal assemblages: the Essex Formation, regarded as marine; and the Braidwood Formation, regarded as estuarine. All sarcopterygian fossils included in the study were collected from Pit 11 (Will and Kankakee counties, Illinois, USA), associated with the Essex Formation [3]. Lungfish scales and teeth have also been reported from the Braidwood Formation [4]. Aquatic species in the brackish Braidwood assemblage are considered to have lived predominantly in those areas, while the presence of these species in the Essex assemblage is considered to indicate the seaward limit of their aquatic range [4]. This is now clarified in the Methods (L834–838).

[1] Colleary, C. et al. Chemical, experimental, and morphological evidence for diagenetically altered melanin in exceptionally preserved fossils. Proc. Natl. Acad. Sci. U.S.A. 112, 12592–12597 (2015). https://doi.org/10.1073/pnas.1509831112
[2] McNamara, M. E., Briggs, D. E., Orr, P. J., Field, D. J. & Wang, Z. Experimental maturation of feathers: implications for reconstructions of fossil feather colour. Biol. Lett. 9, 20130184 (2013). https://doi.org/10.1098/rsbl.2013.0184
[3] Clements, T., Purnell, M. & Gabbott, S. The Mazon Creek Lagerstätte: a diverse late Paleozoic ecosystem entombed within siderite concretions. J. Geol. Soc. 176, 1–11 (2018). https://doi.org/10.1144/jgs2018-088
[4] Schiffbauer, J. et al. 283,821 concretions, how do you measure the Mazon Creek? Assessing the paleoenvironmental and taphonomic nature of the Braidwood and Essex assemblages. Paleobiology 51, 488–506 (2025). https://doi.org/10.1017/pab.2025.10045

Comment 5: Lines 262-265: Yeah, I might be wrong, but isn't this widely acknowledged? Perhaps rephrasing to something like "your results give further support to this interpretation" would be better than what is written.
Response: It is widely known that melanins absorb and scatter light. In fish, however, these properties have been associated with melanosome attributes (e.g. size/shape/packing density) for deep sea species only [1]. We have amended the text to clarify (L319–322).

[1] Davis, A. L. et al. Ultra-black camouflage in deep-sea fishes. Curr. Biol. 30, 3470–3476 (2020). https://doi.org/10.1016/j.cub.2020.06.044

Comment 6: Lines 277-279: Just to complement, melanosomes at the RPE Tapetum are mostly oval [1-3], occasionally spherical [1], but rarely elongated (or spindle-shaped [4]. Sure, this trait vary according to species and habitat, but this trend is seen in most vertebrates, including fishes.
Refs:
[1] 10.1126/science.169.3944.478
[2] 10.3390/biophysica4040030
[3]] Braekevelt (1993). Histology and Histopathology Fine structure of the retinal pigment epithelium of the great horned owl (*Bubo virginianus*).
[4] 10.1007/BF00301820
Response: We agree, this is also seen within our taxa and in cited literature [1]. However, care must be taken when observing melanosomes in the RPE strictly with TEM

as cylindrical melanosomes may appear spherical if they are oriented parallel to the plane of observation. This is evident in our histology of melanosomes in the apical process of RPE cells (Extended Data Fig. 5).

[1] Burgoyne, T., O'Connor, M. N., Seabra, M. C., Cutler, D. F. & Futter, C. E. Regulation of melanosome number, shape and movement in the zebrafish retinal pigment epithelium by OA1 and PMEL. J. Cell. Sci. 128, 1400–1407 (2015). https://doi.org/10.1242/jcs.164400

Comment 7: Lines 362-365: This is a very intriguing subject!! Better yet, it would be nice to evaluate if they could be older by analysing the (four) eyes of early vertebrates. Cool finding!
Response: We thank the reviewer for their appraisal and are excited to see where these methods can be applied in future studies, including in testing interpretations of eye anatomy in early vertebrate fossils. We have now included discussion of evidence for melanosomes from the Cambrian, highlighting the potential to extend these phylogenetic analyses further into deep vertebrate evolutionary history (L443–445).

Comment 8: Line 375: Perhaps it was just nature experimenting with different approaches. It's not surprising that these animals became extinct...
Response: We have amended the text to incorporate the reviewer's suggestion (L452–453).

Comment 9: Lines 380-381: Not necessarily. What about the hypothesis of melanosome alteration through ageing? Some studies have demonstrated that, at least in humans, eye melanosomes shrink with age. This is because new melanosomes are not synthesised de novo, since eye melanosomes are created only during embryonic development [1-3]. Therefore, the question that arises would be relative to the age of the fish sampled. Aside from that, just food for thought, one should consider that melanosome size and density diminish in some regions of the RPE [4]. Consequently, the apparent reduction of microbody size in fossils (from the Mazon Creek) could be biased as a direct result of this sampled area.
Refs:
[1] 10.1111/j.1468-2494.2010.00580_5.x
[2] 10.1111/pcmr.12697
[3] 10.3390/ijms20153739
[4] Schmidt, S.Y., & Peisch, R.D. (1986). Melanin concentration in normal human retinal pigment epithelium.
Response: We thank the reviewer for raising this point, as it is important to address.

As the reviewer correctly points out, ocular pigment epithelial melanosomes are not regenerated during life [1]. On the other hand, melanin <u>concentration</u> in the eye decreases with age [2, 3]. The reference provided by the reviewer does not discuss changes in melanosome size or geometry [1]. In fact, studies of human ocular tissues explicitly report a lack of evidence for age-related change in the size or shape of ocular melanosomes [4]. The revised manuscript includes a brief discussion of this point and additional consideration of broader taphonomic and ecological factors (L462–466)

In contrast, melanosomes in human hair increase in size with age [5], but these melanosomes are continuously regenerated throughout life. Consequently, the observed changes in human hair melanosomes likely reflect the production of newly formed melanosomes, rather than morphological modification of existing melanosomes (L327–333). As the reviewer's comment refers specifically to ocular melanosomes, we do not consider age-related changes in hair melanosomes to be directly comparable.

We acknowledge, however, that we cannot exclude the possibility that specimen age is a potential bias in our dataset. It was not possible to determine the age of all fish specimens used in our study, but we ensured that all extant specimens had reached sexual maturity, to reduce potential age-related bias. We have amended the manuscript to included additional information and discussion of this point in the revised Methods (L766) and Discussion (L327–333 and 462–466).

[1] Lopes, V. S., Wasmeier, C., Seabra, M. C. & Futter, C. E. Melanosome maturation defect in Rab38-deficient retinal pigment epithelium results in instability of immature melanosomes during transient melanogenesis. Molecular Biology of the Cell 18, 3914–3927 (2007). https://doi.org/10.1091/mbc.e07-03-0268
[2] Schmidt, S.Y., & Peisch, R.D. (1986). Melanin concentration in normal human retinal pigment epithelium.
[3] Sarna, T. et al. Loss of melanin from human RPE with aging: possible role of melanin photooxidation. Experimental Eye Research 76, 89–98 (2003). https://doi.org/10.1016/s0014-4835(02)00247-6
[4] Hong, L. et al. Age-dependent photoionization thresholds of melanosomes and lipofuscin isolated from human retinal pigment epithelium cells. Photochem Photobiol 82, 1475–1481 (2006). https://doi.org/10.1562/2006-03-14-RA-846
[5] Itou, T. Morphological changes in hair melanosomes by aging. Pigment Cell Melanoma Res. 31, 630–635 (2018). https://doi.org/10.1111/pcmr.12697

Comment 10: Line 384: Albeit not wrong, I feel this excerpt is a little bolder because vertebrate visual systems imply the preservation of other structures, such as photoreceptors and ganglia. However, it is undeniable that the importance of this study lies in the strong evidence it provides for eye melanisation (and its relative properties) among fish.
Response: Agreed. Text has been amended. (L470–471).

Comment 11: Lines 411-417: This paragraph is repeated, though the next one shows more information.
Response: Agreed. Repeated text removed.

Comment 12: Line 431: Delete the space.
Response: Agreed.

Comment 13: Lines 433-434: Trying to understand why a group of animals doesn't use a particular evolutionary strategy is misguided, as it would imply an idea of directed

evolution. In other words, things in nature happen arbitrarily, without purpose or direction. Therefore, trying to understand is merely a mental exercise.
Response: Agreed. Text deleted.

Comment 14: Lines 444-445: What about dwelling in shallow bodies of water?
Response: Agreed. Text has been amended (L522–525).

Comment 15: Lines 448-452: Just to add. What about the hypothesis of signalling among these animals or sexual selection? I guess that the enhancement of the visual system would favour Chondrichthyes to communicate or better identify sexual partners [1]. Taking into consideration these aspects could also modulate the evolution of visual traits (in addition to increasing diversity of melanosomes) without requiring the explanations of constant changing of light levels.
Refs:
[1] 10.1007/s10641-023-01425-x
Response: Agreed. Text has been amended to incorporate these points (L528–530)

Comment 16: Lines 690-692: Do all dark regions preserve melanosomes?
Response: All examined dark regions correspond to areas of melanosomes and/or mouldic melanosomes. The original manuscript included a reference to this feature, but we have revised the text to clarify (L815–816 and L900–903).

Comment 17: Lines 693-694: Just out of curiosity, were the fossils examined in their entirety? I mean, were they placed directly under a SEM? Why not collect samples by extracting small fragments (<1 mm)?
Response: All fossil specimens were analysed without sputter-coating. Instead, charging was mitigated by wrapping each specimen in aluminium foil, leaving only a very small region, 2–3 mm wide, exposed for SEM imaging at high vacuum. Destructive sampling was not permitted by the museum curators (L866–873).

Comment 18: Line 822: Has this study been approved by an ethics committee? If yes, then it would be nice to put in brackets the number of the protocol (if it exists).
Response: Euthanasia of lungfish was approved by the UCC Animal Experimentation Ethics Committee (approval number: UCC-AEEC-23/001; amendment 10/07/2023) and was performed by Luke Harman (School of BEES; HPRA Individual Authorisation AE19130/I087). This information has now been made more clear in the Methods section (L785–787).
